# Synthesis and Biological Evaluation of New Dihydrofuro[3,2-*b*]piperidine Derivatives as Potent *α*-Glucosidase Inhibitors

**DOI:** 10.3390/molecules29051179

**Published:** 2024-03-06

**Authors:** Haibo Wang, Xiaojiang Huang, Yang Pan, Guoqing Zhang, Senling Tang, Huawu Shao, Wei Jiao

**Affiliations:** 1 Natural Products Research Centre, Chengdu Institute of Biology, Chinese Academy of Sciences, Chengdu 610041, China; wanghb616@163.com (H.W.); huangxiaojiang22@mails.ucas.ac.cn (X.H.); 18685560668@163.com (Y.P.); guoqingzhanga@outlook.com (G.Z.); tangsl@cib.ac.cn (S.T.); shaohw@cib.ac.cn (H.S.); 2 University of Chinese Academy of Sciences, Beijing 100049, China; 3 Zhejiang Hongyuan Pharmaceutical Co., Ltd., Linhai 317016, China

**Keywords:** dihydrofuro[3,2-*b*]piperidine, iminosugar, *C*-glycosides, glucosidase, inhibitors

## Abstract

Inhibition of glycoside hydrolases has widespread application in the treatment of diabetes. Based on our previous findings, a series of dihydrofuro[3,2-*b*]piperidine derivatives was designed and synthesized from D- and L-arabinose. Compounds **32** (IC_50_ = 0.07 μM) and **28** (IC_50_ = 0.5 μM) showed significantly stronger inhibitory potency against *α*-glucosidase than positive control acarbose. The study of the structure–activity relationship of these compounds provides a new clue for the development of new *α*-glucosidase inhibitors.

## 1. Introduction

**Figure 1:**
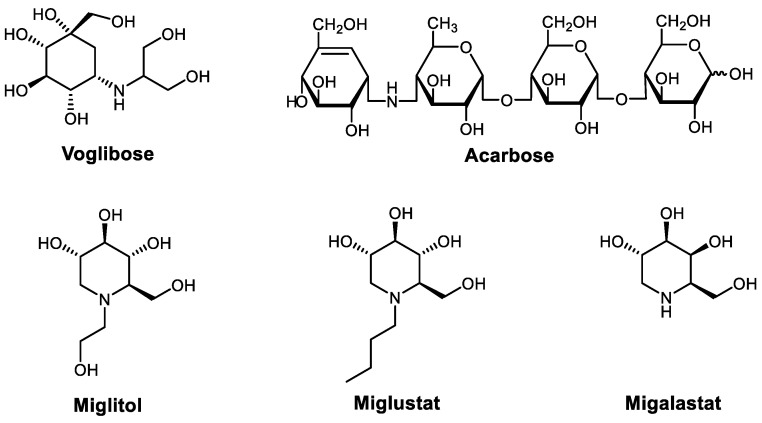
The structure of *α*-glucosidase inhibitors and polyhydroxypiperidines approved by the FDA.

Approximately 422 million people suffer from diabetes worldwide [1], more than 95% of which are type 2 diabetes [2]. It is currently clear that aggressive control of hyperglycemia in patients with type 2 diabetes can attenuate the development of chronic complications such as nephropathy and retinopathy [3,4]. To date, suppressing hyperglycemia is one of the effective approaches for the therapy of type 2 diabetes, which includes reducing gut glucose absorption [5,6,7]. Therefore, inhibition of α-glucosidase, an enzyme located in the small intestine of the human body, which transforms oligosaccharides or glycoconjugates into monosaccharides, is a method of choice to control elevated glucose levels in blood [8,9]. Furthermore, *α*-glucosidase inhibitors also offer other benefits, such as reducing triglyceride levels [10] and post-prandial insulin levels [11]. In fact, *α*-glucosidase inhibitors like acarbose [12], voglibose [13], and miglitol [14] effectively compensate for defective early-phase insulin release by inhibiting post-prandial absorption of monosaccharides, and have been employed for clinical use in the management of type 2 diabetes [15] (Figure 1). However, the incidence of side effects of the inhibitors mentioned above is common, such as gastrointestinal problems [16]. Thus, the novel generation of more selective and potent *α*-glucosidase inhibitors has been consistently required.

Polyhydroxypiperidines, one of the typical iminosugars, are glycomimetics in which the endocyclic oxygen on the hemiacetal ring is replaced by nitrogen [17,18]. As such, they are often competitive inhibitors of enzymes that act on sugar substrates, and several typically successful examples are Miglitol (*N*-hydroxyethyl-1-deoxynojirimycin), Miglustat (*N*-butyl-1-deoxynojirimycin), and Migalastat (1-deoxygalactonojirimycin), which have been approved by the FDA for the treatment of type 2 diabetes [19], Gaucher’s disease [20], Niemann–Pick disease Type C [21], and Fabry disease [22] through showing effects on receptors of intestinal *α*-glucosidases, glucosylceramide synthase [23], and ER (endoplasmic reticulum) *α*-glucosidases I and II [24], respectively (Figure 1).

**Figure 2:**
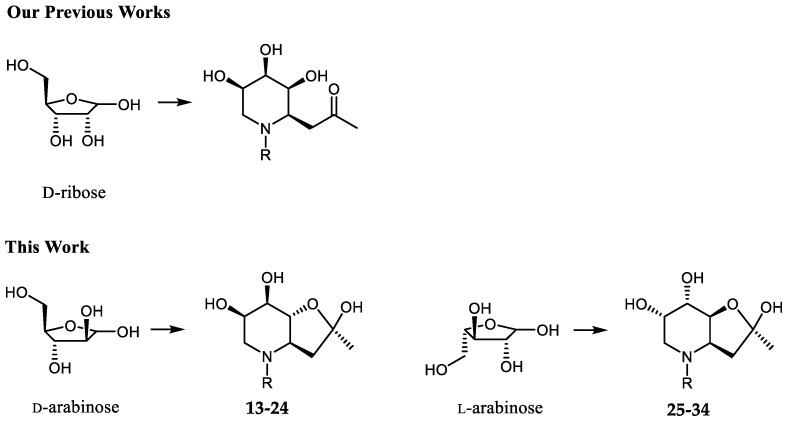
The synthetic route and final products of previous works and the current work.

During the past few years, the synthesis method of hydrofuro[3,2-*b*]piperidine was established by our group [25]. We further developed a series of *N*-substituted iminosugars from D-ribose, and some showed strong inhibitory potency against *α*-glucosidase (Figure 2) [26]. Previous reports have shown that the alteration of chiral centers in the glycomimetics can markedly influence the inhibition [27,28,29]. Therefore, iminosugar derivatives with different hydroxyl configurations should be focused on and investigated to determine the activity to the enzyme target. Furthermore, for these iminosugar derivatives, locked bicyclic glycomimetics are one effective strategy that has attracted particular interest [30,31,32,33]. These conformational locked molecules show excellent performance in terms of the inhibitory effect of glycosidase [34,35,36,37]. The above results inspired us to further investigate the potential of hydrofuro[3,2-*b*]piperidine with different configurations and a locked fused ring system. Herein, we extend the compound library from D-and L-arabinose to obtain a series of arabino-configured Dihydrofuro[3,2-*b*]piperidine derivatives with bicyclic skeletons, resulting in more potent inhibitors of *α*-glucosidases (Figure 2).

## 2. Results and Discussion

### 2.1. Chemistry

**Scheme 1:**
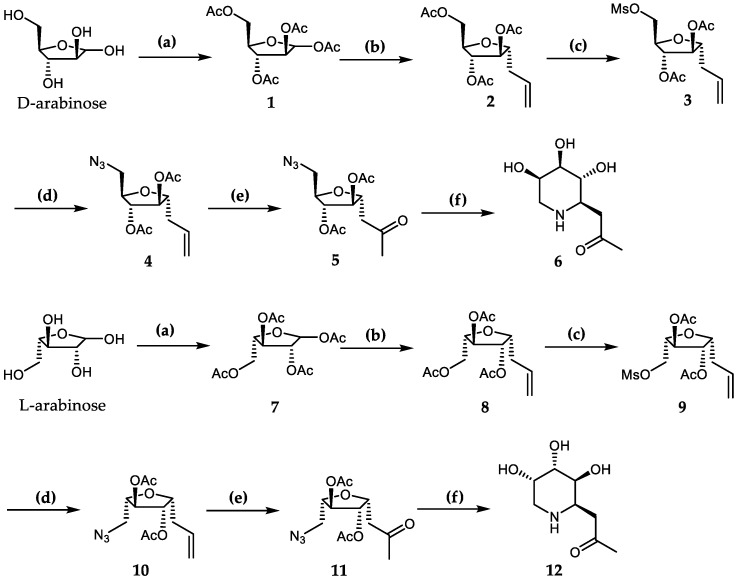
Synthesis of compounds **6** and **12**. Reagents and conditions: (**a**) 1. MeOH, CH_3_COCl, 2.5 h; 2. Ac_2_O, Pridine(Py), overnight; 3. HOAc, Ac_2_O, H_2_SO_4_, 8 h. (**b**) MeCN, Allyl-TMS, TMSOTf. (**c**) 1. MeONa, MeOH, 1 h; 2. Py, MsCl, then Ac_2_O. (**d**) NaN_3_, DMF, 8 h. (**e**) Acetone/H_2_O = 4:1, Hg(OAc)_2_, Jones reagent, 5 h. (**f**) 1. Pd/C, H_2_, MeOH; 2. MeONa, MeOH.

The intermediates **6** and **12** were synthesized from D-arabinose and L-arabinose, respectively (Scheme 1). Global acetylation of D-arabinose or L-arabinose, followed by allylation of the C1 position with TMS-Allyl, resulted in the *C*-glycosides **2** and **8** [38]. Selective deprotection of 5-*O*-acetyl and mesylation of the naked hydroxyl yielded mesylates **3** and **9**. By nucleophilic substitution, mesylates **3** and **9** were reacted with NaN_3_ afforded 5-azido-*C*-ribosides **4** and **10** [39]. The reduction of the azido group to amine was performed after the terminal oxidation of olefin, which was immediately subject to saturated NaOMe in methanol at room temperature overnight. After purifying the crude on silica gel flash column chromatography, compounds **6** and **12** were acquired [40].

**Scheme 2:**
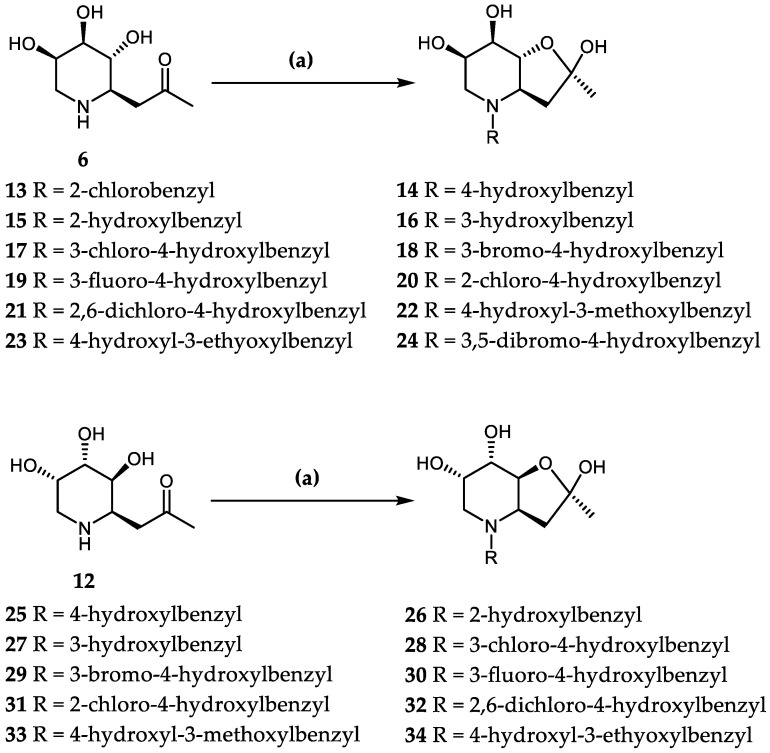
Synthesized Dihydrofuro[3,2-*b*]piperidine derivatives. Reagents and conditions: (**a**) RCHO, NaBH(OAc)_3_, MeOH.

Various benzaldehydes with phenolic hydroxyl groups were used to attach to the synthetic intermediates through reductive amination, resulting in the formation of Dihydrofuro[3,2-*b*]piperidine derivatives **13**–**34**. We observed that the formation of the hemiketal in the final products was attributed to the aldol condensation between the carbonyl group and the adjacent hydroxyl group. Finally, Dihydrofuro[3,2-*b*]piperidine derivatives with *α*-methyl groups on furan rings were produced (Scheme 2).

### 2.2. Structure–Activity Relationship

The inhibitory activities of Dihydrofuro[3,2-*b*]piperidine derivatives **13**–**34** were tested against *α*-glucosidase from yeast [41]. The IC_50_ values are displayed in Table 1. Compounds **6**, **12**, **15**, **16**, **26**, **33** and **34** showed poor inhibitory potency (IC_50_ > 20.0 μM). L-arabino-configured compound **32**, which contains an *N*-substituted 2,6-dichloro-4-hydroxylbenzyl group, exhibited the most potent activity against *α*-glucosidase compared to others. Remarkably, compound **32** showed 30 times stronger activity (IC_50_ = 0.07 μM) than the positive control acarbose (IC_50_ = 2.0 μM), stronger than all compounds reported by us previously [25]. Compound **28** equipped with a 3-chloro-4-hydroxylbenzyl suggested fourfold higher potency (IC_50_ = 0.5 μM) than the positive control acarbose. D-arabino-configured compound **21** (IC_50_ = 0.91 μM), bearing the same *N*-substituted side chain, also showed a significant effect. Although there were different configurations on the piperidine, it is worth noting that compounds with *N*-substituted 3-chloro-4-hydroxylbenzyl and 2,6-dichloro-4-hydroxylbenzyl groups all showed excellent inhibitory effects to *α*-glucosidase [26]. It was revealed that these two *N*-substituted groups are the key factors influencing the activities.

Introducing a hydroxyl into the C2 or C3-position of the phenyl for D-arabino-configured derivatives reduced inhibitory potency significantly, such as compounds **15** (IC_50_ > 20 μM) and **16** (IC_50_ > 20 μM) compared to compound **14** (IC_50_ = 10.0 μM), but this principle could not be found in L-arabino-configured derivatives, such as compounds **26** (IC_50_ > 20 μM) and **27** (IC_50_ = 3.7 μM) compared to compound **25** (IC_50_ = 8.3 μM). However, the installation of a chlorine atom at the C2-position of the 4-hydroxylbenzyl group improved inhibitory activity to the enzyme. For example, compounds **20** (IC_50_ = 4.4 μM) and **31** (IC_50_ = 1.8 μM) showed higher inhibitory activity than compounds **14** and **25**, respectively. Two chlorine atoms located at the C2- and C6-position of 4-hydroxylbenzyl group gave the best result, according to the activity of compounds **21** and **32**. Meanwhile, the location of the halogen atom at the C3-position of the 4-hydroxylbenzyl group, including compounds **17**–**19**, **24**, **29,** and **30**, generally showed slight enhancement for the inhibitory activity, except compound **28**. Introducing an alkoxyl at the C3-position severely weakened the inhibition potency when compounds **22**, **23**, **33**, and **34** were compared to their unsubstituted 4-hydroxyl derivatives **14** and **25**, respectively. Thus, the substituted pattern of the *N*-benzyl side chain has a significant influence on the inhibitory potency of the *α*-glucosidase. On the whole, L-arabino-configured compounds exhibited more potent activities by comparison with the corresponding compounds synthesized from D-arabinose with the same *N*-substituted group, except compounds **26**, **33**, and **34**. This result suggests that the configuration of the hydroxyl groups in the *N*-heterocycle has a dramatic effect on the inhibitory potency of derivatives to the *α*-glucosidase.

In glucose metabolism, the oligosaccharides from α-amylase digestion are finally hydrolyzed to monosaccharides by α-glucosidases at the brush border of enterocytes [42]. Four α-glucosidases are involved in digestion of starch in humans, including maltase-glucoamylase (MGAM (EC number: 3.2.1.20 and EC number: 3.2.1.3)) and sucrose-isomaltase (SI (EC number: 3.2.1.48 and EC number: 3.2.1.10)) [42,43]. These enzymes can be divided into an N-terminal subunit (MGAM-N and SI-N) and a C-terminal subunit (MGAM-C and SI-C) with duplicated catalytic centers. As an efficient drug target for type 2 diabetes, human MGAM plays a crucial role, and MGAM (EC number: 3.2.1.20) is the most active of the four α-glucosidases [42]. All subunits exhibit similar activities, but different substrate specificities [43].

To elucidate the binding modes of the newly synthesized **28**, and **32**, as well as their interactions with *α*-glucosidase, induced fit docking simulations (IFD) were performed considering the flexibility of ligands and proteins simultaneously. Crystal structures of three available human α-glucosidase subunits, 2QMJ (N-terminal of MGAM, EC number: 3.2.1.20) [44], 3TOP (C-terminal of MGAM, EC number: 3.2.1.20) [43], and 3LPP (N-terminal of SI, EC number: 3.2.1.20) [45], were used as receptors for docking [46,47]. The best scoring conformation was utilized to demonstrate the bond formed between the ligand and the binding pocket of the receptor.

**Figure 3:**
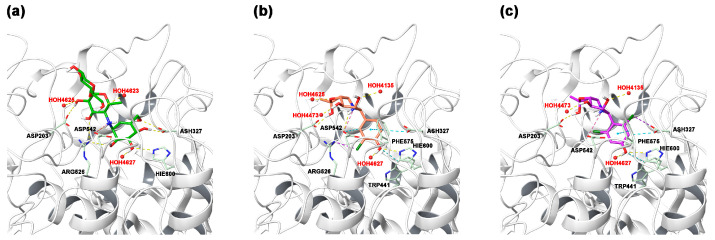
Binding modes of acarbose (**a**) in crystal and predicted binding modes of compounds **28** (**b**) and **32** (**c**) with N-terminal maltase-glucoamylase (PDB ID: 2QMJ). The key residues are represented as a light green stick. The H-bond is shown as a yellow dotted line. The π–π stacking is shown as a blue dotted line. The aromatic H-bond is shown as a light blue dotted line. The halogen bond is shown as a dark purple dotted line. The salt bridge is shown as a pink dotted line. The water molecule in crystal is displayed simply as a red ball.

In the N-terminal MGAM docking model, compounds **28** and **32** were found to accommodate with the same posture and reserve almost all the interactions of acarbose, except additional interactions involving PHE575 and HIE600. As depicted in Figure 3b,c, multiple H-bond interactions were established with HIE600, ASP203, and crystal water molecules; a salt bridge with ASP542; an edge-to-face π-stacking with PHE575; and aromatic H-bonds with ASH327 and TRP441. In addition, a halogen bond with ARG526, an H-bond, and an aromatic H-bond with ASP542 could be found for compound **28**. It is speculated that residues ASP203, ASH327, ASP542, TRP441, PHE575, and HIE600 are essential for the binding of compounds **28** and **32** to N-terminal MGAM.

**Figure 4:**
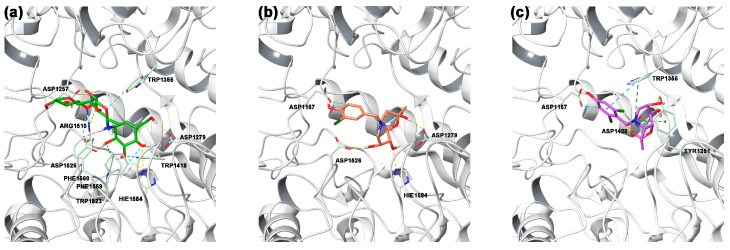
Binding modes of acarbose (**a**) in crystal and predicted binding modes of compounds **28** (**b**) and **32** (**c**) with C-terminal maltase-glucoamylase (PDB ID: 3TOP). The key residues are represented as a light green stick. The H-bond is shown as a yellow dotted line. The π–π stacking is shown as a blue dotted line. The aromatic H-bond is shown as a light blue dotted line. The salt bridge is shown as a pink dotted line. The π–cation interaction is shown as a dark green dotted line.

In the C-terminal MGAM docking model, the binding modes of compound **28** and **32** exhibited notable distinctions in comparison to acarbose. The H-bond interactions with ASP1526, HIE1584, and ASP1279, as well as aromatic H-bonds with ASP1157, were observed in the potential binding mode of compound **28** (Figure 4b). Besides an aromatic H-bond with ASP1157, an edge-to-face π-stacking with PHE1355, a π–cation interaction with TYR1251, and the H-bond interactions with ASP1420 and TYR1251 were predicted to be predominant between compound **32** and C-terminal MGAM (Figure 4c).

**Figure 5:**
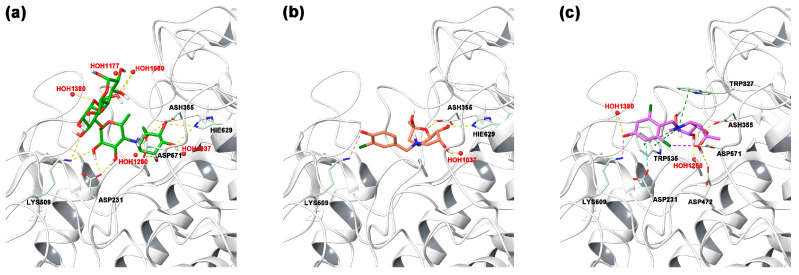
Binding modes of acarbose (**a**) in crystal and predicted binding modes of compounds **28** (**b**) and **32** (**c**) with N-terminal sucrase-isomaltase (PDB: 3LPP). The key residues are represented as a light green stick. The H-bond is shown as a yellow dotted line. The aromatic H-bond is shown as a light blue dotted line. The halogen bond is shown as a dark purple dotted line. The salt bridge is shown as a pink dotted line. The π–cation interaction is shown as a dark green dotted line. The water molecule in crystal is displayed simply as a red ball.

In the N-terminal SI docking model, the most important protein–ligand interactions of compound **28** seemed to be the H-bonds with the ASH355, HIE629 residues, and a crystal water molecule, together with a salt bridge between LYS509 and aromatic oxygen anion of **28** (Figure 5b). Compared to compound **28**, compound **32** was bound to the active pocket with the reverse posture and significantly stronger interactions, stabilized by the H-bond interactions with ASH355, ASP472, and water molecules; an H-bond interaction with ASP231; π–cation interactions with TRP535 and TRP327; a salt bridge with LYS509; and a halogen bond with ASP571 (Figure 5c).

For the high inhibitory effect of **28** and **32**, it can be seen that *N*-substituted 3-chloro-4-hydroxylbenzyl and 2,6-dichloro-4-hydroxylbenzyl groups were hot spots concentrated with multiple forces, such as the halogen bond, π-stacking effect, H-bond, salt bridge, and π–cation interactions. This should be one of the important reasons why these derivatives showed high activities, including previously acquired compounds with similar *N*-substituted groups [26].

### 2.3. Drug Likeness

For further developing this type of inhibitor, the drug likeness characteristics of the synthesized compounds were analyzed in silico. As presented in Table 2, all compounds satisfied Lipinski’s rule, including molecular weight, rotatable bonds, H-bond acceptor, H-bond donor, and Mlog*P*, suggesting that these compounds are likely to have the basic properties of becoming drugs. The Mlog*P* parameter is related to the water solubility, and all molecules showed Mlog*P* ≤ 4.15, indicating that they were appropriately lipophilic for absorption [48]. All compounds **13**–**34** also meet the criteria of Ghose Filter, such as molar refractivity values (40–130) within the scope and effective topological polar surface areas (TPSA ≤ 140 Å^2^) for permeation of the cell membrane [49]. These calculated physicochemical properties provided a preliminary speculation for the probability of developing these compounds into drugs.

## 3. Materials and Methods

### 3.1. Molecular Docking

Shrödinger software (version 13.5, Schrödinger Release 2023-4, Schrödinger, LLC, New York, NY, USA) was used to carry out a docking simulation. Structures were obtained using conformational search (force field: OPLS4) and then optimized at B3LYP/6-31G** with a CPCM solvent model, making use of the Jaguar module. Crystal structures of receptor proteins (PDB ID: 2QMJ, 3TOP, and 3LPP) were prepared after the removal of unnecessary ligands. Water molecules within 5 Å from ligands were removed in the binding site. The active pockets were determined by the Sitemap module and referring to the positions of acarbose and kotalanol in protein crystals. Induced fit docking simulations were performed based on Glide and Prime, which accurately predicted ligand binding modes and concomitant structural changes in the receptor. The optimal docking conformation was identified based on the docking score in the molecular docking pattern map.

### 3.2. General Methods

All reagents were purchased from commercial suppliers and used without further purification unless otherwise noted. Thin layer chromatography was performed using silica gel 60 F254 precoated plates (0.20–0.25 mm thickness) with a fluorescent indicator. Visualization of TLC was achieved by UV light (254 nm) and a typical TLC indication solution (3% sulfuric acid/ethanol solution). Column chromatography was performed on silica gel 300–400 mesh. Melting points were determined with the X-6 (Beijing Fukai Co. Ltd., Beijing, China) melting point apparatus. Optical rotations were measured with a Perkin Elmer M341 Digital Polarimeter. ^1^H and ^13^C NMR (600 and 125 MHz, respectively) spectra were recorded on a Bruker Avance 600 spectrometer. Data are reported as follows: chemical shift, multiplicity (s = singlet, d = doublet, t = triplet, q = quartet, m = multiplet), coupling constants (Hz), and integration. HRESIMS spectra were recorded on a BioTOF-Q mass spectrometer.

### 3.3. General Procedure for α-Glucosidase Inhibition Assay

α-Glucosidase activity was assayed by the method reported, with minor modifications [47,50]. The inhibition rate was determined at 37 °C in 0.067 M Na_2_HPO_4_/NaH_2_PO_4_ buffer (pH 6.8). The reaction mixture contained 150 μL of enzyme solution, 150 μL of inhibitor, and 150 μL of substrate (maltose). The substrate and *α*-glucosidase (Baker’s yeast) were purchased from Sigma-Aldrich Chemical (St. Louis, MO, USA). Both the inhibitor and the substrate were first dissolved in dimethylsulfoxide (DMSO) and then diluted with Na_2_HPO_4_/NaH_2_PO_4_ buffer for a final concentration of DMSO of 5%. The enzymatic reaction was started after incubation of the enzyme (0.04 U/mL) for 10 min in the presence of the inhibitor (five different concentrations) by the addition of the substrate (0.5 mM). The mixture was incubated at 37 °C for 60 min, and the reaction was quenched in boiling water for 10 min. The supernatant was taken to determine the content of glucose by means of the glucose oxidase method using a commercial glucose kit (Sichuan Mike Biotechnology Co., Ltd., Chengdu, China). The absorption at 505 nm was measured immediately and taken as the relative rate for the hydrolysis of the substrate. The blank control (the distilled water instead of sample and enzyme solution in the process) and the negative control (distilled water instead of sample solution in the process) were also prepared. Acarbose was used as the positive control. To calculate the inhibition rate (%), the following formula was used: (control_negative_ − sample)/(control_negative_ − control_blank_) × 100%. The entire experiment was carried out in triplicate.

### 3.4. Synthesis Compounds ***13**–**34***

*(2R, 3aR, 6R, 7R, 7aR)-2,6,7-trihydroxy-2-methyl-4-(2-chlorobenzyl)-3a,7a-Dihydrofuro[3,2-b]piperidine* (**13**) (Figures S21 and S22): A suspension of compound **6** (40 mg, 0.2 mmol) containing activated 4Å molecular sieves in anhydrous methanol (3 mL) was stirred at room temperature for 10 min. After cooling to −20 °C, the corresponding aldehydes (0.6 mmol, 3eq) and NaBH(OAc)_3_ (0.6 mmol, 3eq) were added, and the solution was stirred for 3 h under an argon atmosphere. The reaction solution was concentrated in vacuo and purified by silica gel flash column chromatography (dichloromethane/methanol, 25:1 → 5:1) to afford colorless, syrupy products. Colorless syrup, yield 49%, [α]25D + 35.5 (*c* 0.11, MeOH). ^1^H NMR (600 MHz, DMSO-*d*_6_) *δ* 7.39 (dd, *J* = 7.6, 1.6 Hz, 1H, *H*_Ar_), 7.36–7.29 (m, 2H, 2 × *H*_Ar_), 7.28–7.21 (m, 1H, *H*_Ar_), 3.99 (d, *J* = 13.6 Hz, 1H, O-C*H*), 3.91 (s, 1H, O-C*H*), 3.80–3.70 (m, 3H, O-C*H*, ArC*H*_2_), 2.46 (dd, *J* = 10.6, 4.1 Hz, 1H, NC*H*), 2.38–2.26 (m, 2H, NC*H*_2_), 2.08 (d, *J* = 5.5 Hz, 1H, C*H_2_*), 1.98 (d, *J* = 7.0 Hz, 1H, C*H_2_*), 1.31 (s, 3H, C*H_3_*). ^13^C NMR (151 MHz, Acetone) *δ* 135.2 (*C*_Ar_Cl), 134.2 (*C*_Ar_), 131.5 (*C*_Ar_), 129.6 (*C*_Ar_), 129.0 (*C*_Ar_), 127.1 (*C*_Ar_), 104.5 (O-*C*-OH), 82.1(O-*C*H), 67.7(HO-*C*H), 65.9(HO-*C*H), 60.8(N*C*H), 55.9 (Ar*C*H_2_), 49.9 (N*C*H_2_), 42.4 (*C*H_2_), 25.2 (*C*H_3_). ESI-HRMS: *m*/*z* calcd for C_15_H_20_O_4_NClNa [M + Na]^+^: 336.0973; found: 336.0957.

*(2R, 3aR, 6R, 7R, 7aR)-2,6,7-trihydroxy-2-methyl-4-(4-hydroxylbenzyl)-3a,7a-Dihydrofuro[3,2-b]piperidine* (**14**) (Figures S23 and S24): The synthesized procedure was the same as compound **13**. Colorless syrup, yield 42%, [α]25D +50.0 (*c* 0.13, MeOH). ^1^H NMR (600 MHz, CD_3_OD) *δ* 7.09 (m, 2H, 2 × *H*_Ar_), 6.74 (m, 2 × *H*_Ar_), 4.05–4.00 (m, 2H, 2 × O-C*H*), 3.89(s, 1H, O-C*H*), 3.88–3.70 (m, 2H, ArC*H*_2_), 2.58 (dd, *J* = 10.8, 4.1 Hz, 1H, NC*H*), 2.47 (d, *J* = 13.5 Hz, 1H, NC*H*_2_), 2.32 (t, *J* = 10.9 Hz, 1H, NC*H*_2_), 2.18 (d, *J* = 12.9 Hz, 1H, C*H_2_*), 2.00 (d, *J* = 13.1 Hz, 1H, C*H_2_*), 1.44 (s, 3H, C*H*_3_). ^13^C NMR (151 MHz, CD_3_OD) *δ* 156.7 (*C*_Ar_OH), 130.2 (*C*_Ar_), 127.6 (*C*_Ar_), 127.6 (*C*_Ar_), 114.9 (*C*_Ar_), 114.6 (*C*_Ar_), 105.3 (O-*C*-OH), 82.6(O-*C*H), 67.3(HO-*C*H), 65.8(HO-*C*H), 59.7 (N*C*H), 57.8 (Ar*C*H_2_), 48.4 (N*C*H_2_), 41.5 (*C*H_2_), 24.0 (*C*H_3_). ESI-HRMS: *m*/*z* calcd for C_15_H_21_O_5_N [M + H]^+^: 296.1407; found: 296.1481.

*(2R, 3aR, 6R, 7R, 7aR)-2,6,7-trihydroxy-2-methyl-4-(2-hydroxylbenzyl)-3a,7a-Dihydrofuro[3,2-b]piperidine* (**15**): The synthesized procedure was the same as compound **13**. Colorless syrup, yield 41%, [α]25D +42.6 (*c* 0.46, MeOH). ^1^H NMR (600 MHz, CD_3_OD) *δ* 7.11 (m, 2H, 2 × *H*_Ar_), 6.80–6.70 (m, 2H, 2 × *H*_Ar_), 4.23 (s, 1H, O-C*H*), 4.02 (s, 1H, O-C*H*), 3.94 (d, *J* = 12.6 Hz, 2H, O-C*H*, ArC*H*_2_), 3.52 (s, 1H, ArC*H*_2_), 3.20 (d, *J* = 12.7 Hz, 1H, NC*H*), 2.61 (dd, *J* = 29.8, 7.9 Hz, 1H, NC*H*_2_), 2.50 (d, *J* = 13.0 Hz, 1H, NC*H*_2_), 2.44 (t, *J* = 10.5 Hz, 1H, C*H*_2_), 2.34 (t, *J* = 10.8 Hz, 1H, C*H*_2_), 1.44 (s, 3H, C*H*_3_). ^13^C NMR (151 MHz, CD_3_OD) *δ* 154.6 (*C*_Ar_OH), 129.3 (*C*_Ar_), 127.2 (*C*_Ar_), 127.0 (*C*_Ar_), 117.6 (*C*_Ar_), 113.4 (*C*_Ar_), 103.0 (O-*C*-OH), 80.9 (O-*C*H), 78.3 (HO-*C*H), 66.0 (HO-*C*H), 64.5 (N*C*H), 58.6 (Ar*C*H_2_), 53.2 (N*C*H_2_), 47.5 (*C*H_2_), 23.6 (*C*H_3_). ESI-HRMS: *m*/*z* calcd for C_15_H_21_O_5_NNa [M + Na]^+^: 318.1312; found: 318.1322.

*(2R, 3aR, 6R, 7R, 7aR)-2,6,7-trihydroxy-2-methyl-4-(3-hydroxylbenzyl)-3a,7a-Dihydrofuro[3,2-b]piperidine* (**16**) (Figures S25 and S26): The synthesized procedure was the same as compound **13**. Colorless syrup, yield 45%, [α]25D +61.5 (*c* 0.16, MeOH). ^1^H NMR (600 MHz, CD_3_OD) *δ* 7.14–7.10 (m, 1H, *H*_Ar_), 6.78–6.66 (m, 3H, 3×*H*_Ar_), 4.20–3.80 (m, 2H, ArC*H*_2_), 4.07 (d, *J* = 13.1 Hz, 1H, O-C*H*), 3.91–3.80 (m, 2H, 2 × O-C*H*), 2.60 (d, *J* = 7.4 Hz, 1H, NC*H*), 2.46 (d, *J* = 13.4 Hz, 1H, NC*H*_2_), 2.33 (t, *J* = 11.0 Hz, 1H, NC*H*_2_), 2.16 (d, *J* = 9.0 Hz, 1H, C*H*_2_), 2.01 (d, *J* = 11.3 Hz, 1H, C*H*_2_), 1.44 (s, 3H, C*H*_3_). ^13^C NMR (151 MHz, CD_3_OD) *δ* 155.9 (*C*_Ar_OH), 137.0 (*C*_Ar_), 127.7 (*C*_Ar_), 118.5 (*C*_Ar_), 114.2 (*C*_Ar_), 112.7 (*C*_Ar_), 103.8 (O-*C*-OH), 81.1(O-*C*H), 65.8(HO-*C*H), 64.3(HO-*C*H), 58.4 (N*C*H), 56.9 (Ar*C*H_2_), 47.4 (N*C*H_2_), 40.0 (*C*H_2_), 22.6 (*C*H_3_). ESI-HRMS: *m*/*z* calcd for C_15_H_21_O_5_NNa [M + Na]^+^: 318.1312; found: 318.1317.

*(2R, 3aR, 6R, 7R, 7aR)-2,6,7-trihydroxy-2-methyl-4-(3-chloro-4-hydroxylbenzyl)-3a,7a-Dihydrofuro[3,2-b]piperidine* (**17**) (Figures S27 and S28): The synthesized procedure was the same as compound **13**. Colorless syrup, yield 35%, [α]25D +38.0 (*c* 0.10, MeOH). ^1^H NMR (600 MHz, CD_3_OD) *δ* 7.06–7.01 (m, 1H, *H*_Ar_), 6.88 (d, *J* = 8.3 Hz, 1H, *H*_Ar_), 6.84 (d, *J* = 8.2 Hz, 1H, *H*_Ar_), 4.19–3.96 (m, 3H, 3 × O-C*H*), 3.92–3.82 (m, 2H, ArC*H*_2_), 2.56 (dd, *J* = 10.8, 4.1 Hz, 1H, NC*H*), 2.47 (d, *J* = 13.5 Hz, 1H, NC*H*_2_), 2.35 (t, *J* = 10.9 Hz, 1H, NC*H*_2_), 2.16 (d, *J* = 8.1 Hz, 1H, C*H*_2_), 2.03–1.98 (m, 1H, C*H*_2_), 1.44 (s, 3H, C*H*_3_). ^13^C NMR (151 MHz, CD_3_OD) *δ* 152.5 (*C*_Ar_OH), 130.4 (*C*_Ar_), 129.1 (*C*_Ar_), 128.5 (*C*_Ar_), 120.3 (*C*_Ar_Cl), 116.2 (*C*_Ar_), 105.3 (O-*C*-OH), 82.6(O-*C*H), 67.3(HO-*C*H), 65.8(HO-*C*H), 59.7 (N*C*H), 57.2 (Ar*C*H_2_), 48.7 (N*C*H_2_), 41.5 (*C*H_2_), 24.1 (*C*H_3_). ESI-HRMS: *m*/*z* calcd for C_15_H_20_O_5_NClNa [M + Na]^+^: 352.0922; found: 352.0917.

*(2R, 3aR, 6R, 7R, 7aR)-2,6,7-trihydroxy-2-methyl-4-(3-bromo-4-hydroxylbenzyl)-3a,7a-Dihydrofuro[3,2-b]piperidine* (**18**) (Figures S29 and S30): The synthesized procedure was the same as compound **13**. Colorless syrup, yield 46%, [α]25D +37.3 (*c* 0.26, MeOH). ^1^H NMR (600 MHz, CD_3_OD) *δ* 7.08–7.04 (m, 1H, *H*_Ar_), 6.86 (d, *J* = 8.2 Hz, 1H, *H*_Ar_), 6.84 (d, *J* = 8.2 Hz, 1H, *H*_Ar_), 4.05–4.01 (m, 2H, 2 × O-C*H*), 3.96 (s, 1H, O-C*H*), 3.90–3.75 (m, 2H, ArCH_2_), 2.56 (dd, *J* = 10.7, 4.1 Hz, 1H, NC*H*), 2.47 (d, *J* = 13.5 Hz, 1H, NC*H*_2_), 2.31 (t, *J* = 10.9 Hz, 1H, NC*H*_2_), 2.16 (d, *J* = 9.2 Hz, 1H, C*H*_2_), 2.00 (dd, *J* = 13.5, 3.8 Hz, 1H, C*H*_2_), 1.44 (s, 3H, C*H*_3_). ^13^C NMR (151 MHz, CD_3_OD) *δ* 153.6 (*C*_Ar_OH), 133.5 (*C*_Ar_), 129.4 (*C*_Ar_), 129.3 (*C*_Ar_), 115.9 (*C*_Ar_), 109.4 (*C*_Ar_Br), 105.3 (O-*C*-OH), 82.6(O-*C*H), 67.3(HO-*C*H), 65.8(HO-*C*H), 59.7 (N*C*H), 57.1 (Ar*C*H_2_), 48.7 (N*C*H_2_), 41.5 (*C*H_2_), 24.1 (*C*H_3_). ESI-HRMS: *m*/*z* calcd for C_15_H_20_O_5_NBrNa [M + Na]^+^: 396.0417; found: 396.0415.

*(2R, 3aR, 6R, 7R, 7aR)-2,6,7-trihydroxy-2-methyl-4-(3-fluoro-4-hydroxylbenzyl)-3a,7a-Dihydrofuro[3,2-b]piperidine* (**19**) (Figures S31 and S32): The synthesized procedure was the same as compound **13**. Colorless syrup, yield 40%, [α]25D +43.9 (*c* 0.19, MeOH). ^1^H NMR (600 MHz, CD_3_OD) *δ* 7.10–6.75 (m, *3*H, 3×*H*_Ar_), 4.03 (t, *J* = 3.2 Hz, 1H, O*H*), 3.90–3.85 (m, 2H, 2 × O-C*H*), 3.75–3.60(m, 2H, ArC*H*_2_), 2.56 (dd, *J* = 10.7, 4.0 Hz, 1H, NC*H*), 2.47 (d, *J* = 13.5 Hz, 1H, NC*H*_2_), 2.34 (t, *J* = 10.9 Hz, 1H, NC*H*_2_), 2.15 (d, *J* = 9.3 Hz, 1H, C*H*_2_), 2.01 (dd, *J* = 13.5, 3.9 Hz, 1H, C*H*_2_), 1.44 (s, 3H, C*H*_3_). ^13^C NMR (151 MHz, CD_3_OD) *δ* 151.4 (*C*_Ar_F), 144.3 (*C*_Ar_OH), 128.8 (*C*_Ar_), 125.1 (*C*_Ar_), 117.3 (*C*_Ar_), 116.3 (*C*_Ar_), 105.3 (O-*C*-OH), 82.6(O-*C*H), 67.3(HO-*C*H), 65.8(HO-*C*H), 59.7 (N*C*H), 57.4 (Ar*C*H_2_), 48.6 (N*C*H_2_), 41.5 (*C*H_2_), 24.1 (*C*H_3_). ESI-HRMS: *m*/*z* calcd for C_15_H_20_O_5_NFNa [M + Na]^+^: 336.1218; found: 336.1211.

*(2R, 3aR, 6R, 7R, 7aR)-2,6,7-trihydroxy-2-methyl-4-(2-chloro-4-hydroxylbenzyl)-3a,7a-Dihydrofuro[3,2-b]piperidine* (**20**) (Figures S33 and S34): The synthesized procedure was the same as compound **13**. Colorless syrup, yield 44%, [α]25D +30.6 (*c* 0.11, MeOH). ^1^H NMR (600 MHz, CD_3_OD) *δ* 7.16 (d, *J* = 8.4 Hz, 1H, *H*_Ar_), 6.83 (d, *J* = 2.4 Hz, 1H, *H*_Ar_), 6.72 (dd, *J* = 8.5, 2.4 Hz, 1H, *H*_Ar_), 4.08–4.01 (m, 2H, 2 × O-C*H*), 3.96 (s, 1H, O-C*H*), 3.91–3.70 (m, 2H, ArC*H*_2_), 2.53 (dd, *J* = 10.7, 3.9 Hz, 1H, NC*H*), 2.48 (d, *J* = 13.5 Hz, 1H, NC*H*_2_), 2.37 (t, *J* = 10.9 Hz, 1H, NC*H*_2_), 2.16 (d, *J* = 12.8 Hz, 1H, C*H*_2_), 2.01 (d, *J* = 4.0 Hz, 1H, C*H*_2_), 1.42 (s, 3H, C*H*_3_). ^13^C NMR (151 MHz, CD_3_OD) *δ* 157.9 (*C*_Ar_OH), 134.7 (*C*_Ar_Cl), 132.5 (*C*_Ar_), 124.7 (*C*_Ar_), 115.9 (*C*_Ar_), 114.0 (*C*_Ar_), 105.0 (O-*C*-OH), 82.6(O-*C*H), 67.3(HO-*C*H), 65.8(HO-*C*H), 60.6 (Ar*C*H_2_), 55.5 (N*C*H_2_), 48.7 (N*C*H), 41.5 (*C*H_2_), 24.2 (*C*H_3_). ESI-HRMS: *m*/*z* calcd for C_15_H_21_O_5_NCl [M + H]^+^: 330.1103; found: 330.1104.

*(2R, 3aR, 6R, 7R, 7aR)-2,6,7-trihydroxy-2-methyl-4-(2,6-dichloro-4-hydroxylbenzyl)-3a,7a-Dihydrofuro[3,2-b]piperidine* (**21**) (Figures S35 and S36): The synthesized procedure was the same as compound **13**. Colorless syrup, yield 31%, [α]25D +6.78 (*c* 0.12, MeOH). ^1^H NMR (600 MHz, CD_3_OD) *δ* 6.84 (s, 1H, *H*_Ar_), 6.80 (d, *J* = 3.6 Hz, 1H, *H*_Ar_), 4.11 (d, *J* = 12.7 Hz, 1H, O-C*H*), 4.04–4.01 (m, 1H, O-C*H*), 3.95 (s, 1H, O-C*H*), 3.85–3.40 (m, 2H, ArC*H*_2_), 2.62 (t, *J* = 10.8 Hz, 1H, NC*H*), 2.50 (d, *J* = 5.7 Hz, 1H, NC*H*_2_), 2.43 (dd, *J* = 10.6, 4.1 Hz, 1H, NC*H*_2_), 2.21 (d, *J* = 3.1 Hz, 1H, C*H*_2_), 1.98(m, 1H, C*H*_2_), 1.39 (s, 3H, C*H*_3_). ^13^C NMR (151 MHz, CD_3_OD) *δ* 157.8 (*C*_Ar_OH), 136.9 (*C*_Ar_Cl), 136.9 (*C*_Ar_Cl), 122.7 (*C*_Ar_),115.5 (*C*_Ar_), 115.4 (*C*_Ar_), 104.7 (O-*C*-OH), 82.6(O-*C*H), 67.3(HO-*C*H), 66.0(HO-*C*H), 61.6 (N*C*H), 53.7 (Ar*C*H_2_), 48.7 (N*C*H_2_), 41.3 (*C*H_2_), 20.1 (*C*H_3_). ESI-HRMS: *m*/*z* calcd for C_15_H_21_O_5_NCl_2_ [M + H]^+^: 364.0719; found: 364.0713.

*(2R, 3aR, 6R, 7R, 7aR)-2,6,7-trihydroxy-2-methyl-4-(4-hydroxyl-methoxylbenzyl)-3a,7a-Dihydrofuro[3,2-b]piperidine* (**22**) (Figures S37 and S38): The synthesized procedure was the same as compound **13**. Colorless syrup, yield 47%, [α]25D +36.4 (*c* 0.06, MeOH). ^1^H NMR (600 MHz, CD_3_OD) *δ* 6.83–6.70 (m, 3H, 3×*H*_Ar_), 4.05 (d, *J* = 11.9 Hz, 2H, 2 × O-C*H*), 3.97 (s, 1H, O-C*H*), 3.85–3.75 (m, 2H, ArC*H*_2_), 3.83 (s, 3H, OC*H*_3_), 2.61 (dd, *J* = 10.8, 4.0 Hz, 1H, NC*H*), 2.50 (d, *J* = 13.5 Hz, 1H, NC*H*_2_), 2.34 (t, *J* = 11.0 Hz, 1H, NC*H*_2_), 2.21 (d, *J* = 6.6 Hz, 1H, C*H*_2_), 2.01 (dd, *J* = 13.5, 3.8 Hz, 1H, C*H*_2_), 1.45 (s, 3H, C*H*_3_). ^13^C NMR (151 MHz, CD_3_OD) *δ* 147.8 (*C*_Ar_OCH_3_), 145.9 (*C*_Ar_OH), 128.3 (*C*_Ar_), 121.8(*C*_Ar_), 114.7(*C*_Ar_), 112.3(*C*_Ar_), 105.8 (O-*C*-OH), 82.6 (O-*C*H), 67.3 (HO-*C*H), 66.5(HO-*C*H), 65.7 (N*C*H), 58.2 (Ar*C*H_2_), 54.9 (O*C*H_3_), 48.7 (N*C*H_2_), 24.0 (*C*H_2_), 20.1 (C*C*H_3_). ESI-HRMS: *m*/*z* calcd for C_16_H_23_O_6_NNa [M + Na]^+^: 348.1418; found: 348.1427.

*(2R, 3aR, 6R, 7R, 7aR)-2,6,7-trihydroxy-2-methyl-4-(4-hydroxyl-3-ethyoxylbenzyl)-3a,7a-Dihydrofuro[3,2-b]piperidine* (**23**) (Figures S39 and S40): The synthesized procedure was the same as compound **13**. Colorless syrup, yield 50%, [α]25D +40.6 (*c* 0.11, MeOH). ^1^H NMR (600 MHz, CD_3_OD) *δ* 6.80 (s, 1H, phO*H*_Ar_), 6.77–6.65 (m, 3H, 3×*H*_Ar_), 4.11–3.99 (m, 5H, OC*H*_2,_ 3 × O-C*H*), 3.90–3.75 (m, 2H, ArC*H*_2_), 2.60 (dd, *J* = 10.6, 4.0 Hz, 1H, NC*H*), 2.31 (m, 1H, NC*H*_2_), 2.22–2.16 (m, 1H, NC*H*_2_), 2.02–1.95 (m, 2H, CC*H*_2_), 1.45 (s, 3H, CC*H*_3_), 1.41 (t, *J* = 9.0 Hz, 3H, CH_2_C*H*_3_). ^13^C NMR (151 MHz, CD_3_OD) *δ* 146.8 (*C*_Ar_OEt), 146.1 (*C*_Ar_OH), 128.3 (*C*_Ar_), 121.8 (*C*_Ar_), 114.6 (*C*_Ar_), 113.6 (*C*_Ar_), 105.3 (O-*C*-OH), 82.6 (O-*C*H), 67.3 (HO-*C*H), 65.8 (HO-*C*H), 64.2 (O*C*H_2_), 59.7(N*C*H), 58.2 (Ar*C*H_2_), 48.7 (N*C*H_2_), 48.2 (C*C*H_2_), 24.1 (C*C*H_3_), 13.7 (CH_2_*C*H_3_). ESI-HRMS: *m*/*z* calcd for C_17_H_25_O_6_NNa [M + Na]^+^: 362.1574; found: 362.1579.

*(2R, 3aR, 6R, 7R, 7aR)-2,6,7-trihydroxy-2-methyl-4-(3,5-dibromo-4-hydroxyl-benzyl)-3a,7a-Dihydrofuro[3,2-b]piperidine* (**24**) (Figures S41 and S42): The synthesized procedure was the same as compound **13**. Colorless syrup, yield 37%, [α]25D +18.6 (*c* 0.10, MeOH). ^1^H NMR (600 MHz, CD_3_OD) *δ* 7.44 (s, 1H, *H*_Ar_), 7.40 (s, 1H, *H*_Ar_), 4.02 (dd, *J* = 12.0, 8.5 Hz, 2H, 2 × O-C*H*), 3.96 (s, 1H, O-C*H*), 3.90–3.80 (m, 2H, ArC*H*_2_), 2.55 (d, *J* = 10.7 Hz, 1H, NC*H*), 2.46 (m, 1H, NC*H*_2_), 2.36 (t, *J* = 10.8 Hz, 1H, NC*H*_2_), 2.18 (s, 1H, *C*H_2_), 2.00 (d, *J* = 4.0 Hz, 1H, *C*H_2_), 1.45 (s, 3H, *C*H_3_). ^13^C NMR (151 MHz, CD_3_OD) *δ* 150.7(*C*_Ar_OH), 132.8 (*C*_Ar_), 132.1(*C*_Ar_), 131.4(*C*_Ar_), 111.0 (*C*_Ar_Br), 110.8 (*C*_Ar_Br), 105.3 (O-*C*-OH), 82.5 (O-*C*H), 67.3 (HO-*C*H), 65.8 (HO-*C*H), 59.6(N*C*H), 56.6 (Ar*C*H_2_), 48.6(N*C*H_2_), 41.4 (*C*H_2_), 24.1(*C*H_3_). ESI-HRMS: *m*/*z* calcd for C_15_H_20_O_5_NBr_2_[M + H]^+^: 451.9703; found: 451.9698.

*(2R, 3aR, 6S, 7S, 7aS)-2,6,7-trihydroxy-2-methyl-4-(4-hydroxylbenzyl)-3a,7a-di-hydrofuro[3,2-b]piperidine* (**25**) (Figures S43 and S44): A suspension of compound **12** (40 mg, 0.2 mmol) containing activated 4Å molecular sieves in anhydrous methanol (3 mL) was stirred at room temperature for 10 min. After cooling to −20 °C, the corresponding aldehydes (0.6 mmol, 3eq) and NaBH(OAc)_3_ (0.6 mmol, 3eq) were added, and the solution was stirred for 3 h under an argon atmosphere. The reaction solution was concentrated in vacuo and purified by silica gel flash column chromatography (dichloromethane/methanol, 25:1 → 5:1) to afford **25**. Colorless syrup, yield 45%, [α]25D−16.0 (*c* 0.10, MeOH). ^1^H NMR (600 MHz, CD_3_OD) *δ* 7.08 (d, *J* = 8.3 Hz, 2H, 2 × *H*_Ar_), 6.74 (dd, *J* = 11.3, 2.9 Hz, 2H, 2 × *H*_Ar_), 4.06–4.00 (m, 2H, 2 × O-C*H*), 3.96 (s, 1H, O-C*H*), 3.89–3.75 (m, 2H, ArC*H*_2_), 2.59 (dd, *J* = 10.9, 4.2 Hz, 1H, NC*H*), 2.49 (d, *J* = 13.5 Hz, 1H, NC*H*_2_), 2.31 (t, *J* = 8.4 Hz, 1H, NC*H*_2_), 2.20 (d, *J* = 8.5 Hz, 1H, C*H*_2_), 2.04–1.98 (m, 1H, C*H*_2_), 1.44 (s, 3H, C*H*_3_). ^13^C NMR (151 MHz, CD_3_OD) *δ* 156.7 (*C*_Ar_OH), 130.2 (*C*_Ar_), 129.9 (*C*_Ar_), 127.5 (*C*_Ar_), 114.9 (*C*_Ar_), 114.6 (*C*_Ar_), 105.2 (O-*C*-OH), 82.6 (O-*C*H), 67.3 (HO-*C*H), 65.8 (HO-*C*H), 59.6 (N*C*H), 57.8 (Ar*C*H_2_), 48.6 (N*C*H_2_), 42.5 (*C*H_2_), 24.0 (*C*H_3_). ESI-HRMS: *m*/*z* calcd for C_15_H_22_O_5_NNa [M + Na]^+^: 296.1492; found: 296.1501.

*(2R, 3aR, 6S, 7S, 7aS)-2,6,7-trihydroxy-2-methyl-4-(2-hydroxylbenzyl)-3a,7a-di-hydrofuro[3,2-b]piperidine* (**26**) (Figures S45 and S46): The synthesized procedure was the same as compound **25**. Colorless syrup, yield 47%, [α]25D−35.0 (*c* 0.10, MeOH). ^1^H NMR (600 MHz, CD_3_OD) *δ* 7.12–7.09 (m, 1H, *H*_Ar_), 6.78–6.75 (m, 2H, 2 × *H*_Ar_), 6.72 (dd, *J* = 8.1, 4.3 Hz, 1H, *H*_Ar_), 4.01 (d, *J* = 3.3 Hz, 1H, O-C*H*), 3.98–3.93 (m, 1H, O-C*H*), 3.92–3.80 (m, 3H, O-C*H*, ArC*H*_2_), 2.61 (dd, *J* = 11.3, 3.8 Hz, 1H, NC*H*), 2.57 (t, *J* = 6 Hz, 1H, NC*H*_2_), 2.51 (d, *J* = 4.8 Hz, 1H, NC*H*_2_), 2.45 (dd, *J* = 17.2, 6.9 Hz, 1H, C*H*_2_), 2.32 (t, *J* = 10.8 Hz, 1H, C*H*_2_), 1.44 (s, 3H, C*H*_3_). ^13^C NMR (151 MHz, CD_3_OD) *δ* 156.1 (*C*_Ar_OH), 130.9 (*C*_Ar_), 128.6 (*C*_Ar_), 123.0 (*C*_Ar_), 118.7 (*C*_Ar_), 114.7 (*C*_Ar_), 104.5 (O-*C*-OH), 82.5 (O-*C*H), 67.7 (HO-*C*H), 66.5 (HO-*C*H), 60.3 (N*C*H), 54.7 (Ar*C*H_2_), 49.0 (N*C*H_2_), 41.5 (*C*H_2_), 25.1 (*C*H_3_). ESI-HRMS: *m*/*z* calcd for C_15_H_22_O_5_N [M + H]^+^: 296.1492; found: 296.1493.

*(2R, 3aR, 6S, 7S, 7aS)-2,6,7-trihydroxy-2-methyl-4-(3-hydroxylbenzyl)-3a,7a-di-hydrofuro[3,2-b]piperidine* (**27**) (Figures S47 and S48): The synthesized procedure was the same as compound **25**. Colorless syrup, yield 48%, [α]25D−32.8 (*c* 0.08, MeOH). ^1^H NMR (600 MHz, CD_3_OD) *δ* 7.15–7.10 (m, 1H, *H*_Ar_), 6.80–6.30 (m, 3H, *H*_Ar_), 4.08–4.02 (m, 2H, 2 × O-C*H*), 3.97 (s, 1H, O-C*H*), 3.90–3.75 (m, 2H, ArC*H*_2_), 2.59 (d, *J* = 4.1 Hz, 1H, NC*H*), 2.47 (d, *J* = 13.5 Hz, 1H, NC*H*_2_), 2.32 (t, *J* = 10.9 Hz, 1H, NC*H*_2_), 2.14 (d, *J* = 9.2 Hz, 1H, C*H*_2_), 2.01 (d, *J* = 9.6 Hz, 1H, C*H*_2_), 1.44 (s, 3H, C*H*_3_). ^13^C NMR (151 MHz, CD_3_OD) *δ* 157.4 (*C*_Ar_OH), 138.5 (*C*_Ar_), 129.2 (*C*_Ar_), 120.0 (*C*_Ar_), 115.6 (*C*_Ar_), 114.1 (*C*_Ar_), 105.3 (O-*C*-OH), 82.6 (O-*C*H), 67.3 (HO-*C*H), 65.8 (HO-*C*H), 59.9 (N*C*H), 58.4 (Ar*C*H_2_), 48.9 (N*C*H_2_), 41.5 (*C*H_2_), 24.1 (*C*H_3_). ESI-HRMS: *m*/*z* calcd for C_15_H_22_O_5_N [M + H]^+^: 296.1492; found: 296.1493.

*(2R, 3aR, 6S, 7S, 7aS)-2,6,7-trihydroxy-2-methyl-4-(3-chloro-4-hydroxylbenzyl)-3a,7a-Dihydrofuro[3,2-b]piperidine* (**28**) (Figures S49 and S50): The synthesized procedure was the same as compound **25**. Colorless syrup, yield 37%, [α]25D−23.7 (*c* 0.19, MeOH). ^1^H NMR (600 MHz, CD_3_OD) *δ* 7.25–7.15 (m, 1H, *H*_Ar_), 7.10–7.00 (m, 1H, *H*_Ar_), 6.90–6.80 (m, 1H, *H*_Ar_), 4.04–4.00 (m, 1H, O-C*H*), 4.00 (s, 1H, O-C*H*), 3.96 (s, 1H, O-C*H*), 3.86–3.65 (m, 2H, ArC*H*_2_), 2.55 (dd, *J* = 10.7, 4.1 Hz, 1H, NC*H*), 2.47 (d, *J* = 13.5 Hz, 1H, NC*H*_2_), 2.30 (t, *J* = 10.9 Hz, 1H, NC*H*_2_), 2.15 (d, *J* = 11.1 Hz, 1H, C*H*_2_), 2.00 (dd, *J* = 13.5, 3.9 Hz, 1H, C*H*_2_), 1.44 (s, 3H, C*H*_3_). ^13^C NMR (151 MHz, CD_3_OD) *δ* 152.5 (*C*_Ar_OH), 130.4 (*C*_Ar_), 128.5 (*C*_Ar_), 128.5 (*C*_Ar_), 120.3 (*C*_Ar_Cl), 116.2 (*C*_Ar_), 105.3 (O-*C*-OH), 82.6 (O-*C*H), 67.3 (HO-*C*H), 65.8 (HO-*C*H), 59.7 (N*C*H), 57.2 (Ar*C*H_2_), 48.6 (N*C*H_2_), 41.5 (*C*H_2_), 24.1 (*C*H_3_). ESI-HRMS: *m*/*z* calcd for C_15_H_21_O_5_NCl [M + H]^+^: 330.1103; found: 330.1102.

*(2R, 3aR, 6S, 7S, 7aS)-2,6,7-trihydroxy-2-methyl-4-(3-bromo-4-hydroxylbenzyl)-3a,7a-Dihydrofuro[3,2-b]piperidine* (**29**) (Figures S51 and S52): The synthesized procedure was the same as compound **25**. Colorless syrup, yield 40%, [α]25D−47.5 (*c* 0.04, MeOH). ^1^H NMR (600 MHz, CD_3_OD) *δ* 7.37 (d, *J* = 2.0 Hz, 1H, *H*_Ar_), 7.06 (dd, *J* = 8.3, 2.0 Hz, 1H, *H*_Ar_), 6.86 (d, *J* = 7.6 Hz, 1H, *H*_Ar_), 4.10–4.00 (m, 2H, O-C*H*), 3.96 (s, 1H, O-C*H*), 3.90–3.75 (m, 2H, ArC*H*_2_), 2.55 (dd, *J* = 10.7, 4.0 Hz, 1H, NC*H*), 2.48 (d, *J* = 13.5 Hz, 1H, NC*H*_2_), 2.30 (t, *J* = 10.9 Hz, 1H, NC*H*_2_), 2.15 (d, *J* = 10.8 Hz, 1H, C*H*_2_), 2.01 (dd, *J* = 13.5, 3.8 Hz, 1H, C*H*_2_), 1.44 (s, 3H, C*H*_3_). ^13^C NMR (151 MHz, CD_3_OD) *δ* 153.6 (*C*_Ar_OH), 133.5 (*C*_Ar_), 129.2 (*C*_Ar_), 115.8 (*C*_Ar_), 115.6 (*C*_Ar_), 109.4 (*C*_Ar_Br), 105.3 (O-*C*-OH), 82.6 (O-*C*H), 67.3 (HO-*C*H), 65.8 (HO-*C*H), 59.7 (N*C*H), 57.1 (Ar*C*H_2_), 48.7 (N*C*H_2_), 41.5 (*C*H_2_), 24.1 (*C*H_3_). ESI-HRMS: *m*/*z* calcd for C_15_H_21_O_5_NBr [M + H]^+^: 374.0598; found: 374.0597.

*(2R, 3aR, 6S, 7S, 7aS)-2,6,7-trihydroxy-2-methyl-4-(3-fluoro-4-hydroxylbenzyl)-3a,7a-Dihydrofuro[3,2-b]piperidine* (**30**) (Figures S53 and S54): The synthesized procedure was the same as compound **25**. Colorless syrup, yield 35%, [α]25D−50.0 (*c* 0.08, MeOH). ^1^H NMR (600 MHz, CD_3_OD) *δ* 6.96–6.88 (m, 3H, 2 × *H*_Ar_), 4.03 (s, 1H, O-C*H*), 4.00 (s, 1H, O-C*H*), 3.96 (s, 1H, O-C*H*), 3.90–3.40 (m, 2H, ArC*H*_2_), 2.56 (dd, *J* = 10.7, 4.1 Hz, 1H, NC*H*), 2.48 (d, *J* = 13.5 Hz, 1H, NC*H*_2_), 2.34 (t, *J* = 10.9 Hz, 1H, NC*H*_2_), 2.20–2.14 (m, 1H, C*H*_2_), 2.01 (dd, *J* = 13.5, 3.9 Hz, 1H, C*H*_2_), 1.44 (s, 3H, C*H*_3_). ^13^C NMR (151 MHz, CD_3_OD) *δ* 152.2 (*C*_Ar_F), 150.6 (*C*_Ar_OH), 128.8 (*C*_Ar_), 125.1 (*C*_Ar_), 117.3 (*C*_Ar_), 116.3 (*C*_Ar_), 105.3 (O-*C*-OH), 82.6 (O-*C*H), 67.3 (HO-*C*H), 65.8 (HO-*C*H), 59.7 (N*C*H), 57.4 (Ar*C*H_2_), 48.7 (N*C*H_2_), 41.5 (*C*H_2_), 24.1 (*C*H_3_). ESI-HRMS: *m*/*z* calcd for C_15_H_21_O_5_NF [M + H]^+^: 314.1398; found: 314.1404.

*(2R, 3aR, 6S, 7S, 7aS)-2,6,7-trihydroxy-2-methyl-4-(2-chloro-4-hydroxylbenzyl)-3a,7a-Dihydrofuro[3,2-b]piperidine* (**31**) (Figures S55 and S56): The synthesized procedure was the same as compound **25**. Colorless syrup, yield 35%, [α]25D−37.5 (*c* 0.16, MeOH). ^1^H NMR (600 MHz, CD_3_OD) *δ* 7.23 (d, *J* = 8.3 Hz, 1H, *H*_Ar_), 6.80 (d, *J* = 2.3 Hz, 1H, *H*_Ar_), 6.70 (dd, *J* = 8.4, 2.5 Hz, 1H, *H*_Ar_), 4.06 (t, *J* = 3.9 Hz, 1H, O-C*H*), 3.91 (s, 1H, O-C*H*), 3.80–3.78 (m, 1H, O-C*H*), 3.74 (d, *J* = 13.8, 1H, ArC*H*_2_), 3.26 (d, *J* = 15.5, 1H, ArC*H*_2_), 2.55 (dd, *J* = 11.0, 4.3 Hz, 1H, NC*H*), 2.31 (t, *J* = 10.9 Hz, 1H, NC*H*_2_), 2.19 (d, *J* = 13.6 Hz, 1H, NC*H*_2_), 2.15–2.09 (m, 1H, C*H*_2_), 2.00–1.90 (m, 1H, C*H*_2_), 1.49 (s, 3H, C*H*_3_). ^13^C NMR (151 MHz, CD_3_OD) *δ* 157.2 (*C*_Ar_OH), 134.3 (*C*_Ar_Cl), 131.7 (*C*_Ar_), 126.1 (*C*_Ar_), 115.7 (*C*_Ar_), 113.7 (*C*_Ar_), 107.3 (O-*C*-OH), 81.0 (O-*C*H), 67.9 (HO-*C*H), 66.7 (HO-*C*H), 60.8 (N*C*H), 55.0 (Ar*C*H_2_), 50.1 (N*C*H_2_), 42.6 (*C*H_2_), 22.4 (*C*H_3_). ESI-HRMS: *m*/*z* calcd for C_15_H_21_O_5_NCl [M + H]^+^: 330.1103; found: 330.1104.

*(2R, 3aR, 6S, 7S, 7aS)-2,6,7-trihydroxy-2-methyl-4-(2,6-dichloro-4-hydroxylbenzyl)-3a,7a-Dihydrofuro[3,2-b]piperidine* (**32**) (Figures S57 and S58): The synthesized procedure was the same as compound **25**. Colorless syrup, yield 30%, [α]25D−11.4 (*c* 0.06, MeOH). ^1^H NMR (600 MHz, CD_3_OD) *δ* 6.84 (s, 2H, 2 × *H*_Ar_), 4.10 (d, *J* = 12.7 Hz, 1H, O-C*H*), 4.03 (d, *J* = 3.4 Hz, 1H, O-C*H*), 3.95 (s, 1H, O-C*H*), 3.85–3.65 (m, 2H, ArC*H*_2_), 2.62 (t, *J* = 10.8 Hz, 1H, NC*H*_2_), 2.50 (d, *J* = 6.9 Hz, 1H, NC*H*), 2.43 (dd, *J* = 10.7, 4.2 Hz, 1H, NC*H*_2_), 2.38 (d, *J* = 13.5 Hz, 1H, C*H*_2_), 1.98 (dd, *J* = 13.5, 4.1 Hz, 1H, C*H*_2_), 1.39 (s, 3H, C*H*_3_). ^13^C NMR (151 MHz, CD_3_OD) *δ* 158.1 (*C*_Ar_OH), 136.9 (*C*_Ar_Cl), 136.9 (*C*_Ar_Cl), 122.7 (*C*_Ar_), 115.6 (*C*_Ar_), 115.4 (*C*_Ar_), 104.7 (O-*C*-OH), 82.6 (O-*C*H), 67.3 (HO-*C*H), 66.0 (HO-*C*H), 61.6 (N*C*H), 53.7 (Ar*C*H_2_), 48.7 (N*C*H_2_), 41.3 (*C*H_2_), 24.2 (*C*H_3_). ESI-HRMS: *m*/*z* calcd for C_15_H_20_O_5_NCl_2_ [M + H]^+^: 364.0713; found: 364.0703.

*(2R, 3aR, 6S, 7S, 7aS)-2,6,7-trihydroxy-2-methyl-4-(4-hydroxyl-methoxylbenzyl)-3a,7a-Dihydrofuro[3,2-b]piperidine* (**33**) (Figures S59 and S60): The synthesized procedure was the same as compound **25**. Colorless syrup,yield 46%, [α]25D−30.4 (*c* 0.08, MeOH). ^1^H NMR (600 MHz, CD_3_OD) *δ* 6.81 (d, *J* = 1.7 Hz, 1H, *H*_Ar_), 6.75 (d, *J* = 8.0 Hz, 1H, *H*_Ar_), 6.70 (dd, *J* = 7.9, 1.7 Hz, 1H, *H*_Ar_), 4.06–4.02 (m, 2H, 2 × O-C*H*), 3.97 (s, 1H, O-C*H*), 3.90–3.70 (m, 2H, ArC*H*_2_), 3.83 (s, 3H, OC*H*_3_), 2.59 (dd, *J* = 10.8, 4.0 Hz, 1H, NC*H*), 2.49 (d, *J* = 13.4 Hz, 1H, NC*H*_2_), 2.30 (t, *J* = 10.9 Hz, 1H, NC*H*_2_), 2.16 (d, *J* = 14.1 Hz, 1H, C*H*_2_), 2.03–1.97 (m, 1H, C*H*_2_), 1.45 (s, 3H, C*H*_3_). ^13^C NMR (151 MHz, CD_3_OD) *δ* 147.7 (*C*_Ar_OCH_3_), 145.8 (*C*_Ar_OH), 128.4 (*C*_Ar_), 121.8 (*C*_Ar_), 114.5 (*C*_Ar_), 112.2 (*C*_Ar_), 105.3 (O-*C*-OH), 82.7 (O-*C*H), 67.3 (HO-*C*H), 65.8 (HO-*C*H), 59.8 (N*C*H), 58.2 (Ar*C*H_2_), 55.0 (O*C*H_3_), 48.6 (N*C*H_2_), 41.5 (*C*H_2_), 24.1 (*C*H_3_). ESI-HRMS: *m*/*z* calcd for C_16_H_24_O_6_N [M + H]^+^: 326.1598; found: 326.1605.

*(2R, 3aR, 6S, 7S, 7aS)-2,6,7-trihydroxy-2-methyl-4-(4-hydroxyl-3-ethyoxylbenzyl)-3a,7a-Dihydrofuro[3,2-b]piperidine* (**34**) (Figures S61 and S62): The synthesized procedure was the same as compound **25**. Colorless syrup, yield 49%, [α]25D−18.5 (*c* 0.13, MeOH). ^1^H NMR (600 MHz, CD_3_OD) *δ* 6.80 (d, *J* = 1.7 Hz, 1H, *H*_Ar_), 6.76 (d, *J* = 8.0 Hz, 1H, *H*_Ar_), 6.69 (dd, *J* = 8.0, 1.8 Hz, 1H, *H*_Ar_), 4.12–3.99 (m, 4H, OC*H*_2_, 2 × O-C*H*), 3.94 (s, 1H, O-C*H*), 3.91–3.70 (m, 2H, ArC*H*_2_), 2.59 (dd, *J* = 10.8, 4.0 Hz, 1H, NC*H*), 2.48 (d, *J* = 13.5 Hz, 1H, NC*H*_2_), 2.28 (t, *J* = 10.9 Hz, 1H, NC*H*_2_), 2.16 (d, *J* = 12.9 Hz, 1H, CC*H*_2_), 2.01 (dd, *J* = 13.4, 3.8 Hz, 1H, CC*H*_2_), 1.45 (s, 3H, CC*H*_3_), 1.40 (t, *J* = 7.0 Hz, 3H, CH_2_C*H*_3_). ^13^C NMR (151 MHz, CD_3_OD) *δ* 146.8 (*C*_Ar_OEt), 146.1 (*C*_Ar_OH), 128.4 (*C*_Ar_), 121.8 (*C*_Ar_), 114.6 (*C*_Ar_), 113.7 (*C*_Ar_), 105.3 (O-*C*-OH), 82.6(O-*C*H), 67.3(HO-*C*H), 65.8(HO-*C*H), 64.2 (O*C*H_2_), 59.7 (N*C*H), 58.2 (Ar*C*H_2_), 48.7 (N*C*H_2_), 41.5 (C*C*H_2_), 24.1 (C*C*H_3_), 13.7 (CH_2_*C*H_3_). ESI-HRMS: *m*/*z* calcd for C_17_H_26_O_6_N [M + H]^+^: 340.1755; found: 340.1760.

## 4. Conclusions

In summary, a series of D- and L-arabino-configured Dihydrofuro[3,2-*b*]piperidine derivatives was synthesized as *α*-glucosidase inhibitors. Notably, L-arabino-configured compound **32** exhibited stronger inhibitory potency compared to the positive control, acarbose. The structure–activity relationships showed that the configuration of hydroxyl groups in the *N*-heterocycle, as well as the substituted pattern of the *N*-substituted benzyl group, could have a considerable effect on the inhibitory potency. Drug-likeness prediction demonstrated that compounds **28** and **32** may have the essential properties for the development of drugs. Overall, these newly discovered compounds present a novel chemical scaffold, which could be a choice for the development of α-glucosidase inhibitors.

## Figures and Tables

**Table 1:** The IC_50_ values of the compounds against *α*-glucosidase from yeast *^a^*.

Compound	IC_50_ (μM)	Compound	IC_50_ (μM)
**6**	>20.0	**12**	>20.0
**13**	13.0 ± 0.5	**14**	10.0 ± 0.7
**15**	>20.0	**16**	>20.0
**17**	4.9 ± 0.2	**18**	5.5 ± 0.4
**19**	5.4 ± 0.3	**20**	4.4 ± 0.3
**21**	0.91 ± 0.1	**22**	10.5 ± 0.8
**23**	9.0 ± 0.6	**24**	1.9 ± 0.2
**25**	8.3 ± 0.4	**26**	>20.0
**27**	3.7 ± 0.1	**28**	0.5 ± 0.1
**29**	1.2 ± 0.1	**30**	2.6 ± 0.3
**31**	1.8 ± 0.2	**32**	0.07 ± 0.01
**33**	>20.0	**34**	>20.0
**Acarbose**	2.0 ± 0.2		

*^a^* Values of IC_50_ are the mean ± standard error (SD) of three independent experiments.

**Table 2:** The predicted physicochemical properties of compounds **15**–**36** *.

Compound	MW (g/mol)	RB	HBA	HBD	MR	TPSA (Å^2^)	Mlog*P*
**13**	313.78	2	5	3	82.07	73.16	0.90
**14**, **25**	295.33	2	6	4	79.08	93.39	−0.16
**15**, **26**	295.33	2	6	4	79.08	93.39	−0.16
**16**, **27**	295.33	2	6	4	79.08	93.39	−0.16
**17**, **28**	329.78	2	6	4	84.09	93.39	0.35
**18**, **29**	374.23	2	6	4	86.78	93.39	0.48
**19**, **30**	313.32	2	7	4	79.04	93.39	0.23
**20**, **31**	329.78	2	6	4	84.09	93.39	0.35
**21**, **32**	364.22	2	6	4	89.10	93.39	0.87
**22**, **33**	325.36	3	7	4	85.57	102.62	−0.45
**23**, **34**	339.38	4	7	4	90.38	102.62	−0.20
**24**	453.12	2	6	4	94.48	93.39	1.10

* SwissADME: http://www.swissadme.ch (accessed on 28 February 2024); MW: molecular weight; RB: rotatable bonds; HBA: H-bond acceptor; HBD: H-bond donor; MR: molecular refractivity; TPSA**:** topological polar surface area; Mlog*P*: topological method implemented from Moriguchi I.

## Data Availability

The details of the data supporting the report results in this research are included in the paper and Supplementary Materials.

## Supplementary Materials

The following supporting information can be downloaded at: https://www.mdpi.com/article/10.3390/molecules29051179/s1, Synthesis method and data of compounds **1**–**12**; Figures S1–S62: ^1^H and ^13^C NMR spectra of compounds **2**–**6**, **8**–**14**, and **16**–**34**.
