# Supplementary material for: Synthesis and Biological Evaluation of New Dihydrofuro[3,2-b]piperidine Derivatives as Potent α-Glucosidase Inhibitors
Source: Molecules. 2024 Mar 6;29(5):1179. doi: 10.3390/molecules29051179 (PMC10935252; doi:10.3390/molecules29051179)
Supplement: Supplementary file 1 [file molecules-29-01179-s001.zip › molecules-2809335-supplementary.pdf]

Synthesis and Biological Evaluation of New Dihydrofuro[3,2-b]piperidine Derivatives as Potent α-Glucosidase Inhibitors

Haibo Wang [1,2,3], Xiaojiang Huang [1,2,] Yang Pan [1,2], Guoqing Zhang [1], Senling Tang [1,2], Huawu Shao [1] and Wei Jiao [1,*]

1 Natural Products Research Centre, Chengdu Institute of Biology, Chinese Academy of Sciences,
Chengdu 610041, China; wanghb616@163.com (H.W.); huangxiaojiang22@mails.ucas.ac.cn (X.H.); 18685560668@163.com (Y.P.); guoqingzhanga@outlook.com (G.Z.); tangsl@cib.ac.cn (S.T.);
shaohw@cib.ac.cn (H.S.)
2 University of Chinese Academy of Sciences, Beijing 100049, China
3 Zhejiang Hongyuan Pharmaceutical Co., Ltd., Linhai 317016, China
* Correspondence: jiaowei@cib.ac.cn

Contents:

Synthesis method and of compounds **1-12** 2

^{1}H and ^{13}C NMR spectra of compounds **2-6**, **8-14**, **16-34** 5

Acetyl-2,3,5-tri-*O*-acetyl-*β*-D-arabinofuranoside(1)

Acetyl chloride (2 mL) was added dropwise to the solution of D-arabinose (5g, 33.4 mmol) in methanol at 0 °C. The mixture was stirred for 2.5 h and evaporated the solvent to obtain yellow residue, which was subsequently dissolved in pyridine (30 mL). Then, Ac_2O was added at 0 °C and the reaction was stirred for 8 h. The mixture was poured into cold water (50 mL), and extracted with EtOAc (3×50 mL). The organic layer was then washed with 1N HCl, brine, water and dried over Na_2SO_4, successively. The filtrate was concentrated i*n vacuo*, and the obtained yellow residue was redissolved in Ac_2O (70 mL) and HOAc (20 mL), concentrated sulfuric acid (5 mL) was added dropwise to the solution at 0 °C. After work up and purification, the product was obtained as colorless syrup. Yield 89%. ^{1}H NMR (600MHz, $CDCl_3$) δ 5.37 (t, *J* = 7.1 Hz,1H, O-C*H*), 5.15 (d, *J* = 3.2 Hz, 1H, O-C*H*), 5.02 (t, *J* = 6.5 Hz, 1H, O-C*H*), 4.71 (d, *J* = 3.2 Hz, 1H, O-C*H*), 3.98 (dd, *J* = 12.7, 2.5 Hz, 1H, OCH_2), 3.78 (dd, *J* = 12.6, 4.0 Hz, 1H, OCH_2), 3.43 (s, 3H, COCH_3), 2.12 (s, 3H, COCH_3), 2.11 (s, 3H, COCH_3), 2.04 (s, 3H, COCH_3).

1-*C*-Allyl-2,3,5-tri-*O*-acetyl-*α*-D-arabinofuranoside (2) (Figure S1, S2)

Compound **1** (10g, 31.5 mmol) was dissolved in anhydrous acetonitrile (100 mL), which Allyl-TMS (10mL, 60.3 mmol) was added to. Under argon atmosphere, Lewis acid TMSOTf was added at 0 °C and stirred the reaction for 8 h. Then, the mixture was poured into cold water (50 mL). After work up and purification, the product was obtained as colorless syrup. Yield 62%, Colorless syrup, $[\alpha]_D^{25}$+23.3 (*c* 0.99, $CHCl_3$). ^{1}H NMR (600 MHz, $CDCl_3$) δ 5.75–5.66 (m, 1H, =C*H*), 5.06–4.85 (m, 4H, 2×O-C*H*, =CH_2), 4.20–4.12 (m, 2H, 2×O-C*H*), 4.08 (dd, *J* = 12.6, 5.6 Hz, 1H, OCH_2), 4.01 (dd, *J* = 6.4, 4.8 Hz, 1H, OCH_2), 2.35–2.24 (m, 2H, =CHCH_2), 1.99 (s, 9H, 3 × COCH_3). ^{13}C NMR (151 MHz, $CDCl_3$) δ 170.4(*C*=O), 169.8(*C*=O), 169.7(*C*=O), 133.1(=CH), 117.9(=CH_2), 82.2 (O-CH), 80.4 (O-CH), 80.0 (O-CH), 78.7 (O-CH), 63.2(OCH_2), 36.8(=CHCH_2), 20.7(CH_3), 20.6(CH_3), 20.6(CH_3). ESI-HRMS: *m*/*z* calcd for $C_{14}H_{20}O_7Na$ $[M+Na]^+$: 323.1101; found: 323.1091.

1-*C*-Allyl-2,3-di-*O*-acetyl-5-*O*-mesyl-*α*-D-arabinofuranoside (3)(Figure S3, S4)

A solution of compound **2** treated with 2 mL saturated MeONa in methanol at room temperature for 3h. The mixture was quenched with HOAc and evaporated the solvent to obtain yellow residue, which was subsequently dissolved in pyridine. Then, mesyl chloride (1.2 eq) was added dropwise at -20 °Cand the solution was stirred for 48 h. Finally, Ac_2O was added to this reaction mixture and was stirred at room temperature for 5 h. The mixture was poured into cold water (40 mL), and extracted with EtOAc (3×50 mL). The organic layer was then washed with 1N HCl, brine, water and dried over Na_2SO_4, successively. The filtrate was concentrated i*n vacuo*, and purified by silica gel flash column chromatography (petroleum ether/ethyl acetate, 3:1) to afford compound **3** as a colorless syrup. Yield 61%. $[\alpha]_D^{25}$ +22.2 (*c* 1.0, $CHCl_3$). ^{1}H NMR (600 MHz, $CDCl_3$) δ 5.81–5.72 (m, 1H, =C*H*), 5.14–5.05 (m, 4H, 2×O-C*H*, =CH_2), 4.44 (d, *J* = 11.2 Hz, 1H, CH_2OSO_2), 4.38 (d, *J* = 4.3 Hz, 1H, O-C*H*), 4.18 (d, *J* = 11.6Hz, 1H, CH_2OSO_2), 4.09 (d, *J* = 9.3 Hz, 1H, O-C*H*), 3.05 (s, 3H, OSO_2CH_3), 2.41 (t, *J* = 6.4 Hz, 2H, =C*H*CH_2), 2.10 (s, 3H, COCH_3), 2.08 (s, 3H, COCH_3). ^{13}C NMR (151 MHz, $CDCl_3$) δ 170.0(*C*=O), 169.9(*C*=O), 133.0 (=CH), 118.2(=CH_2), 82.4 (O-CH), 80.6 (O-CH), 79.8 (O-CH), 78.3 (O-CH), 68.4(CH_2OSO_2), 37.7(OSO_2CH_3), 36.6(=CHCH_2), 20.8(COCH_3), 20.7(COCH_3). ESI-HRMS: *m*/*z* calcd for $C_{13}H_{20}O_8SNa$ $[M+Na]^+$: 359.0771; found: 359.0761.

1-*C*-Allyl-2,3-di-*O*-acetyl-5-Azido-5-deoxy-*α*-D-arabinofuranoside (4)(Figure S5, S6)

A solution of **3** (2.7 mmol) in DMF (10 mL) was treated with NaN_3 (0.5 g, 8.2 mmol) at 80°C for 8h. Then, the mixture was poured into cold water (30 mL), and extracted with EtOAc (3×40 mL). The organic layer was successively washed with water (2 × 50 mL), brine (3 × 100 mL), dried over anhydrous Na_2SO_4. The filtrate was concentrated to obtain a colorless syrup, which was subsequently subject to silica gel flash column chromatography (petroleum ether/ethyl acetate, 5:1) to afford compound **4** (0.6 g, 2.1 mmol, 90%) as a colorless syrup. $[\alpha]_D^{25}$ +85.5 (*c* 0.91, $CHCl_3$). ^{1}H NMR (600 MHz, $CDCl_3$) δ 5.83–5.75 (m, 1H, =C*H*), 5.15–5.07 (m, 4H, 2×O-C*H*, =CH_2), 4.13–4.05 (m, 2H, 2×O-C*H*), 3.50 (dd, *J* = 13.0, 3.7 Hz, 1H, N_3CH_2), 3.43 (dd, *J* = 13.0, 5.8 Hz, 1H, N_3CH_2), 2.41 (t, *J* = 6.8 Hz, 2H, =CHCH_2), 2.11 (s, 3H, COCH_3), 2.07 (s, 3H, COCH_3). ^{13}C NMR (151 MHz, $CDCl_3$) δ 170.1 (*C*=O), 170.0(*C*=O), 133.1 (=CH), 118.1 (=CH_2),

82.2 (O-CH), 81.9 (O-CH), 80.2 (O-CH), 79.1 (O-CH), 51.8(N_3CH_2), 36.6 (=CHCH_2), 20.8 (CH_3), 20.7 (CH_3). ESI-HRMS: *m/z* calcd for $C_{12}H_{17}O_5N_3Na$ $[M+Na]^+$: 306.1060; found: 306.1056.

1-*C*-Acetylmethyl-2,3-di-*O*-acetyl-5-Azido-5-deoxy-*α*-D-arabinofuranoside (5) (Figure S7, S8)

To a solution of **4** (1.5 mmol) and $Hg(OAc)_2$ (200 mg, 0.6 mmol) in acetone/water (4:1, 20 mL) was added dropwise a solution of Jones reagent (2 M, 6 mL) at 0 °C. The dark greenish brown mixture was stirred overnight at 0 °C to room temperature and then poured into cold water (40 mL). The aqueous mixture was extracted with EtOAc (3×60 mL). The organic layer was successively washed with water (2×50 mL), brine (5×100 mL), dried over anhydrous Na_2SO_4. The filtrate was concentrated *in vacuo* and the residue was purified by silica gel flash column chromatography (petroleum ether/ethyl acetate, 2:1, R_f = 0.3) to afford compound **5** as a colorless syrup. Yield 98%, $[\alpha]_D^{25}$ +66.5 (*c* 1.3, $CHCl_3$). ^1H NMR (600 MHz, $CDCl_3$) δ 5.05 (dd, *J* = 8.5, 4.7 Hz, 1H, O-C*H*), 5.02 (br s, 1H, O-C*H*), 4.50–4.44 (m, 1H, O-C*H*), 4.10–4.05 (m, 1H, O-C*H*), 3.50–3.37 (m, 2H, N_3CH_2), 2.85–2.70 (m, 2H, $COCH_2$), 2.15 (s, 3H, $COCH_3$), 2.05 (s, 6H, 2 × $COCH_3$). ^{13}C NMR (151 MHz, $CDCl_3$) δ 205.1 (*C*=O), 170.1 (*C*=O), 170.0 (*C*=O), 82.0 (O-CH), 80.5 (O-CH), 78.8 (O-CH), 78.7 (O-CH), 51.6 (N_3CH_2), 46.2 ($COCH_2$), 30.3 (CH_3), 20.7 (CH_3), 20.7 (CH_3). ESI-HRMS: *m/z* calcd for $C_{12}H_{17}O_6N_3Na$ $[M+Na]^+$: 322.1010; found: 322.0995.

1-*C*-Acetylmethyl-5-deoxy-5-amino-*α*-D-arabinopyranoside (6)(Figure S9, S10)

A solution of compound **5** (1 mmol) in 8mL methanol was bubbled with hydrogen in the presence Pd/C catalyst (200 mg) and stirred for 8h at room temperature. Then, the solution was filtered and evaporated to get a yellow syrup, which was immediately treated with 1% MeONa in methanol at room temperature for 8h. The reaction mixture was concentrated without disposal to obtain a yellow crude. Purified the crude with silica gel flash column chromatography (petroleum ether/acetone, 10:1→1:1) to afford the final product **6**. yellowish syrup, Yield 67%. ^1H NMR (600 MHz, MeOD) δ 4.02 (m, 1H, O-C*H*), 3.91 (s, 1H, O-C*H*), 3.80 (s, 1H, O-C*H*), 3.65 (s, 1H, NC*H*), 3.30–3.25 (m, 2H, NCH_2), 3.05–2.93 (m, 2H, $COCH_2$), 2.20 (s, 3H, $COCH_3$). ^{13}C NMR (151 MHz, MeOD) δ207.4 (*C*=O), 69.9 (O-CH), 69.6(O-CH), 63.6 (O-CH), 56.8 (NCH), 49.8 (NCH_2), 44.0 ($COCH_2$), 22.5 (CH_3). ESI-HRMS: *m/z* calcd for $C_8H_{16}O_4N$ $[M+H]^+$: 190.1074; found: 190.1075.

Acetyl-2,3,5-tri-*O*-acetyl-*α*/*β*-L-arabinofuranoside (7)

olorless syrup, Yield 93%, Rf = 0.3 (P.E./ EtOAc, 2:1). ***β***:^1H NMR (600MHz, $CDCl_3$) δ 5.37 (t, *J* = 7.1 Hz, 1H,O-C*H*), 5.15 (d, *J* = 3.2 Hz, 1H, O-C*H*), 5.02 (t, *J* = 6.5 Hz, 1H,O-C*H*), 4.71 (d, *J* = 3.2 Hz, 1H, O-C*H*), 3.98 (dd, *J* = 2.5, 12.7 Hz, 1H, OCH_2), 3.78 (dd, *J* = 4.0, 12.6 Hz, 1H, OCH_2), 3.43 (s, 3H, $COCH_3$), 2.12 (s, 3H, $COCH_3$), 2.11 (s, 3H, $COCH_3$), 2.04 (s, 3H, $COCH_3$).

1-*C*-Allyl-2,3,5-tri-*O*-acetyl-*α*-L-arabinofuranoside (8)(Figure S11, S12)

The synthesized procedure was the same with compound **2**. Colorless syrup, Yield 91%, $[\alpha]_D^{25}$ - 23.6 (*c* 0.40, $CHCl_3$). ^1H NMR (600 MHz, $CDCl_3$) δ 5.83–5.74 (m, 1H, =C*H*), 5.12–5.06 (m, 4H, 2 × O-C*H*, =CH_2), 4.24–4.21 (m, 2H, 2 × O-C*H*), 4.17–4.07 (m, 2H, O-CH_2), 2.42–2.26 (m, 2H, =CHCH_2), 2.14 (s, 9H, $COCH_3$). ^{13}C NMR (151 MHz, $CDCl_3$) δ 170.7 (*C*=O), 170.0 (*C*=O), 169.9 (*C*=O), 133.1 (=CH), 118.0 (=CH_2), 82.3 (O-CH), 80.5 (O-CH), 80.1 (O-CH), 78.7 (O-CH), 63.4 (OCH_2), 36.9 (=CHCH_2), 20.8 (CH_3), 20.8 (CH_3), 20.8 (CH_3). ESI-HRMS: *m/z* calcd for $C_{14}H_{20}O_7Na$ $[M+Na]^+$: 323.1101; found: 323.1105.

1-*C*-Allyl-2,3-di-*O*-acetyl-5-*O*-mesyl-*α*-L-arabinofuranoside (9) (Figure S13, S14)

The synthesized procedure was the same with compound **3**. Colorless syrup, Yield 88%, $[\alpha]_D^{25}$ -23.9 (*c* 0.93, $CHCl_3$). ^1H NMR (600 MHz, $CDCl_3$) δ 5.84–5.72 (m, 1H, =C*H*), 5.23–5.02 (m, 4H, 2 × O-C*H*, =CH_2), 4.41–4.36 (m, 2H, O-C*H*, CH_2OSO_2), 4.19 (d, *J* = 11.6 Hz, 1H, CH_2OSO_2), 4.12–4.05 (m, 1H, O-C*H*), 3.05 (s, 3H, OSO_2CH_3), 2.41 (dd, *J* = 15.8, 9.0 Hz, 2H, =CHCH_2), 2.08(s, 6H, 2×$COCH_3$). ^{13}C NMR (151 MHz, $CDCl_3$) δ 170.1 (*C*=O), 170.0 (*C*=O), 133.0 (=CH), 118.2 (=CH_2), 82.4 (O-CH), 80.6 (O-CH), 79.8 (O-CH), 78.3 (O-CH), 68.4 (CH_2OSO_2), 37.7(OSO_2CH_3), 36.6 (=CHCH_2), 20.8 (CH_3), 20.7 (CH_3). ESI-HRMS: *m/z* calcd for $C_{13}H_{20}O_8SNa$ $[M+Na]^+$: 359.0771; found: 359.0776.

1-*C*-Allyl-2,3-di-*O*-acetyl-5-Azido-5-deoxy-*α*-L-arabinofuranoside (10)(Figure S15, S16)

The synthesized procedure was the same with compound **4**. Colorless syrup, Yield 63%, $[\alpha]_D^{25}$ - 84.2 (*c* 0.51, $CHCl_3$). ^1H NMR (600 MHz, $CDCl_3$) δ 5.85–5.78 (m, 1H, =C*H*), 5.25–5.02 (m, 4H, 2

×O-C*H*, =C*H*$_2$), 4.18–4.08 (m, 2H, 2×O-C*H*), 3.52 (dd, *J* = 13.0, 3.7 Hz, 1H, N$_3$C*H*$_2$), 3.43 (dd, *J* = 13.0, 5.8 Hz, 1H, N$_3$C*H*$_2$), 2.42 (dd, J = 14.2, 7.5 Hz, 2H, =CHC*H*$_2$), 2.08 (s, 6H, 2×COC*H*$_3$). ^{13}C NMR (151 MHz, CDCl$_3$) δ 170.1 (*C*=O), 170.0 (*C*=O), 133.1 (=CH), 118.1 (=CH$_2$), 82.2 (O-CH), 81.9 (O-CH), 80.2 (O-CH), 79.1 (O-CH), 51.8 (N$_3$CH$_2$), 36.6 (=CHCH$_2$), 20.8 (*C*H$_3$), 20.7 (*C*H$_3$). ESI-HRMS: *m/z* calcd for $C_{12}H_{17}O_5N_3Na$ [M+Na]$^+$: 306.1060; found: 306.1059.

1-*C*-Acetylmethyl-2,3-di-*O*-acetyl-5-Azido-5-deoxy-*α*-L-arabinofuranoside (11)(**Figure S17**, **S18**)

The synthesized procedure was the same with compound **5**. Colorless syrup, Yield 98%, $[\alpha]_D^{25}$-66.4 (*c* 0.80, CHCl$_3$). ^{1}H NMR (600 MHz, CDCl$_3$) δ 5.08–5.00 (m, 2H, 2×O-C*H*), 4.53–4.46 (m, 1H, O-C*H*), 4.12–4.07 (m, 1H, O-C*H*), 3.48–3.41 (m, 2H, N$_3$C*H*$_2$), 2.82 (dd, *J* = 16.5, 8.2 Hz, 1H, COC*H*$_2$), 2.75 (dd, *J* = 16.4, 5.1 Hz, 1H, COC*H*$_2$), 2.16 (s, 3H, COC*H*$_3$), 2.07 (s, 6H, 2×COC*H*$_3$). ^{13}C NMR (151 MHz, CDCl$_3$) δ 205.1 (*C*=O), 170.1 (*C*=O), 170.0 (*C*=O), 82.0(O-CH), 80.5(O-CH), 78.8(O-CH), 78.7(O-CH), 51.7 (N$_3$CH$_2$), 46.2 (COCH$_2$), 30.3 (*C*H$_3$), 20.8 (*C*H$_3$), 20.7 (*C*H$_3$). ESI-HRMS: *m/z* calcd for $C_{12}H_{17}O_6N_3Na$ [M+Na]$^+$: 322.1010; found: 322.1022.

1-*C*-Acetylmethyl-5-deoxy-5-amino-*α*-L-arabinopyranoside (12) (**Figure S19**, **S20**)

The synthesized procedure was the same with compound **6**. Colorless syrup, Yield 65%. ^{1}H NMR (600 MHz, MeOD) δ 4.00 (d, *J* = 4.8 Hz, 1H, O-C*H*), 3.94–3.86 (m, 1H, O-C*H*), 3.78 (d, *J* = 4.1 Hz, 1H, O-C*H*), 3.64 (s, 1H, NC*H*), 3.50–3.35 (m, 1H, NC*H*$_2$), 3.02–2.91 (m, 2H, NC*H*$_2$, COC*H*$_2$), 2.82 (br dd, *J* = 11.6 Hz, 1H, COC*H*$_2$), 2.18 (s, 3H, COC*H*$_3$). ^{13}C NMR (151 MHz, MeOD) δ 207.4 (*C*=O), 74.7 (O-CH), 71.2(O-CH), 68.7(O-CH), 63.5 (NCH), 49.8 (NCH$_2$), 44.0 (*C*H$_2$), 22.5 (*C*H$_3$). ESI-HRMS: *m/z* calcd for $C_8H_{16}O_4N$ [M+H]$^+$: 190.1074; found: 190.1081.

1. ^{1}H NMR and ^{13}C NMR spectra of intermediate compounds.

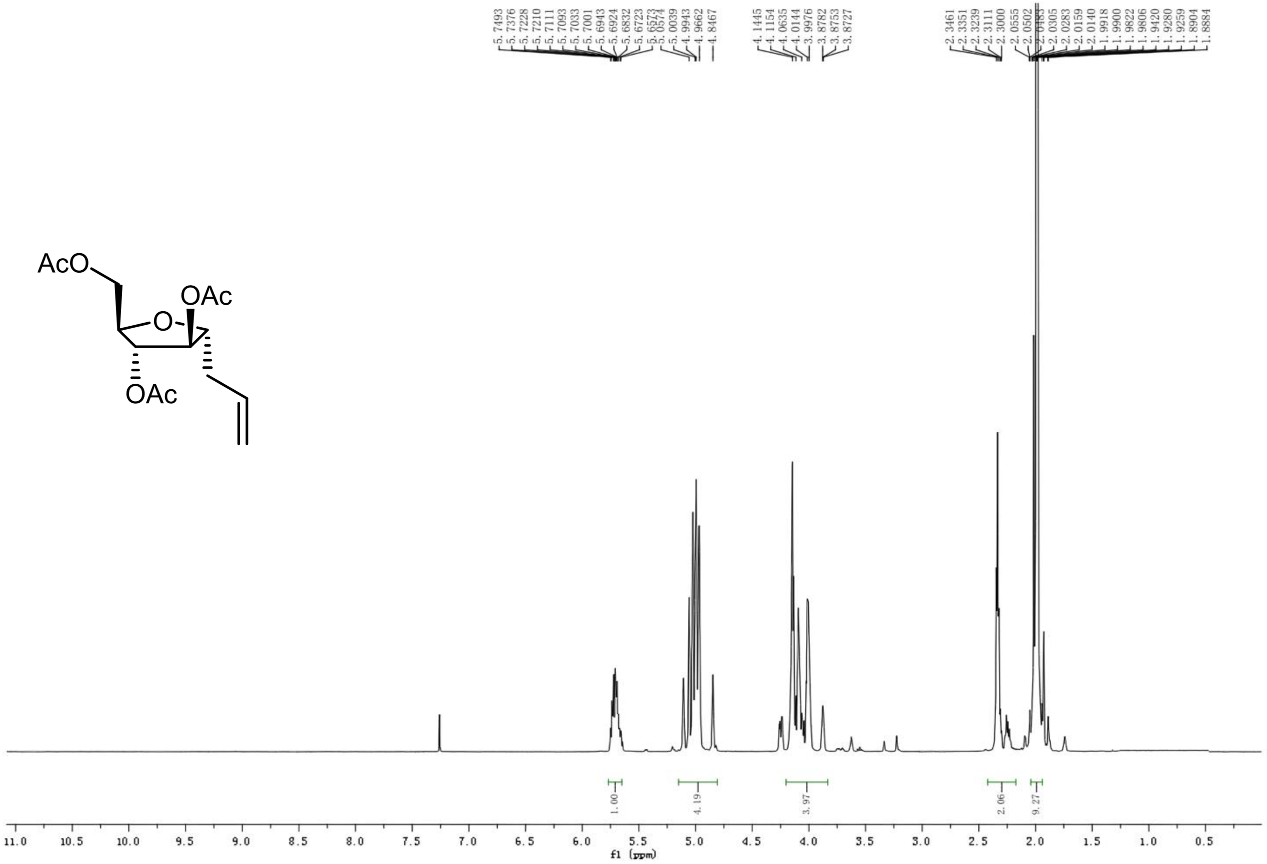

Figure S1. The ^{1}H NMR of compound **2**.

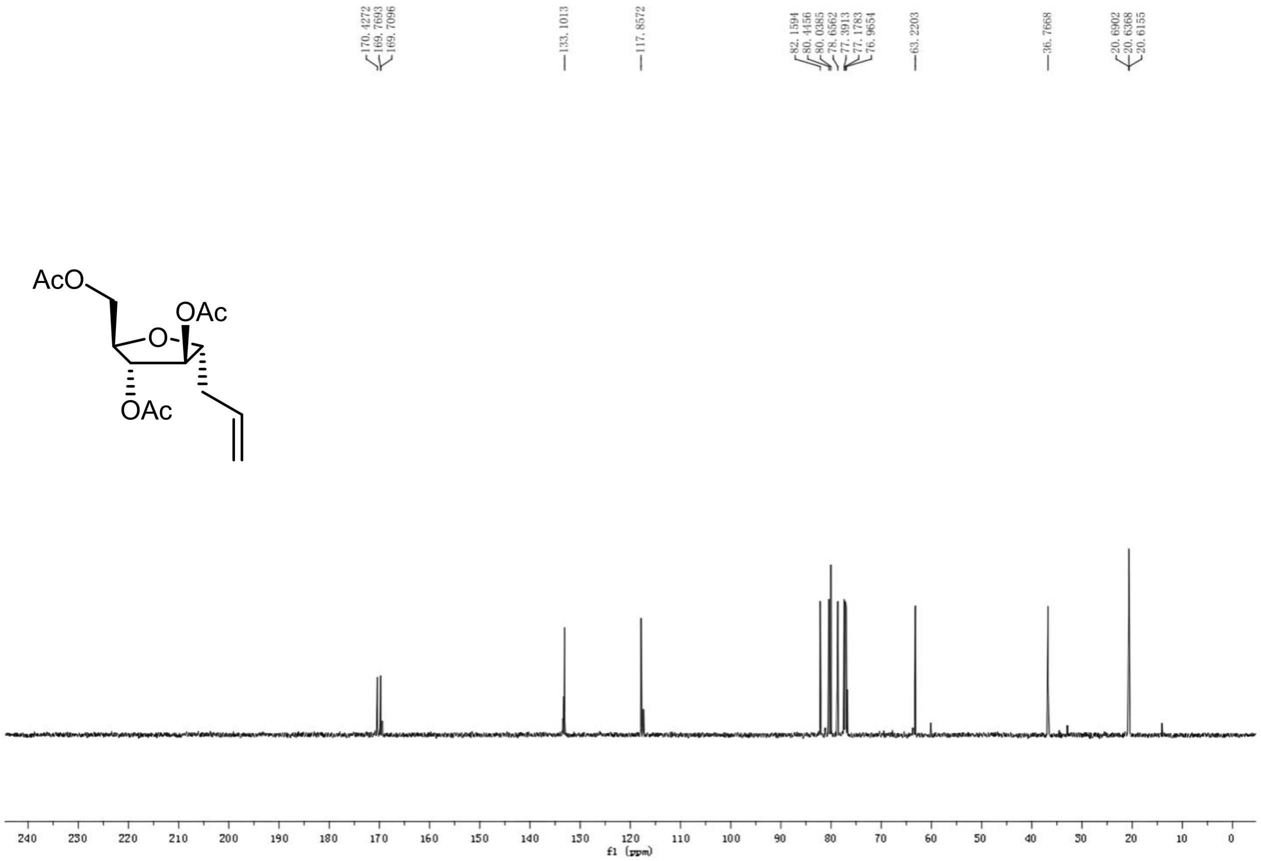

Figure S2. The ^{13}C NMR of Compound **2**.

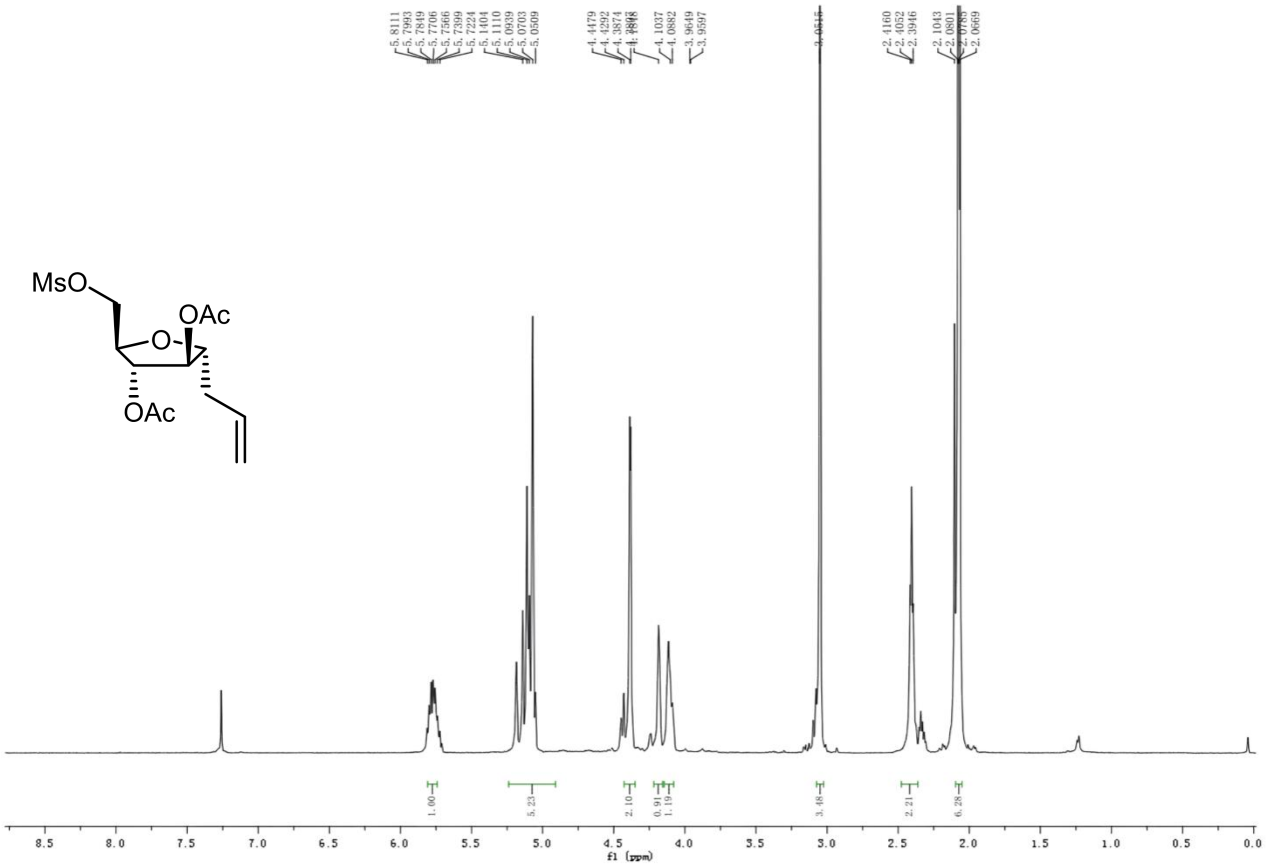

Figure S3. The ^{1}H NMR of compound **3**.

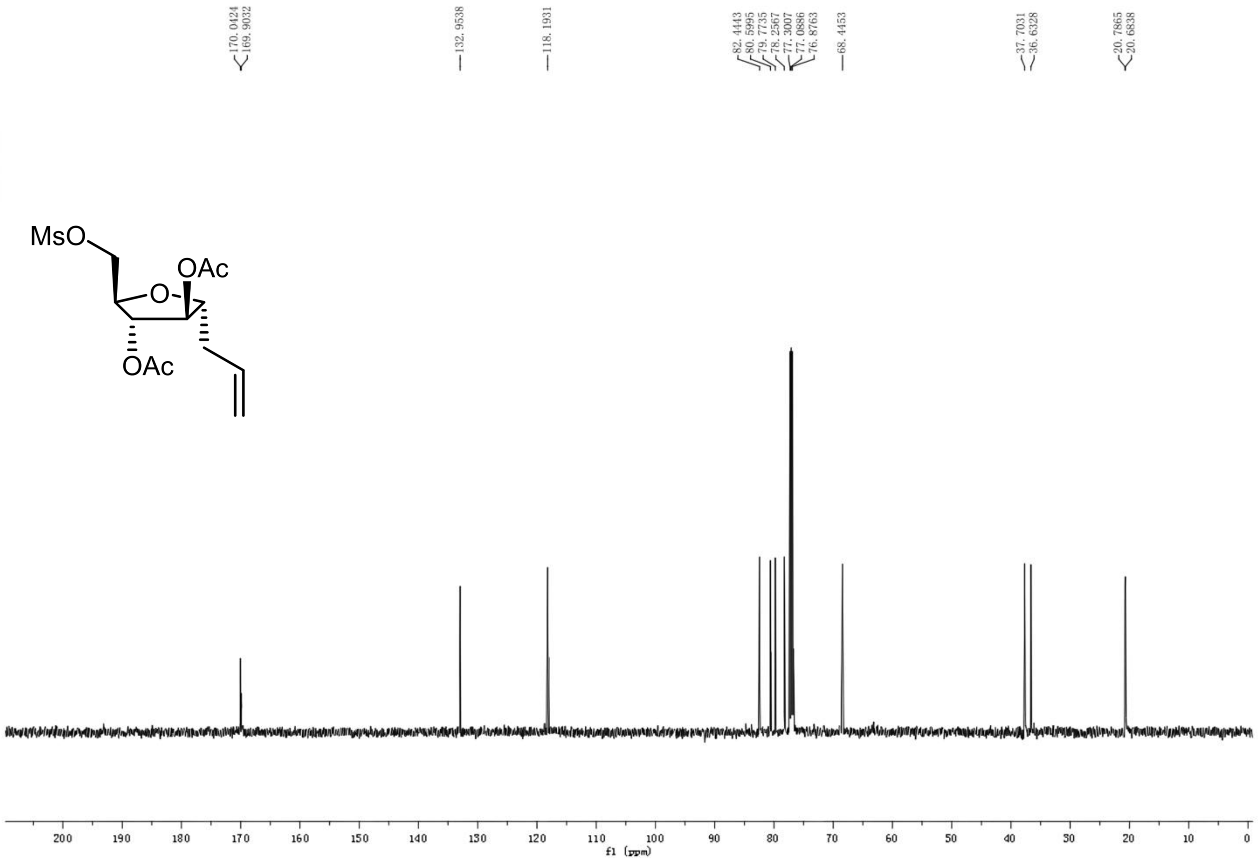

Figure S4. The ^{13}C NMR of Compound **3**.

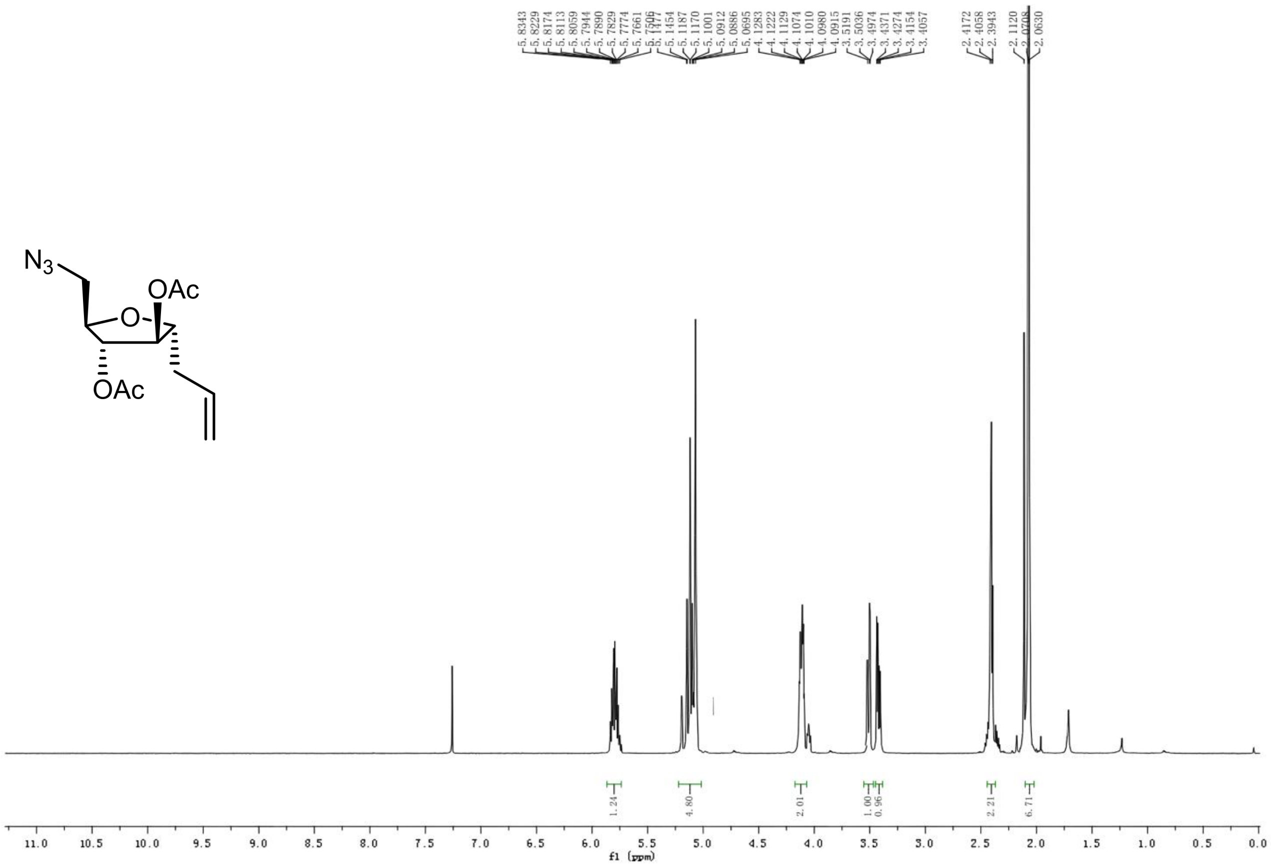

Figure S5. The ^{1}H NMR of compound **4**.

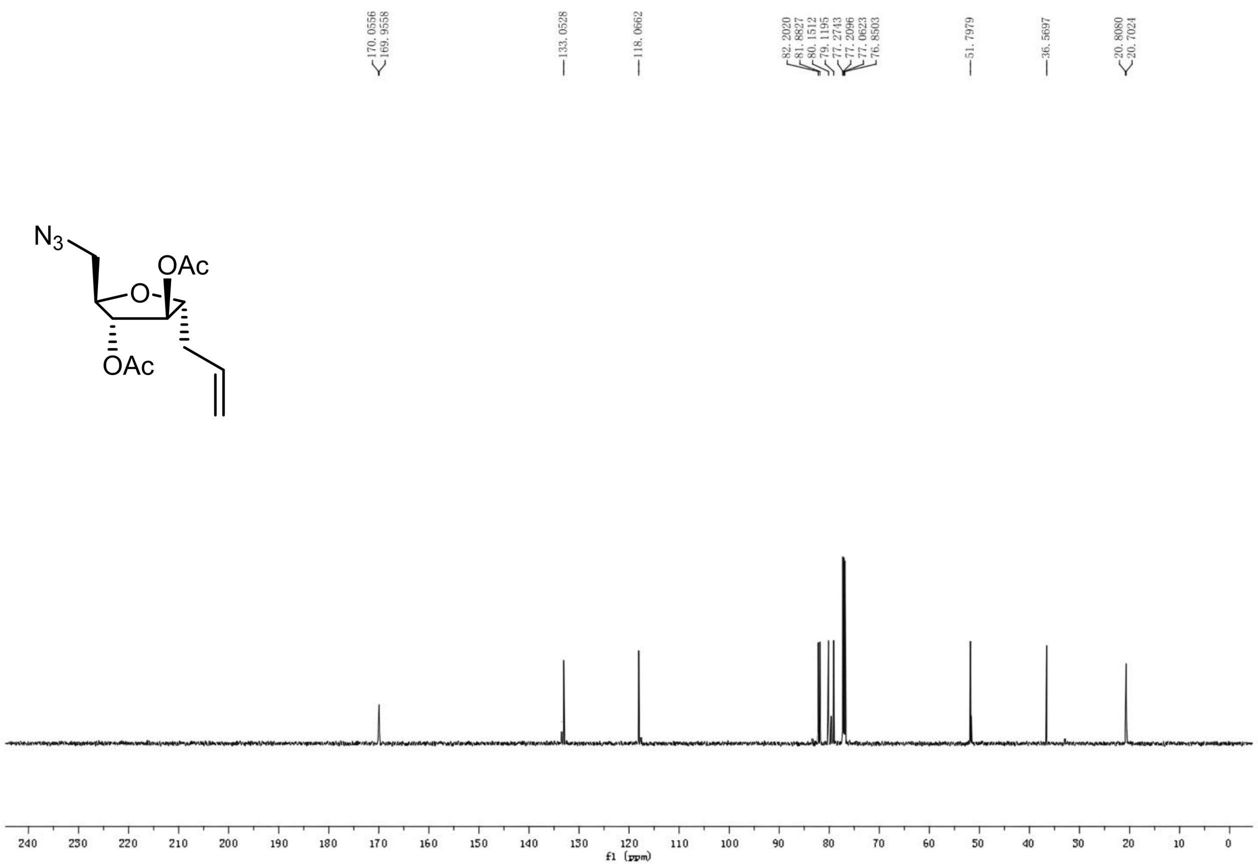

Figure S6. The ^{13}C NMR of Compound **4**.

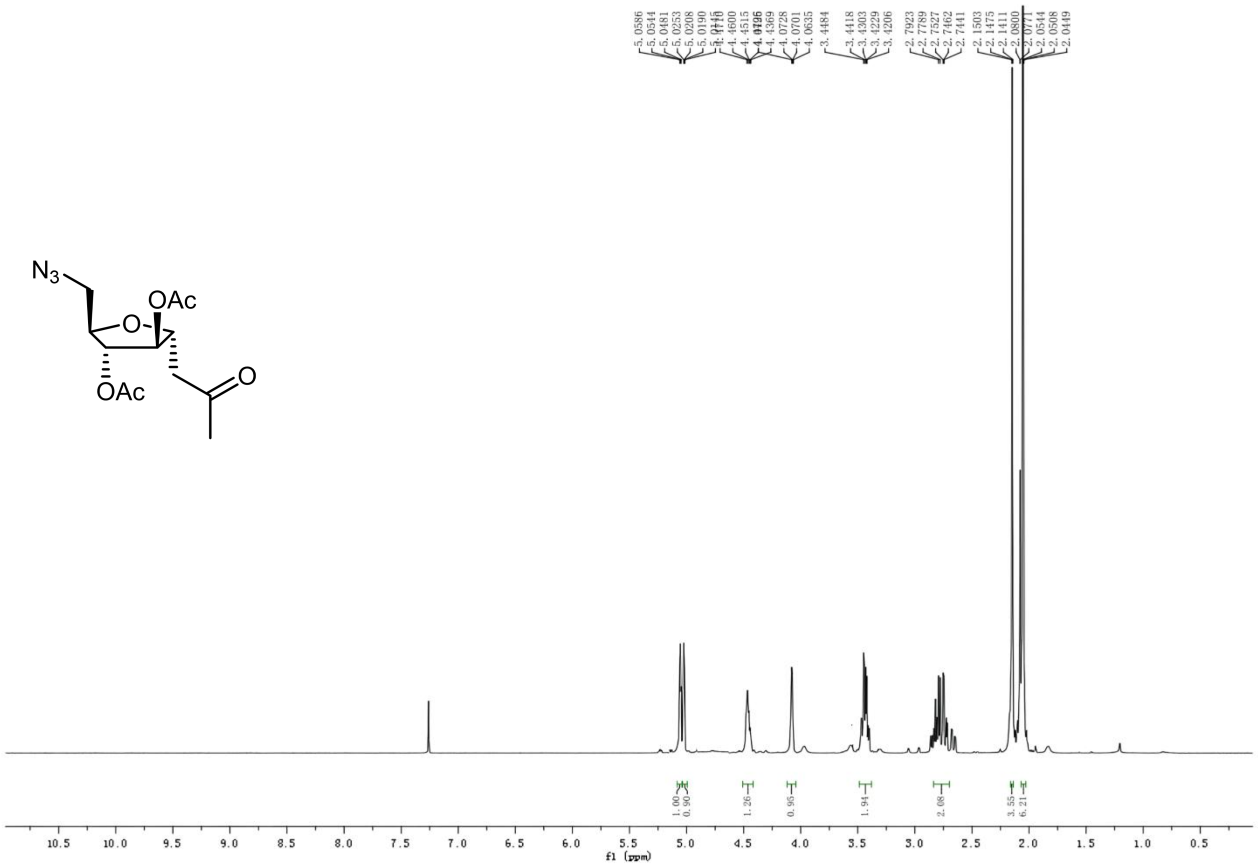

Figure S7. The ^{1}H NMR of compound **5**.

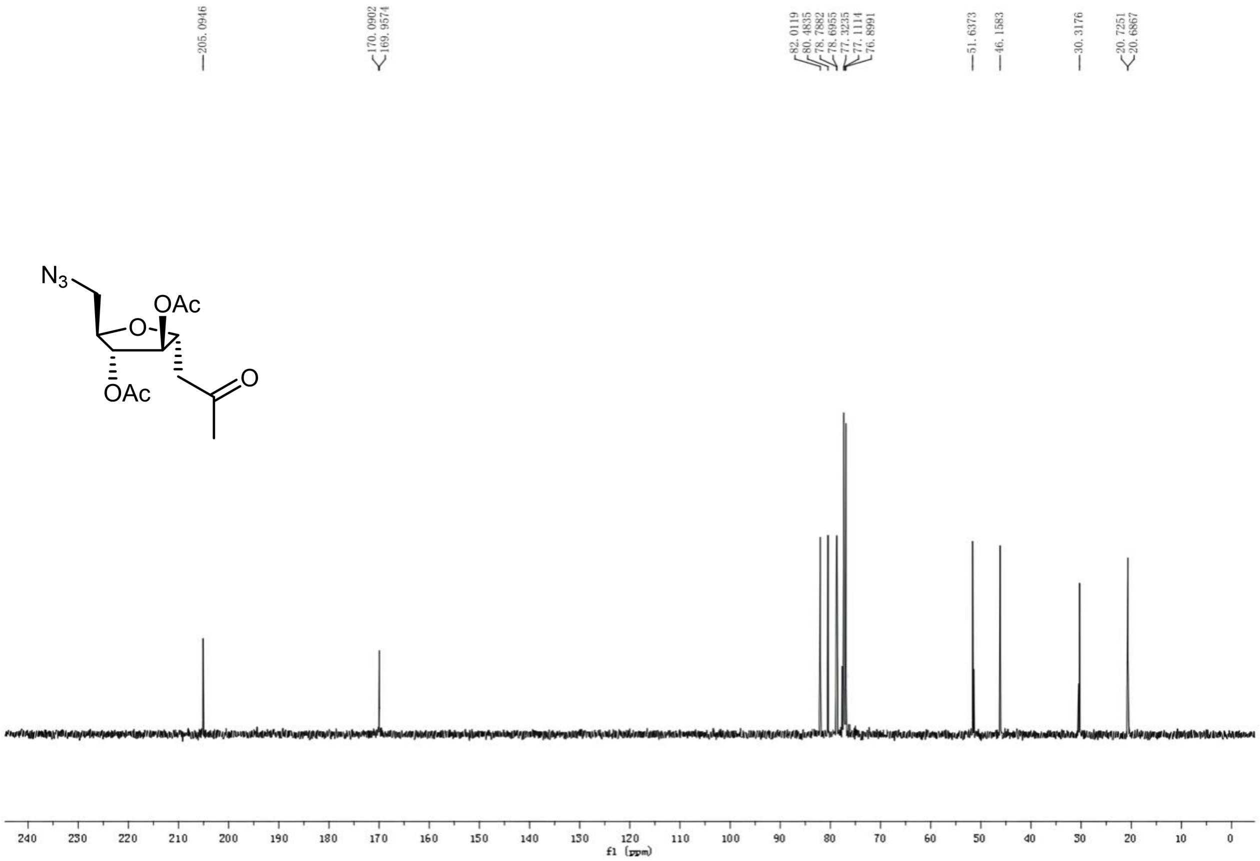

Figure S8. The ^{13}C NMR of Compound **5**.

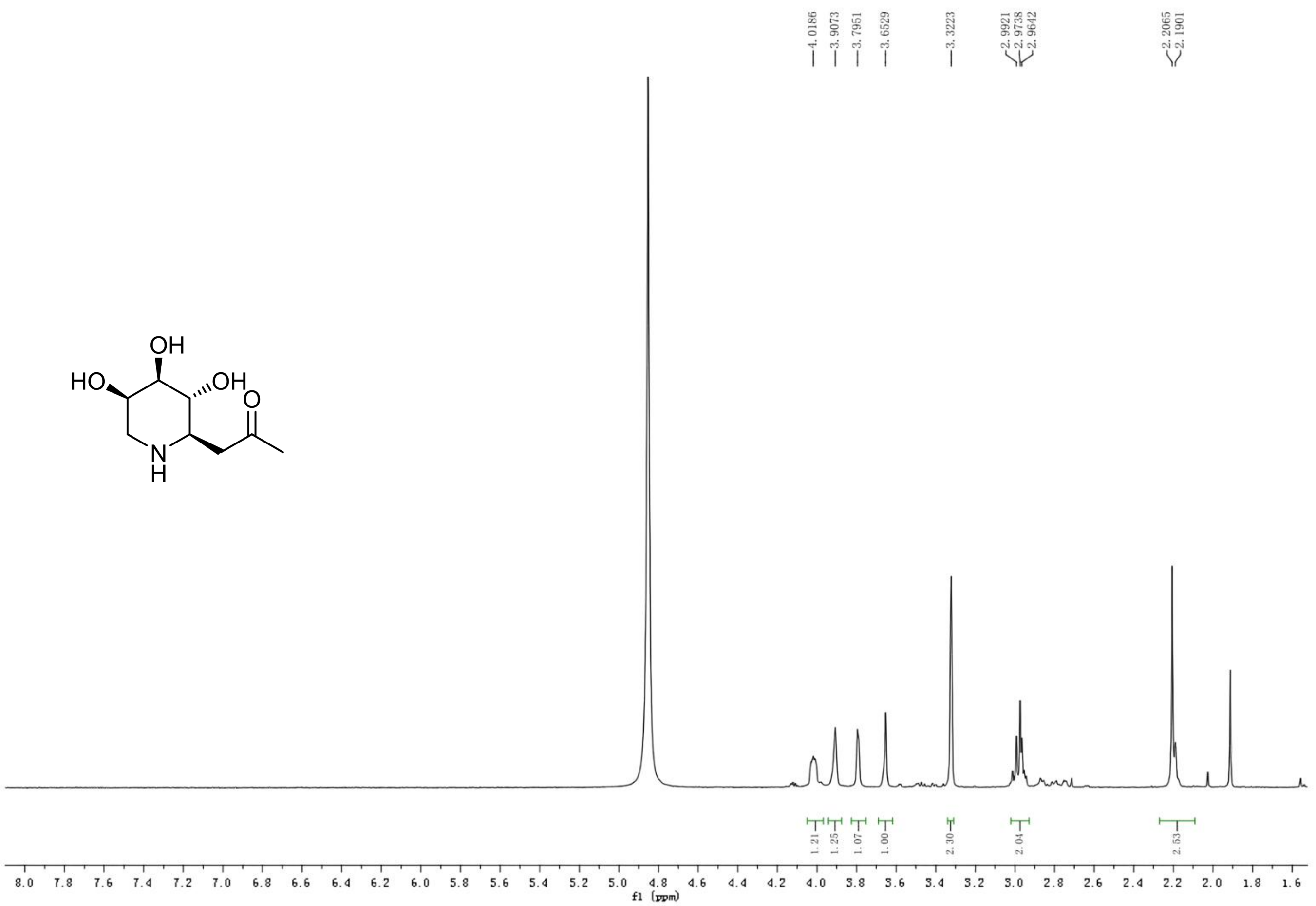

Figure S9. The ^{1}H NMR of compound **6**.

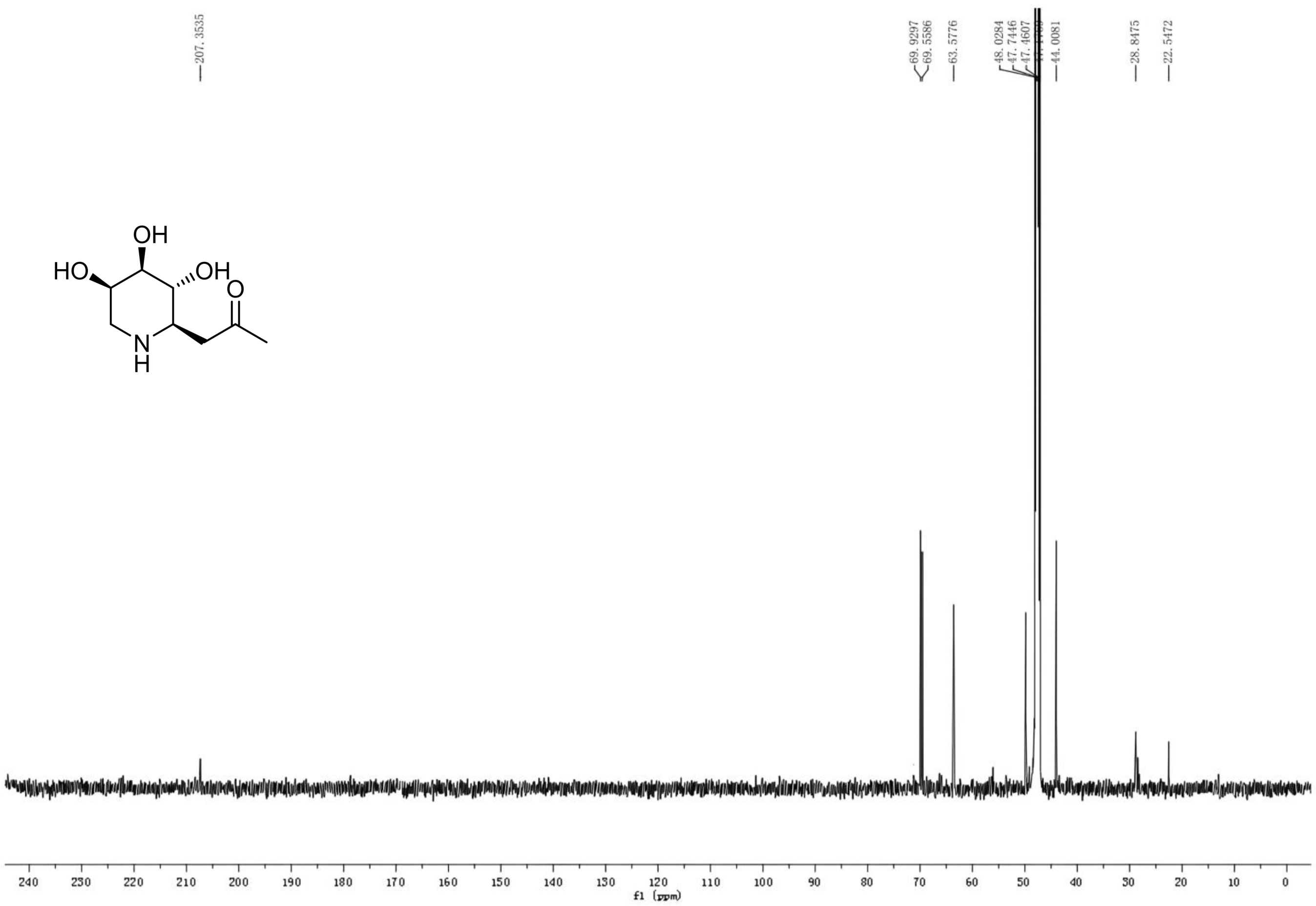

Figure S10. The ^{13}C-NMR of Compound **6**.

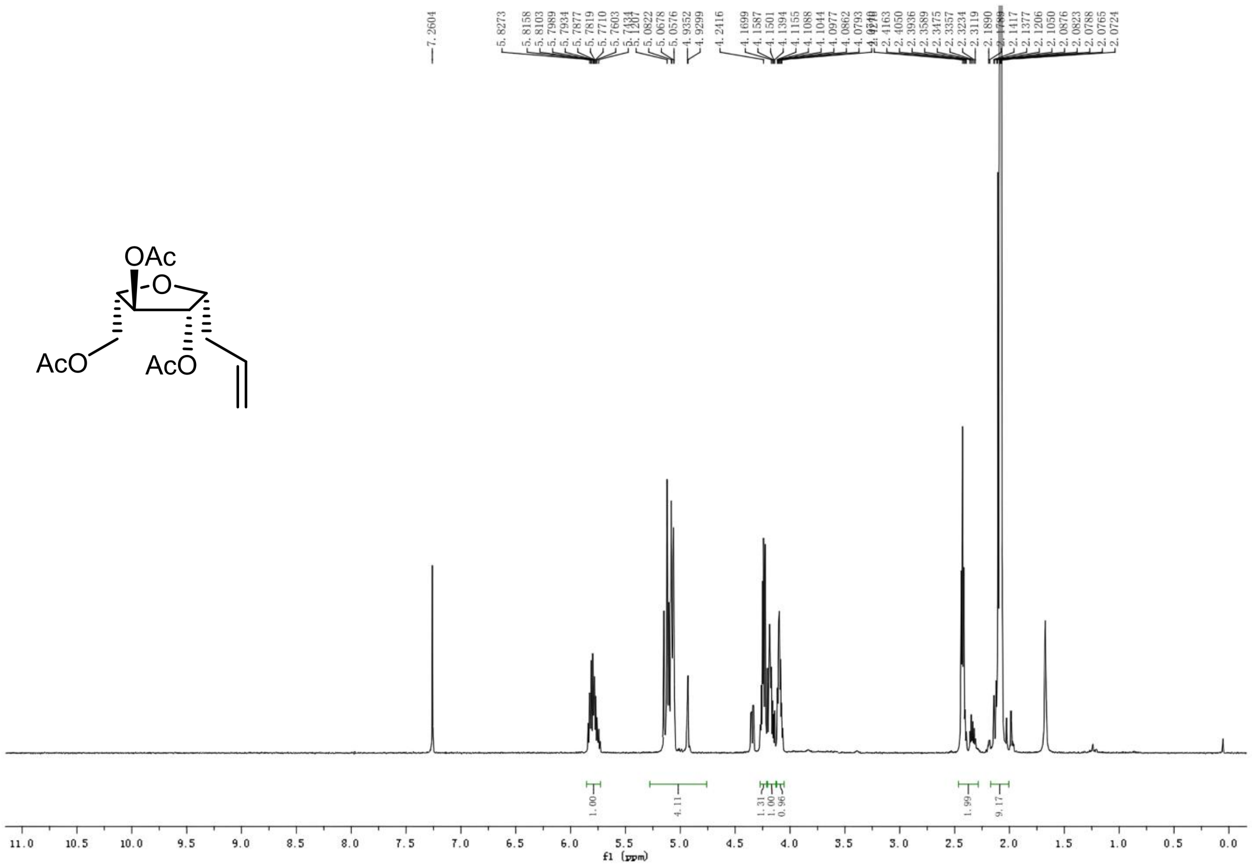

Figure S11. The ^{1}H NMR of compound **8**.

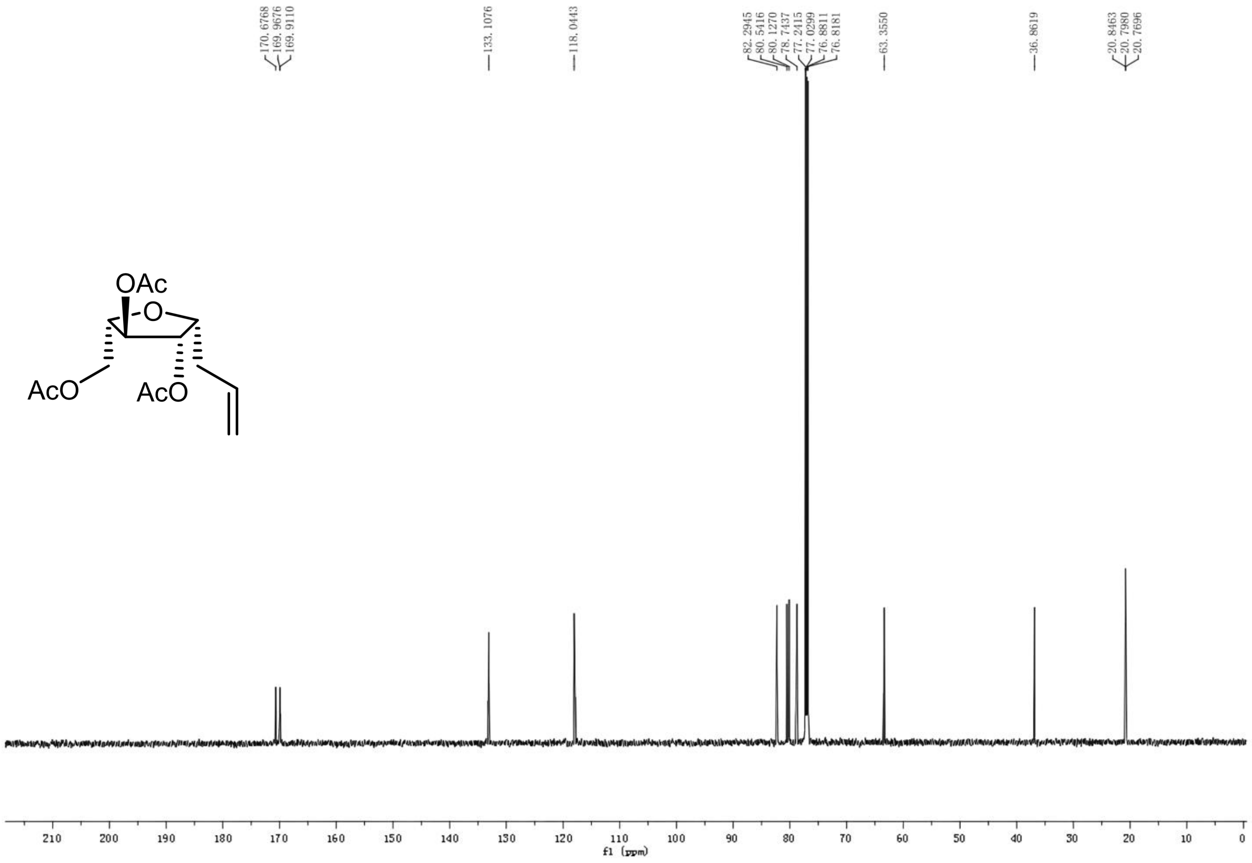

Figure S12. The ^{13}C NMR of Compound **8**.

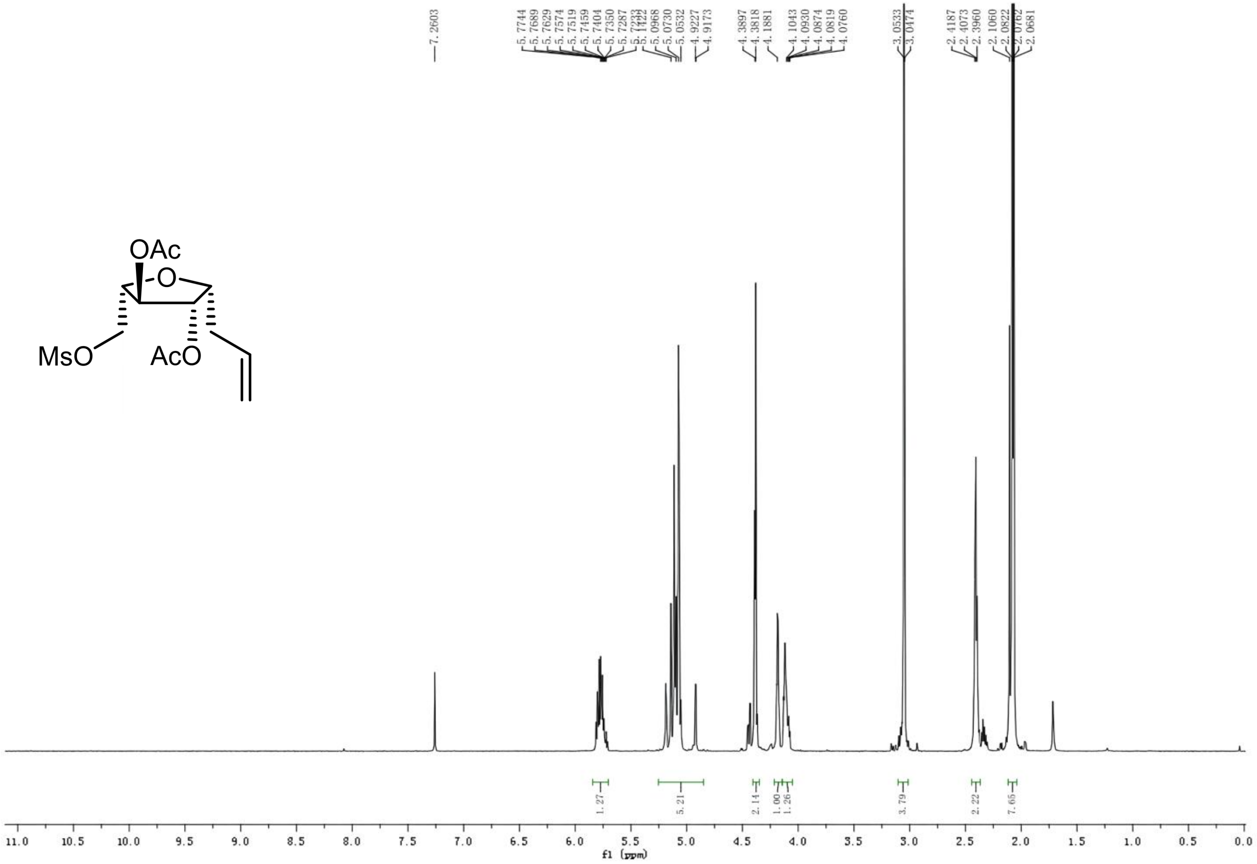

Figure S13. The ^{1}H NMR of compound **9**.

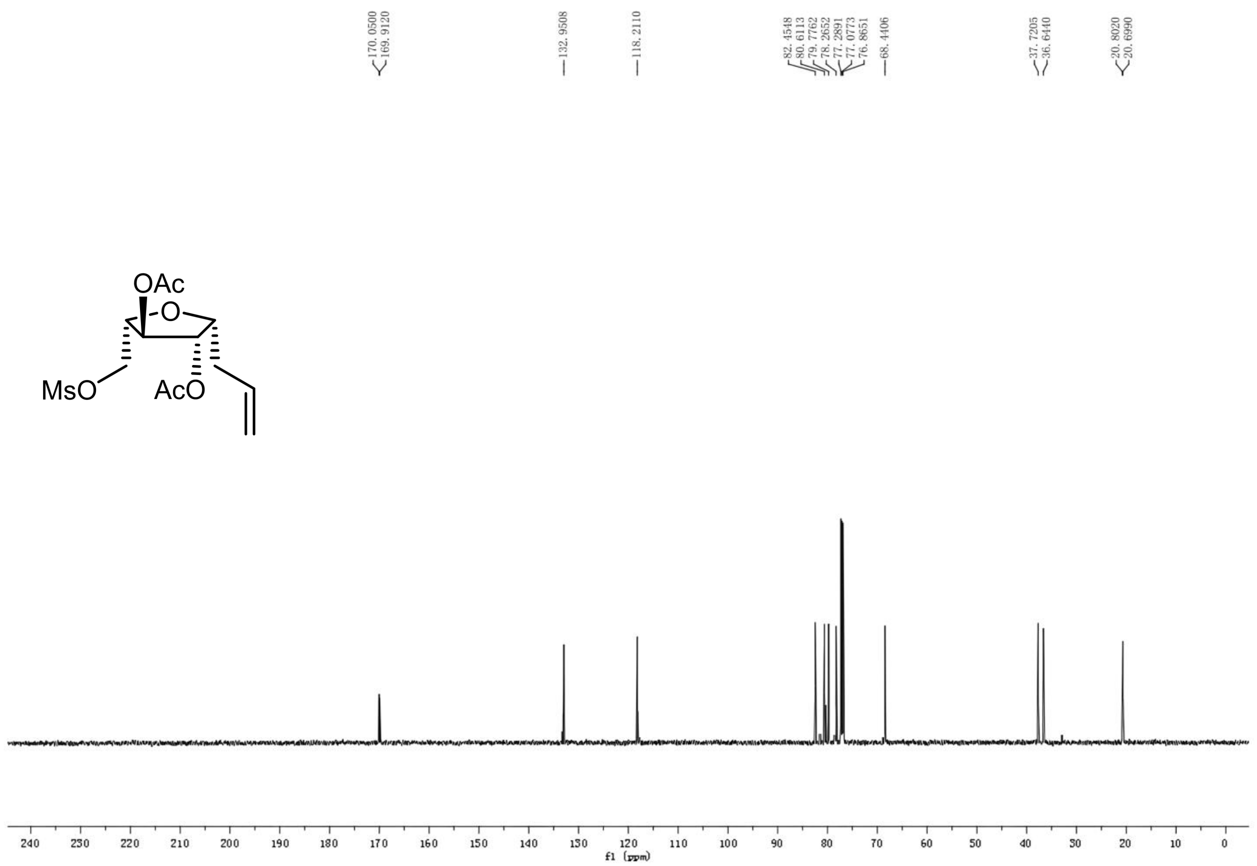

Figure S14. The ^{13}C NMR of Compound **9**.

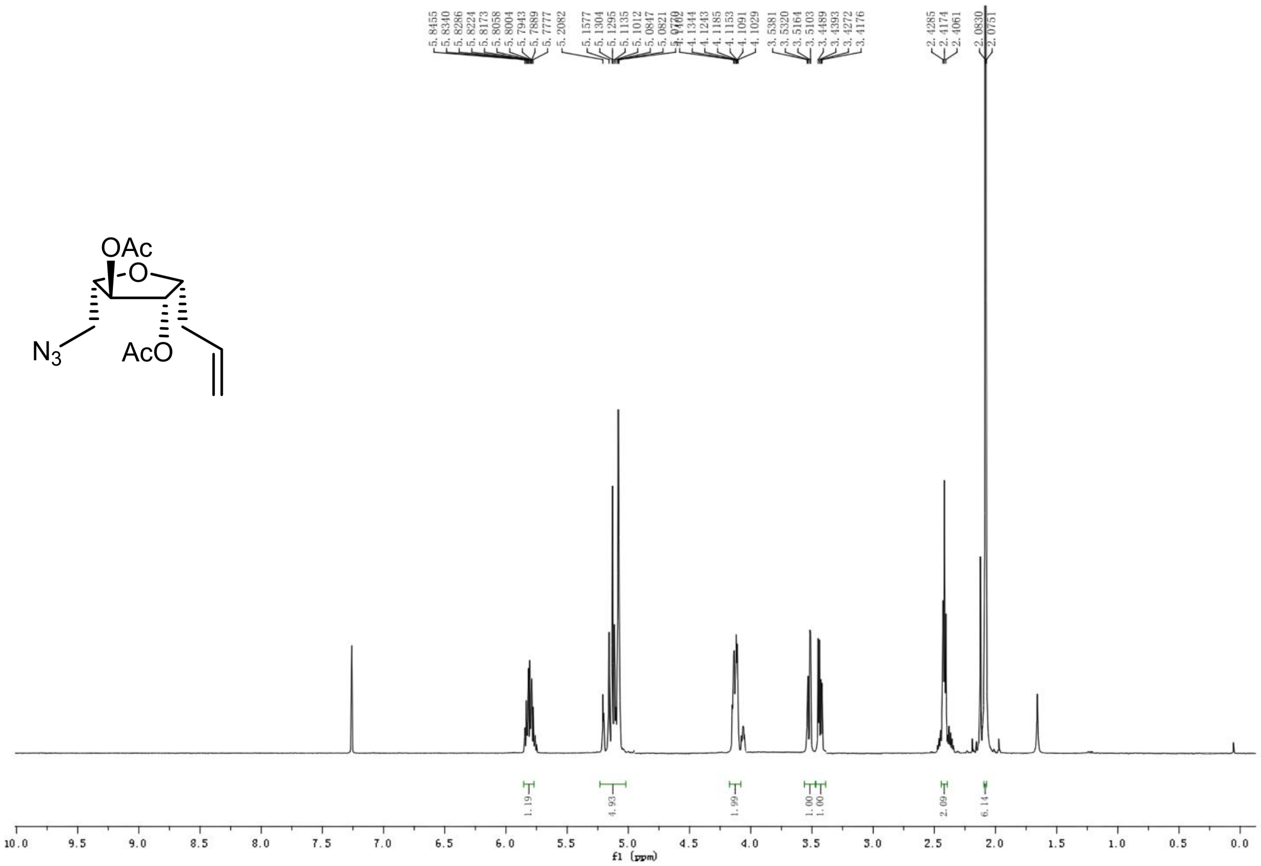

Figure S15. The ^{1}H NMR of compound **10**.

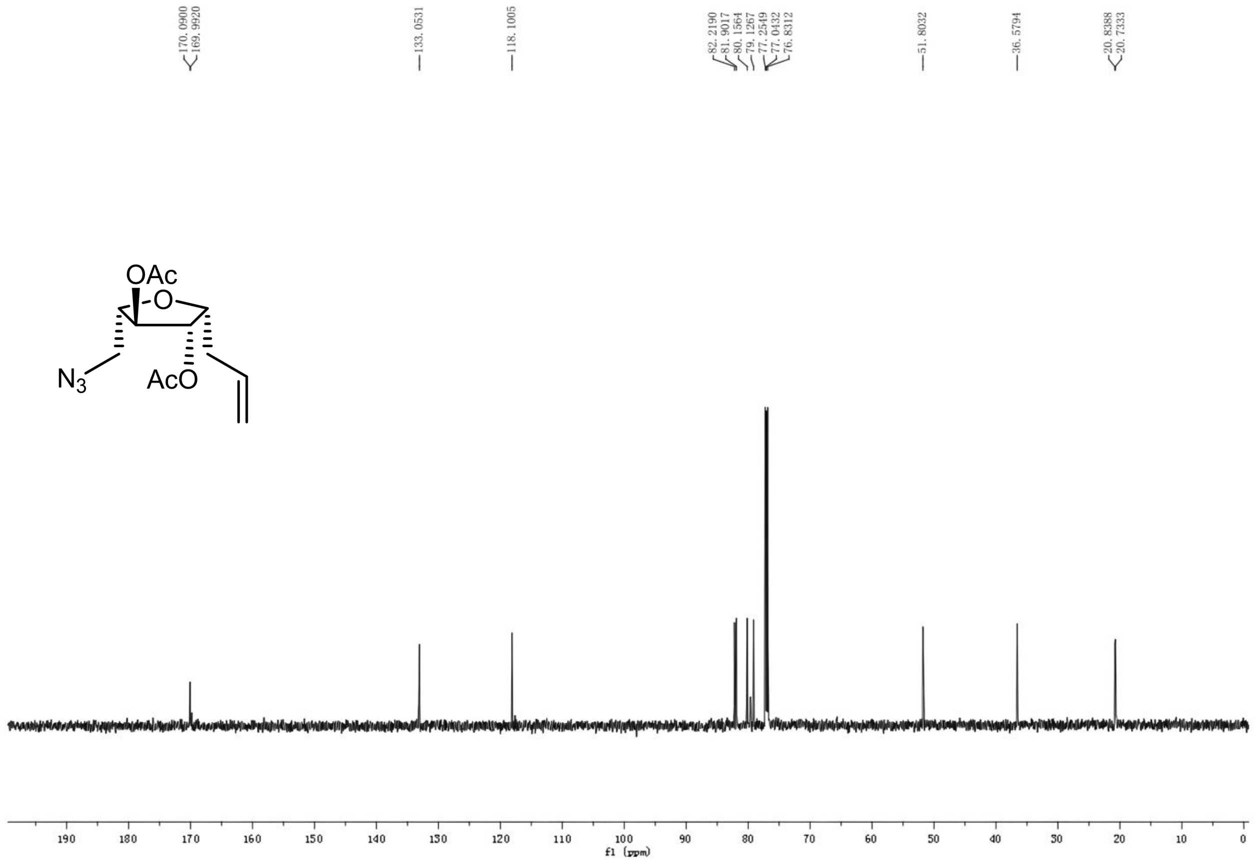

Figure S16. The ^{13}C NMR of Compound **10**.

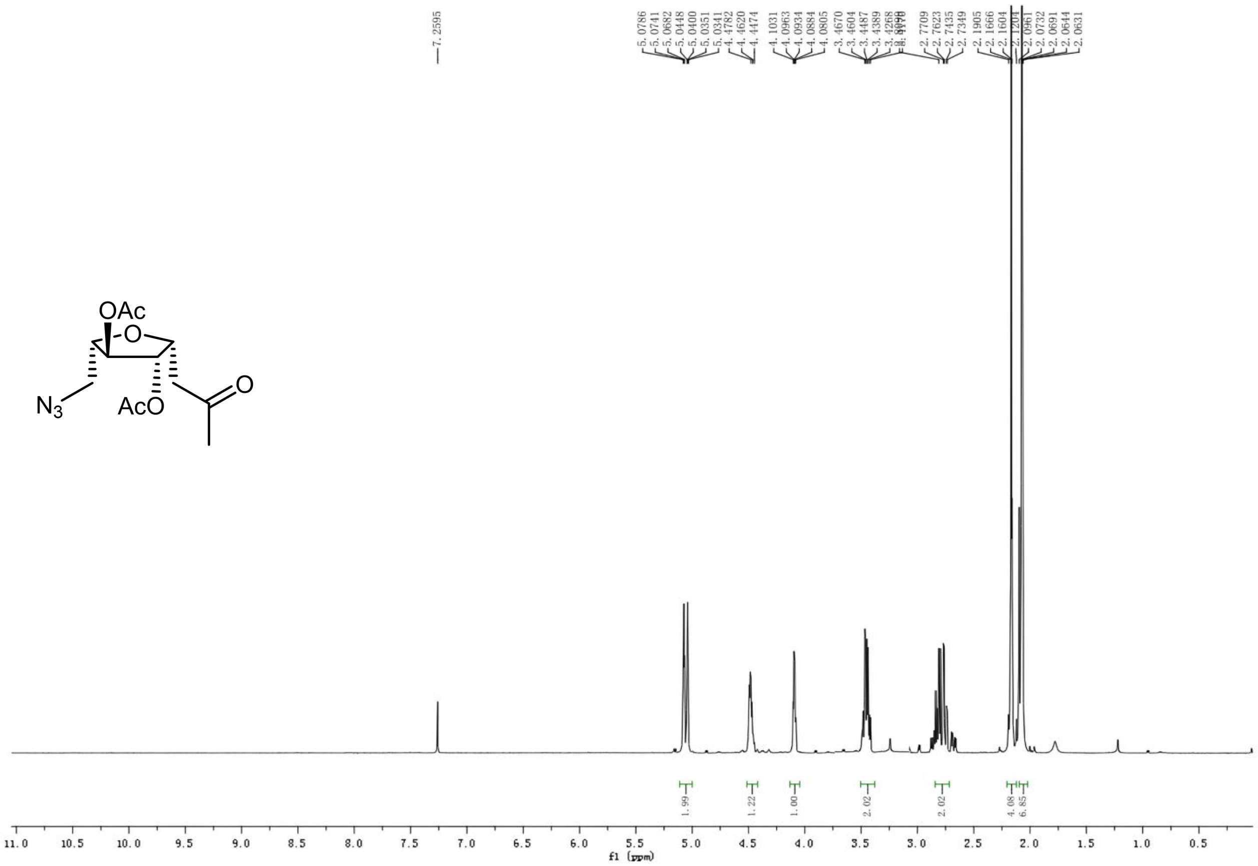

Figure S17. The ^{1}H NMR of compound **11**.

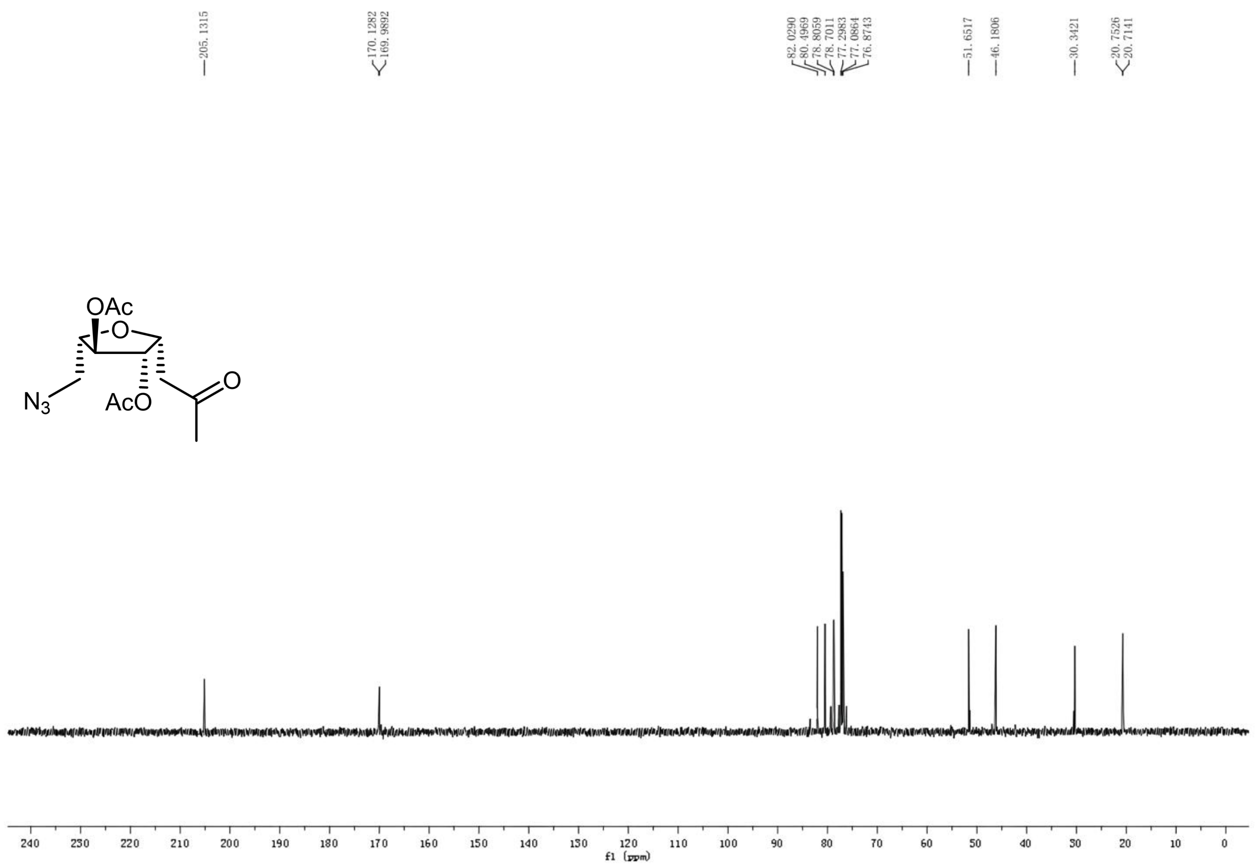

Figure S18. The ^{13}C NMR of Compound **11**.

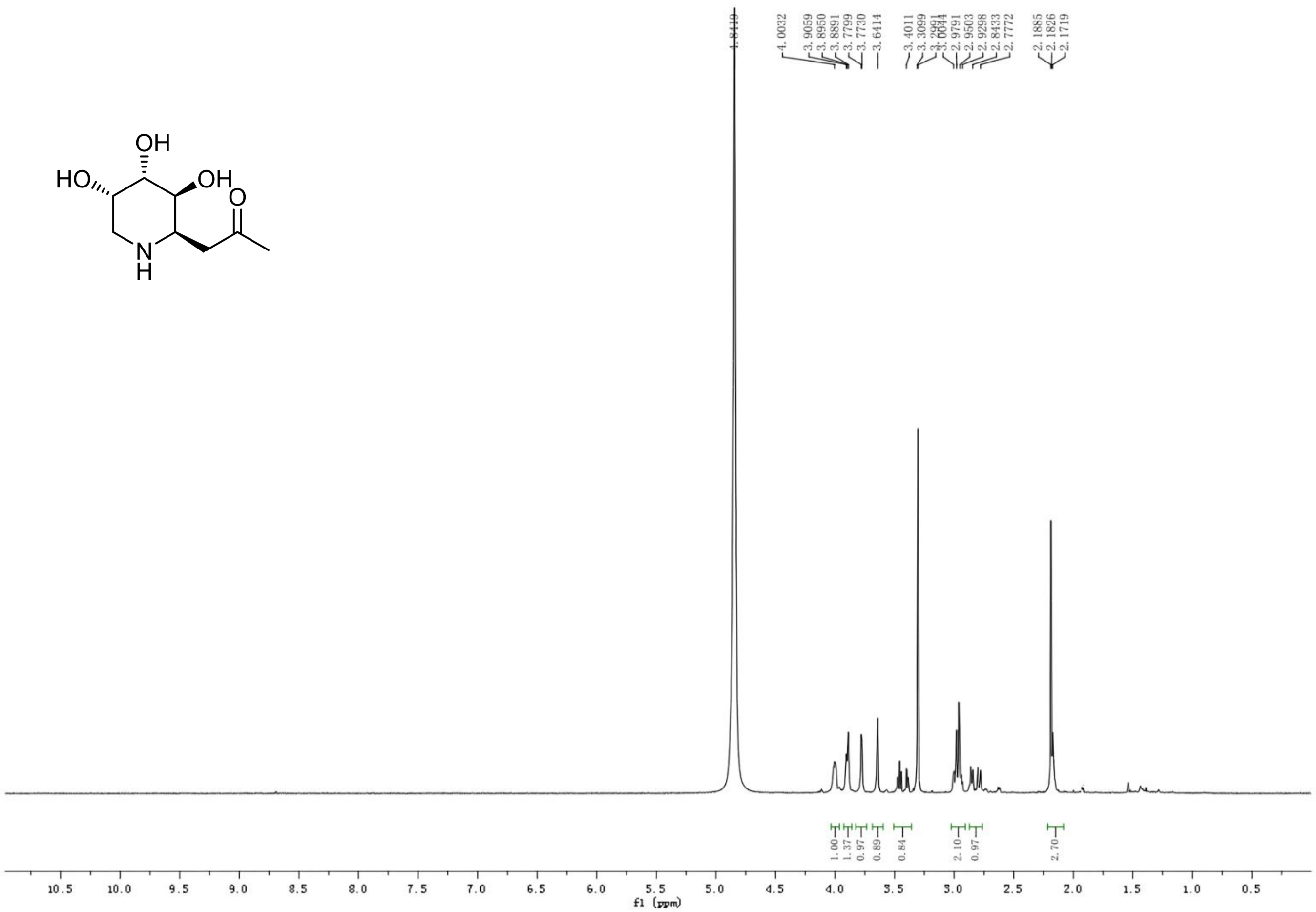

Figure S19. The ^{1}H NMR of compound **12**.

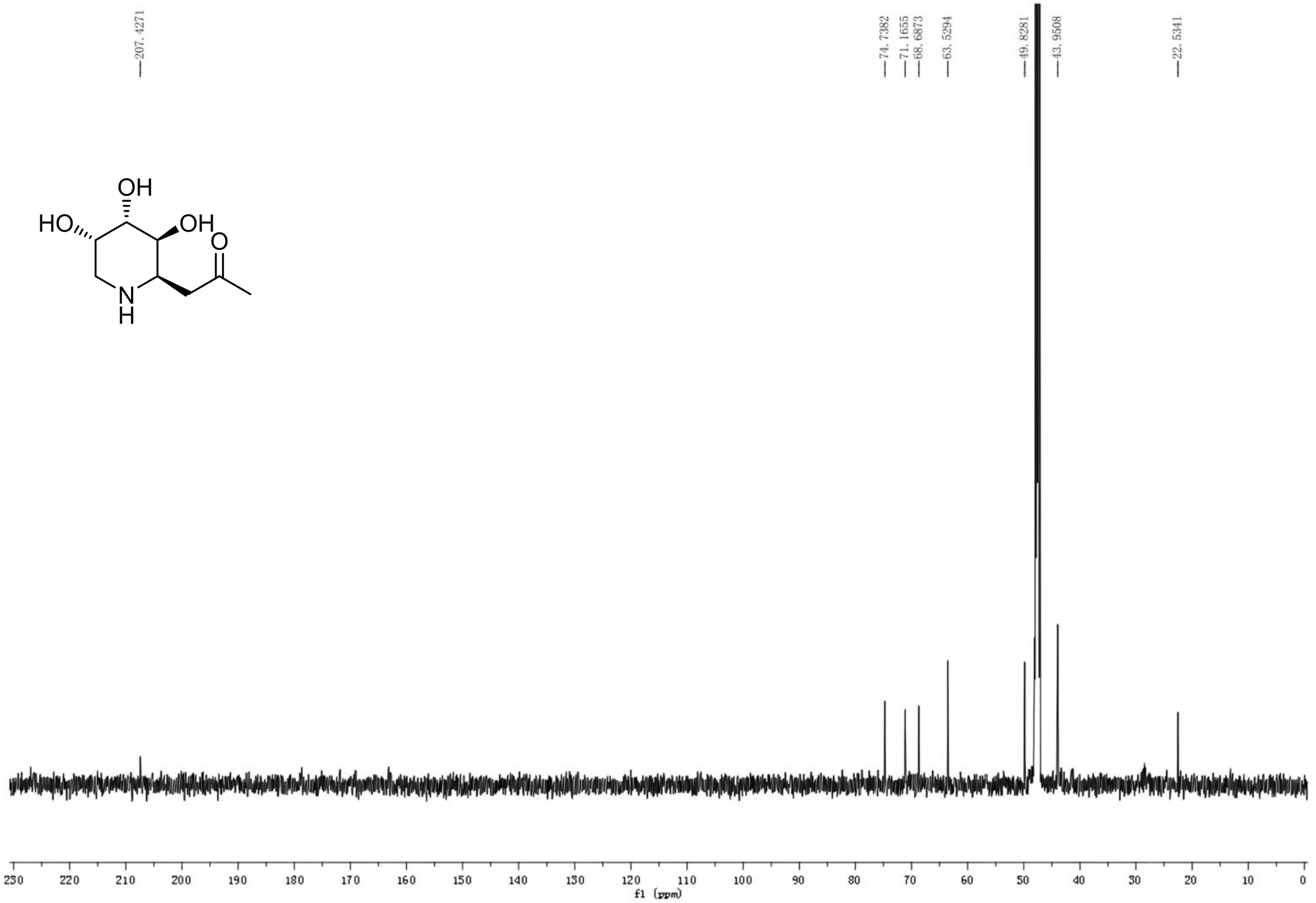

Figure S20. The ^{13}C-NMR of Compound **12**.

2. ^{1}H NMR and ^{13}C NMR spectra of dihydrofuro[3,2-b]piperidine.

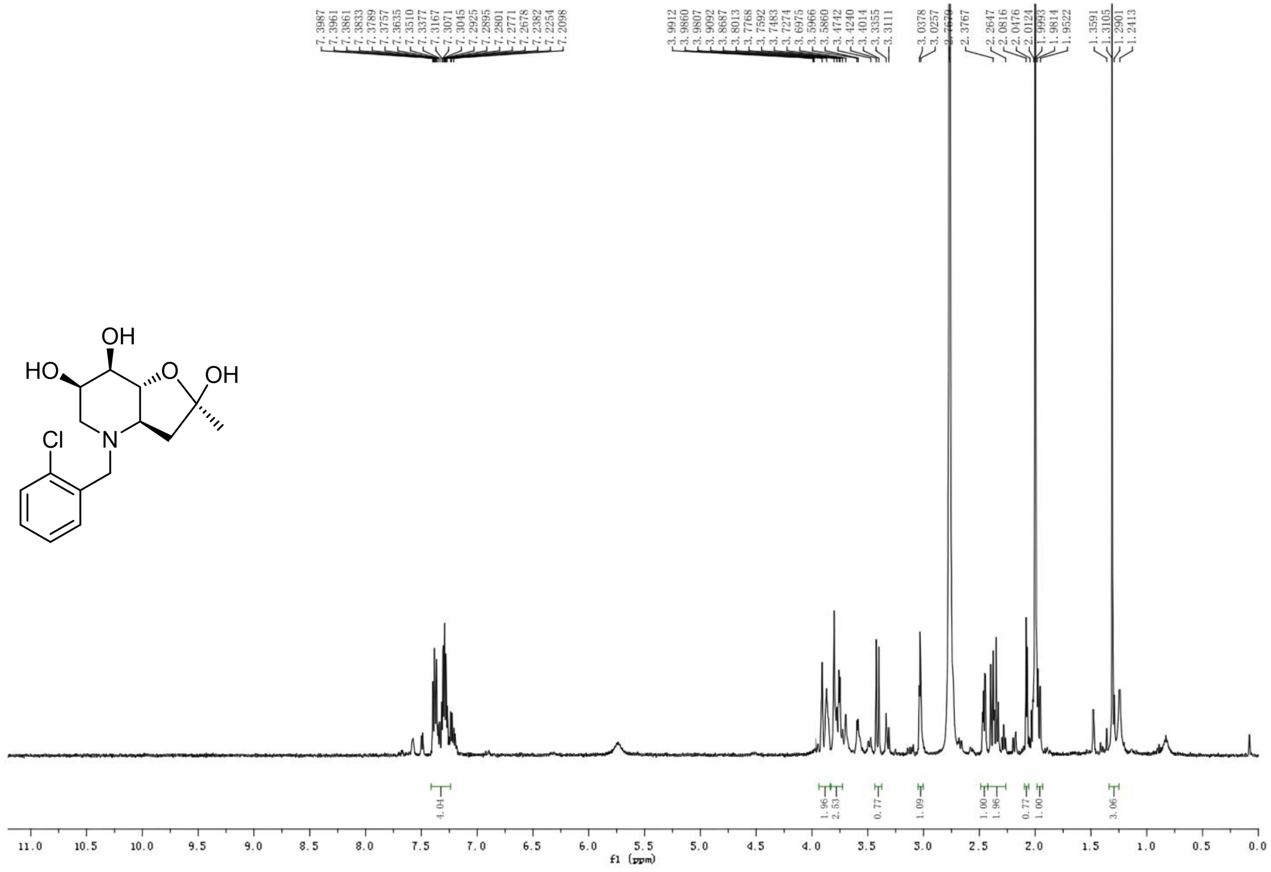

Figure S21. The ^{1}H NMR of compound **13**.

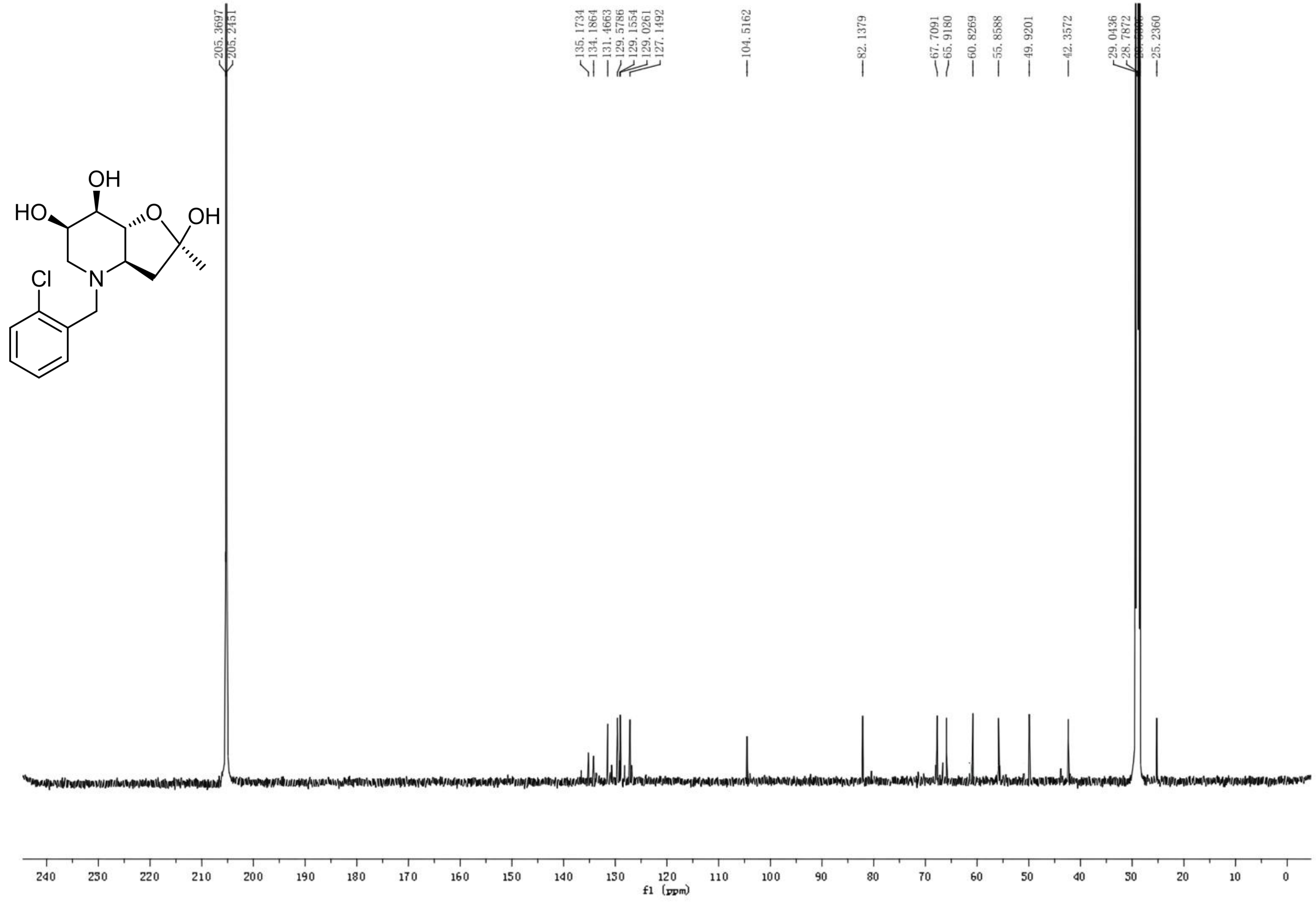

Figure S22. The ^{13}C-NMR of Compound **13**.

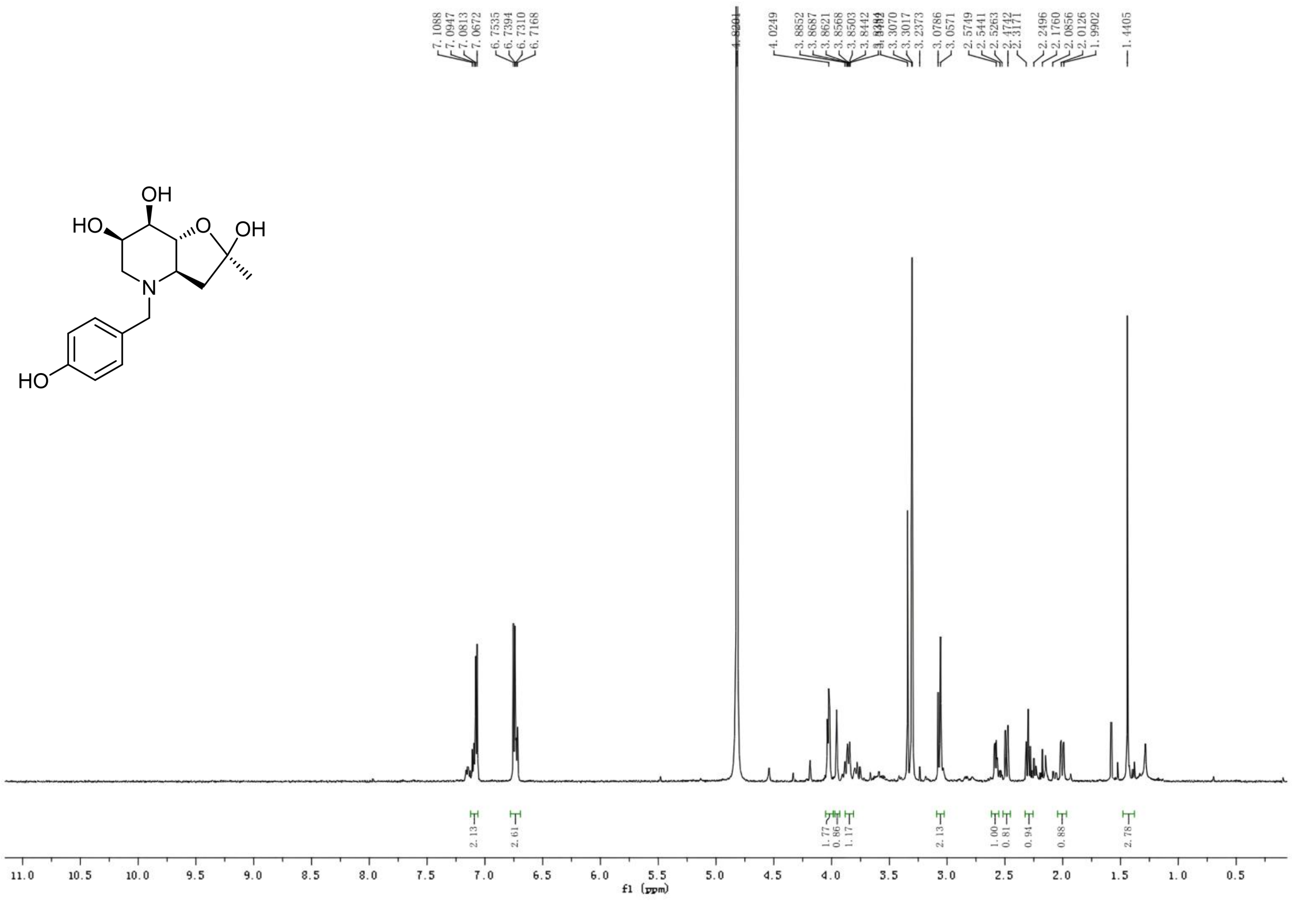

Figure S23. The ^{1}H NMR of compound **14**.

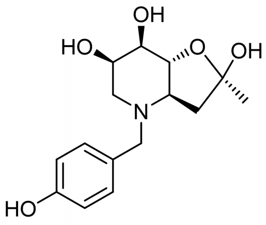

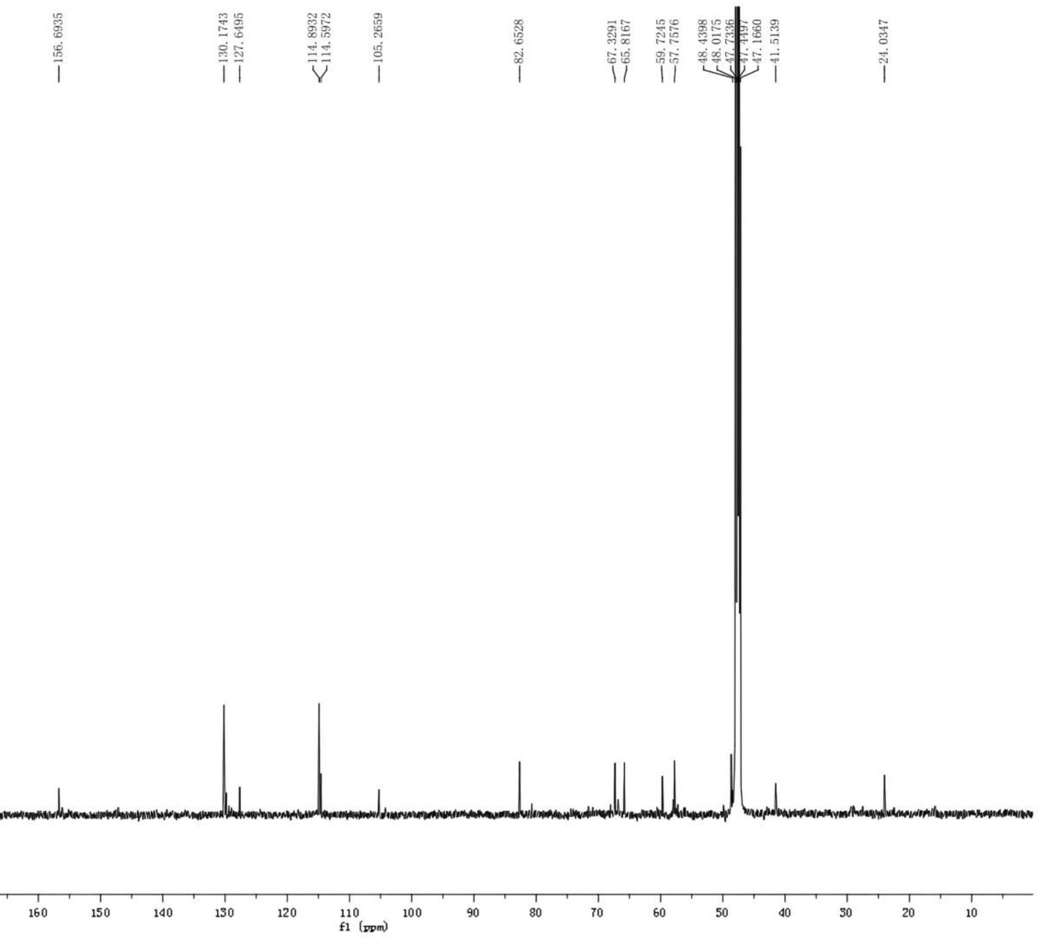

Figure S24. The ^{13}C-NMR of Compound **14**.

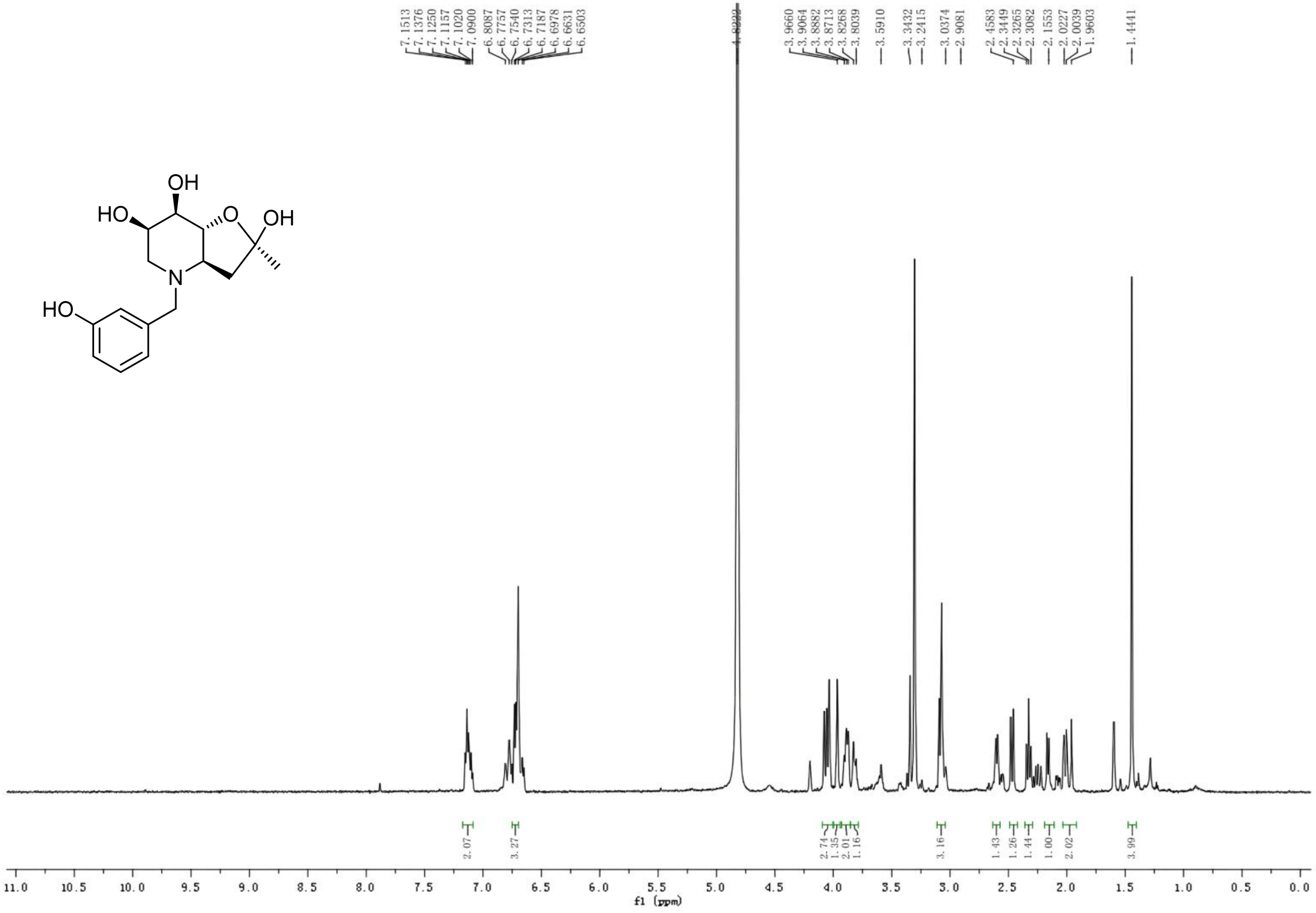

Figure S25. The ^{1}H NMR of compound **16**.

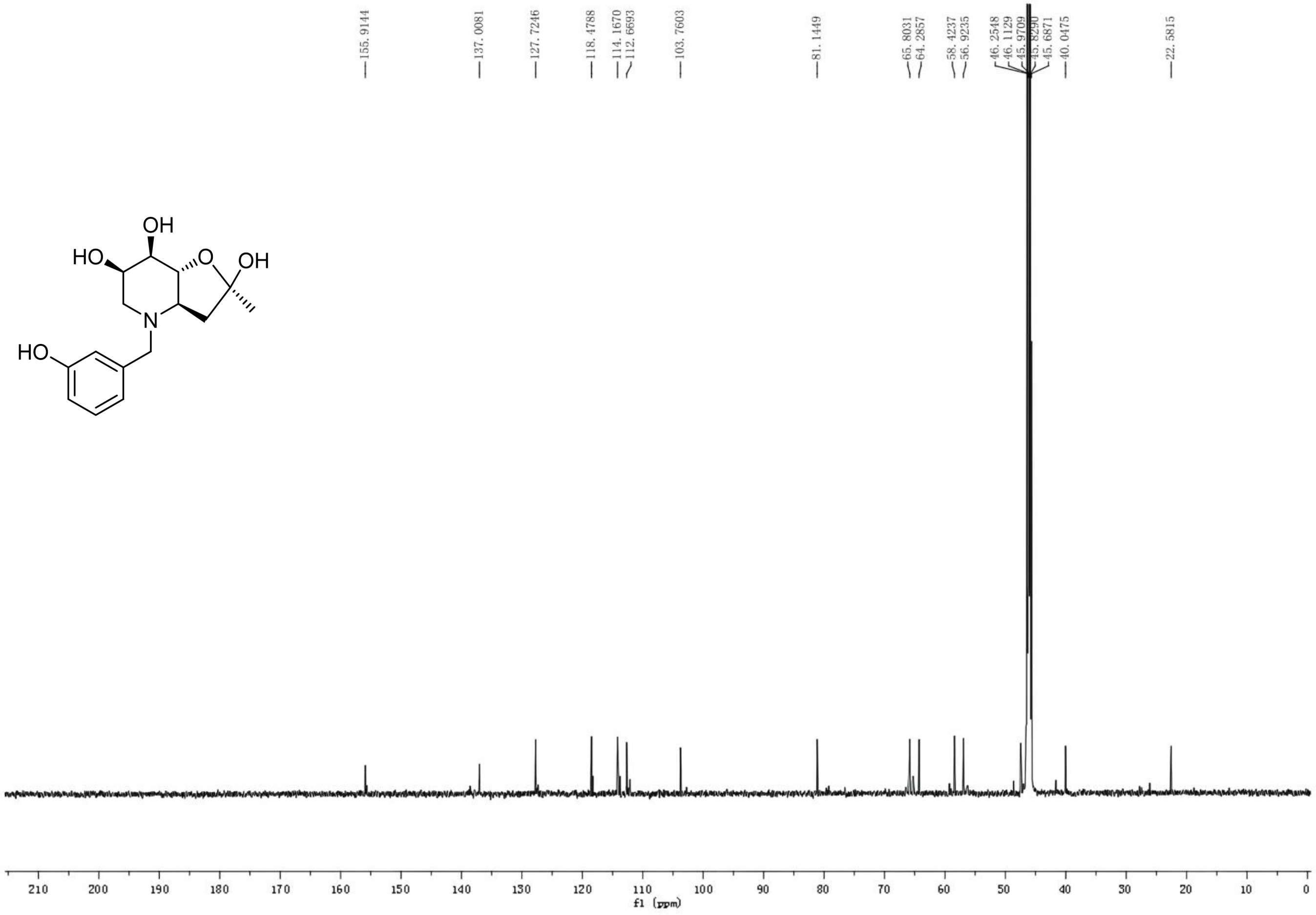

Figure S26. The ^{13}C-NMR of Compound **16**.

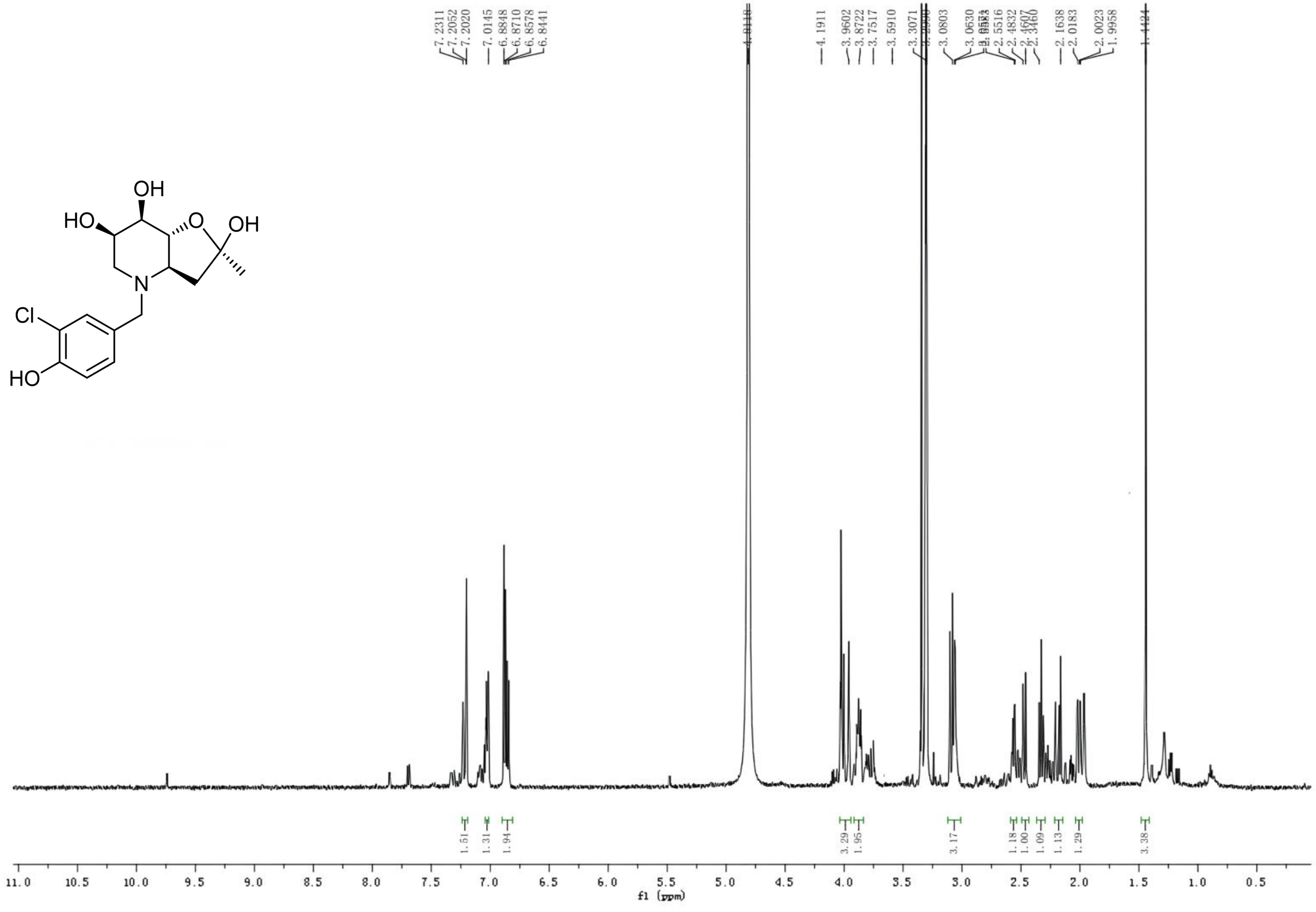

Figure S27. The ^{1}H NMR of compound **17**.

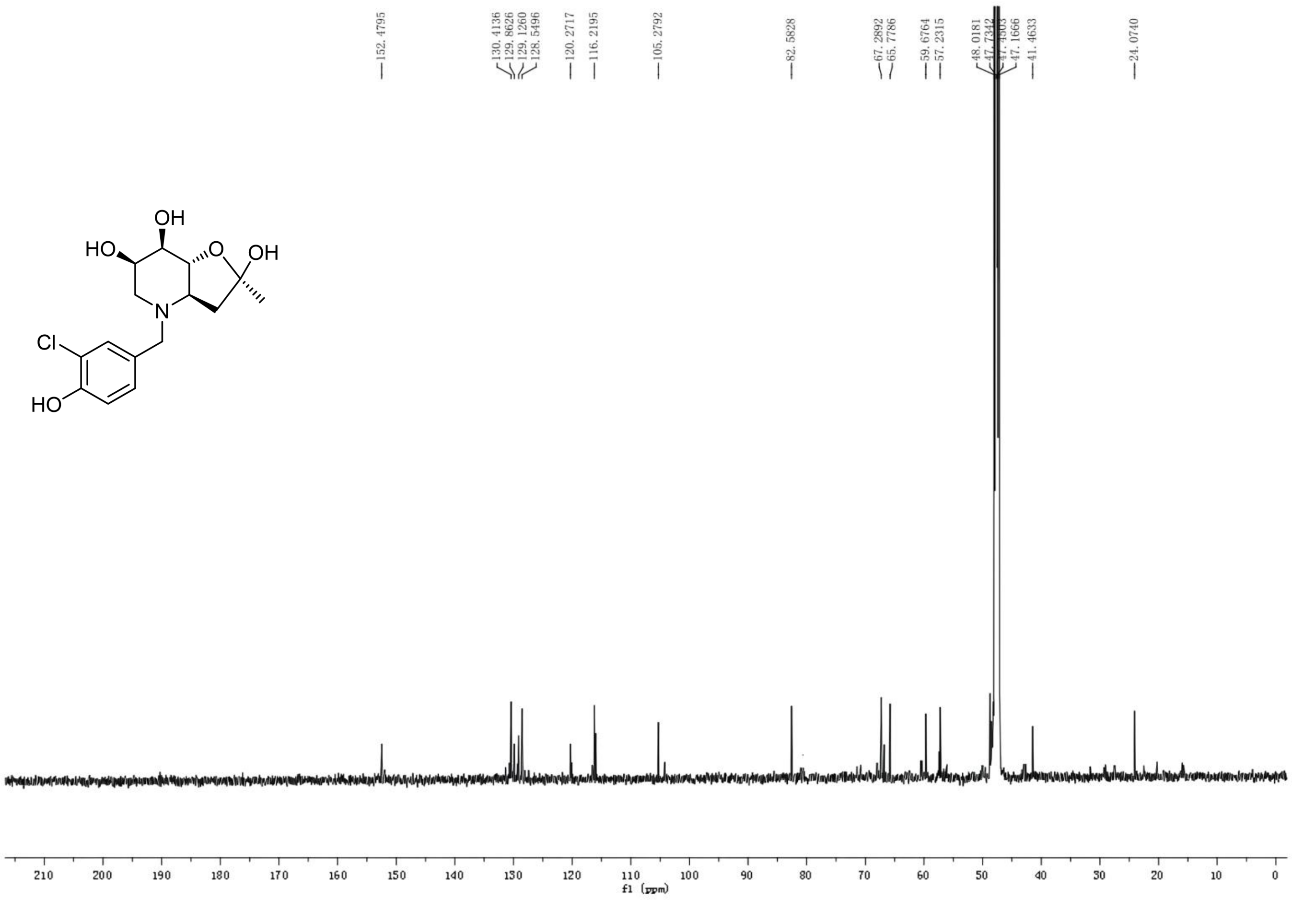

Figure S28. The ^{13}C-NMR of Compound **17**.

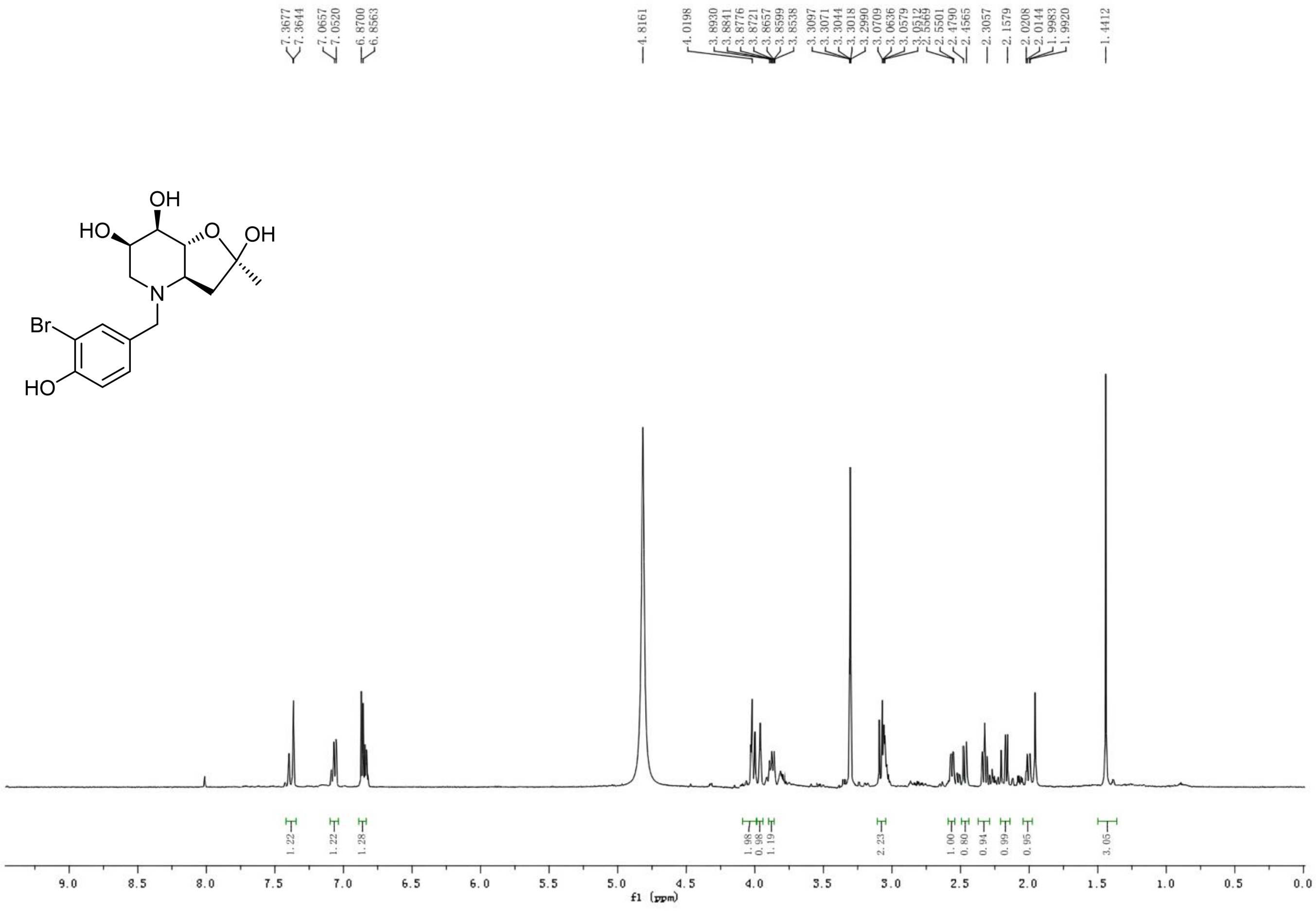

Figure S29. The ^{1}H NMR of compound **18**.

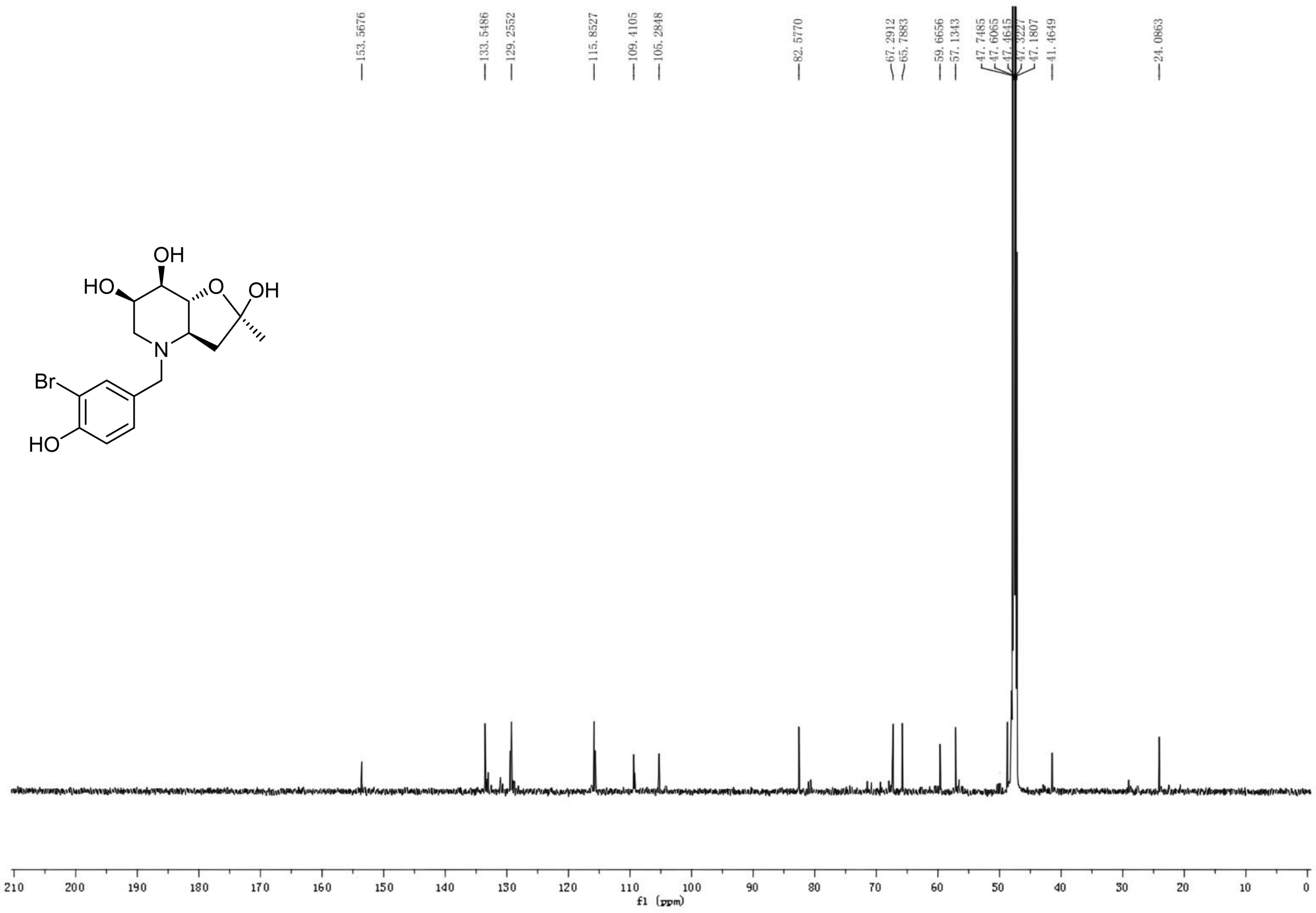

Figure S30. The ^{13}C-NMR of Compound **18**.

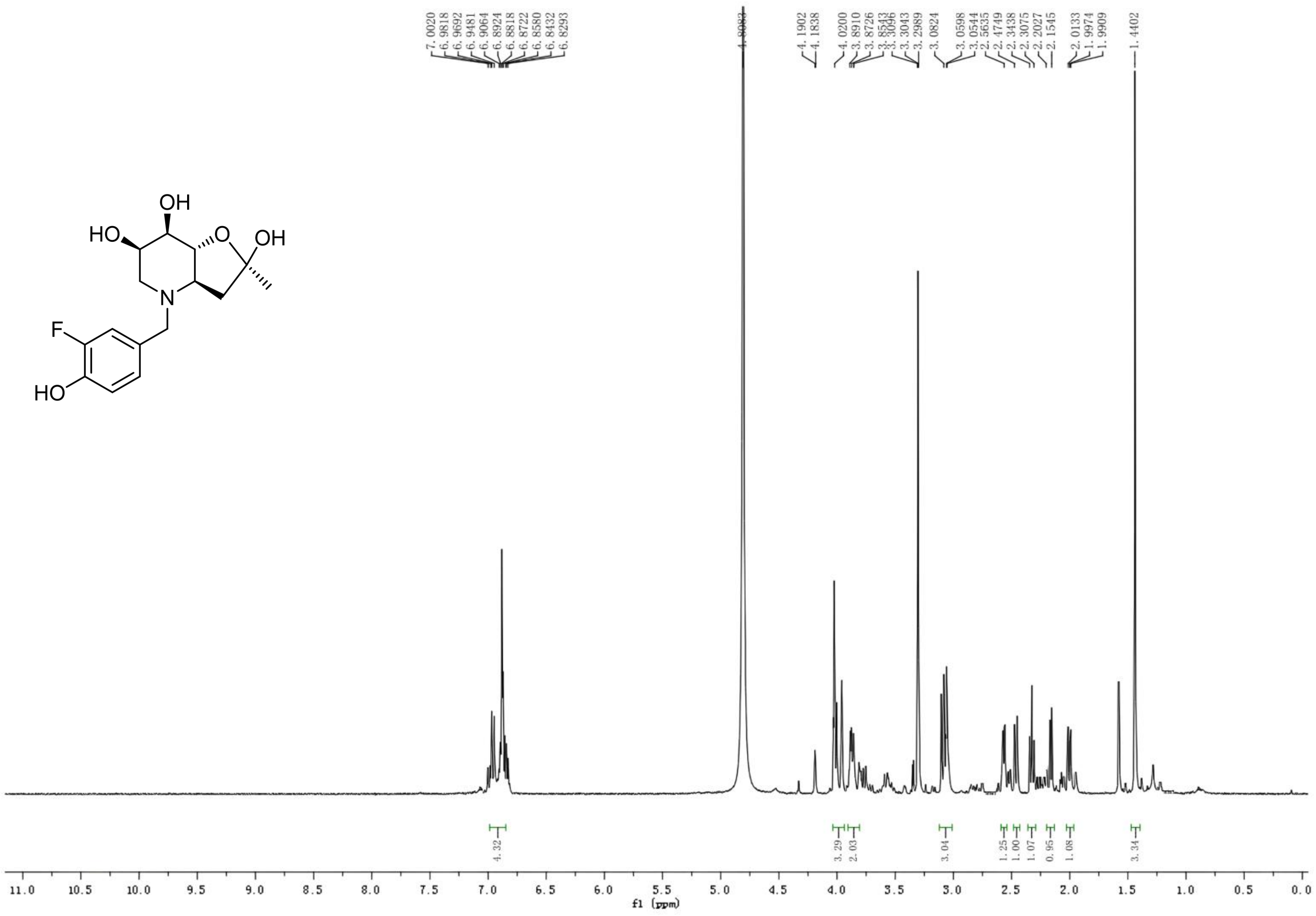

Figure S31. The ^{1}H NMR of compound **19**.

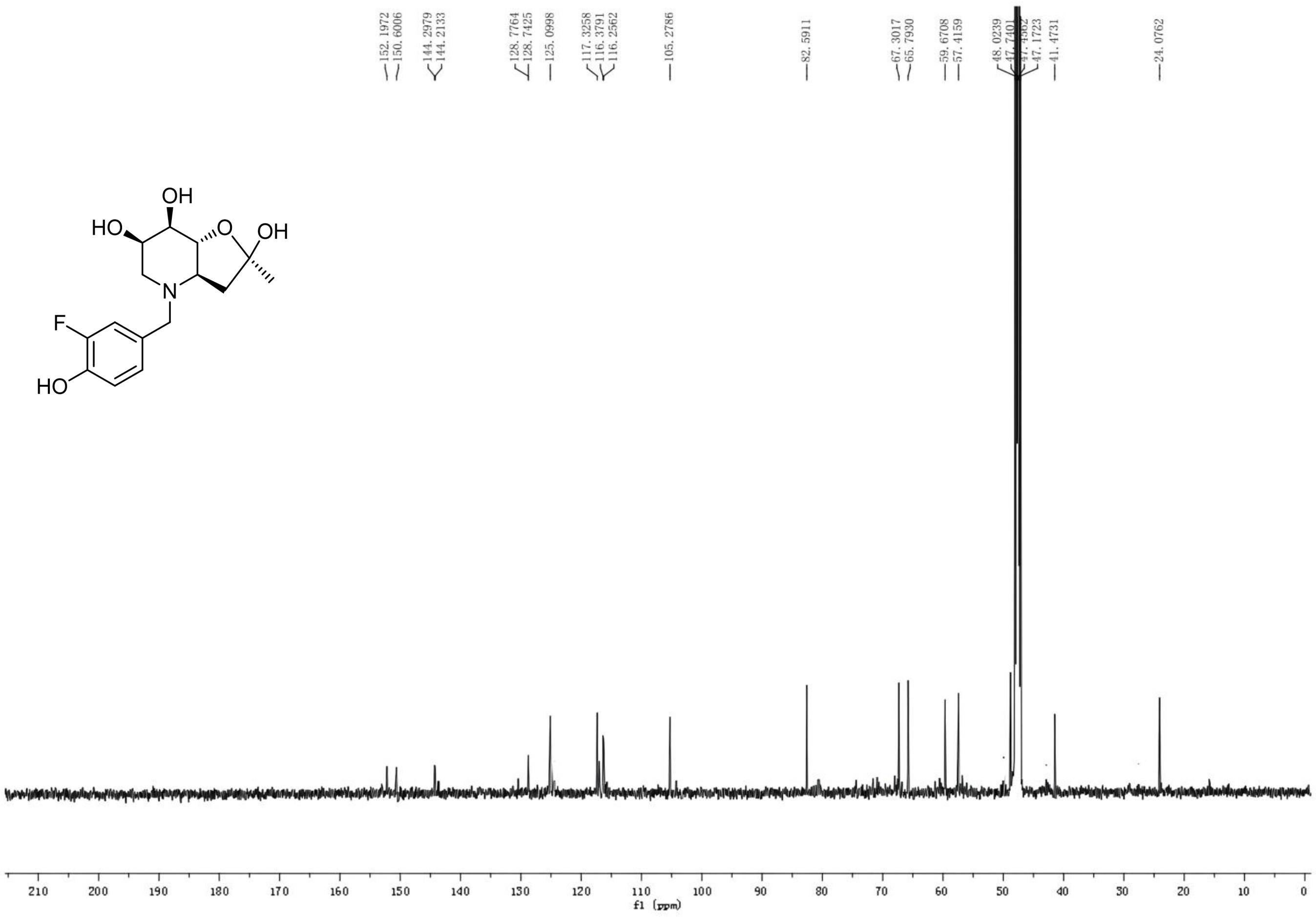

Figure S32. The ^{13}C-NMR of Compound **19**.

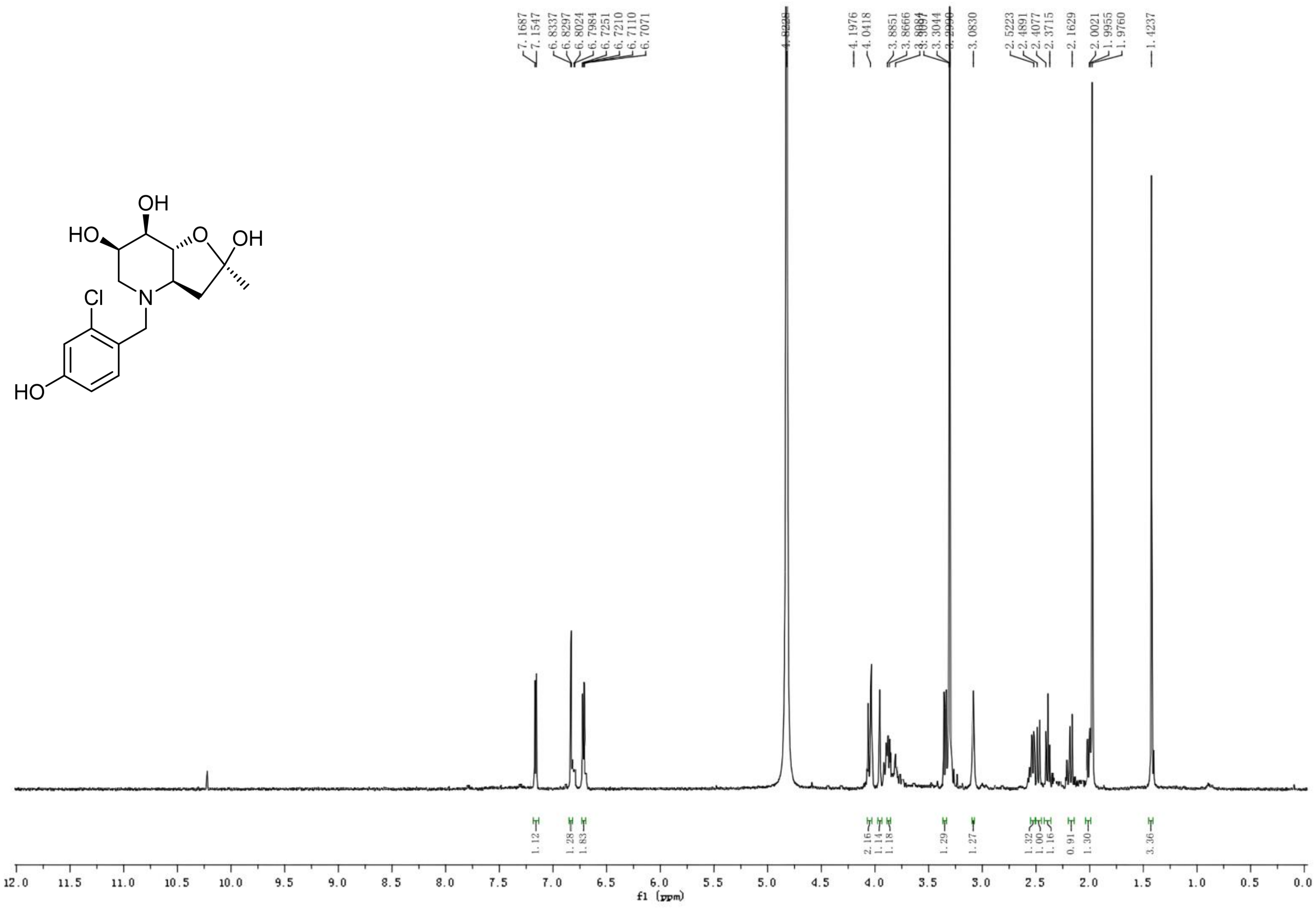

Figure S33. The ^{1}H NMR of compound **20**.

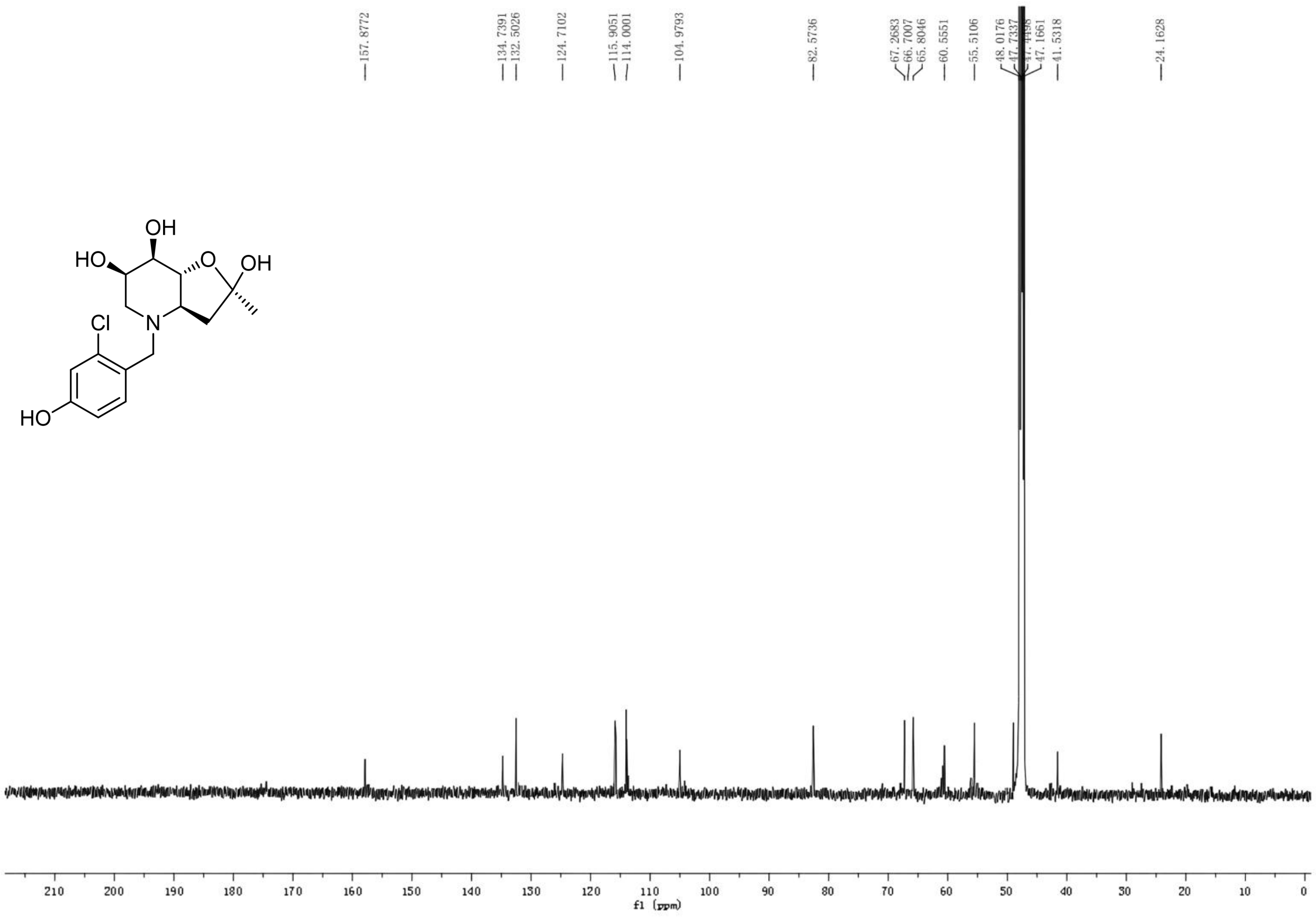

Figure S34. The ^{13}C-NMR of Compound **20**.

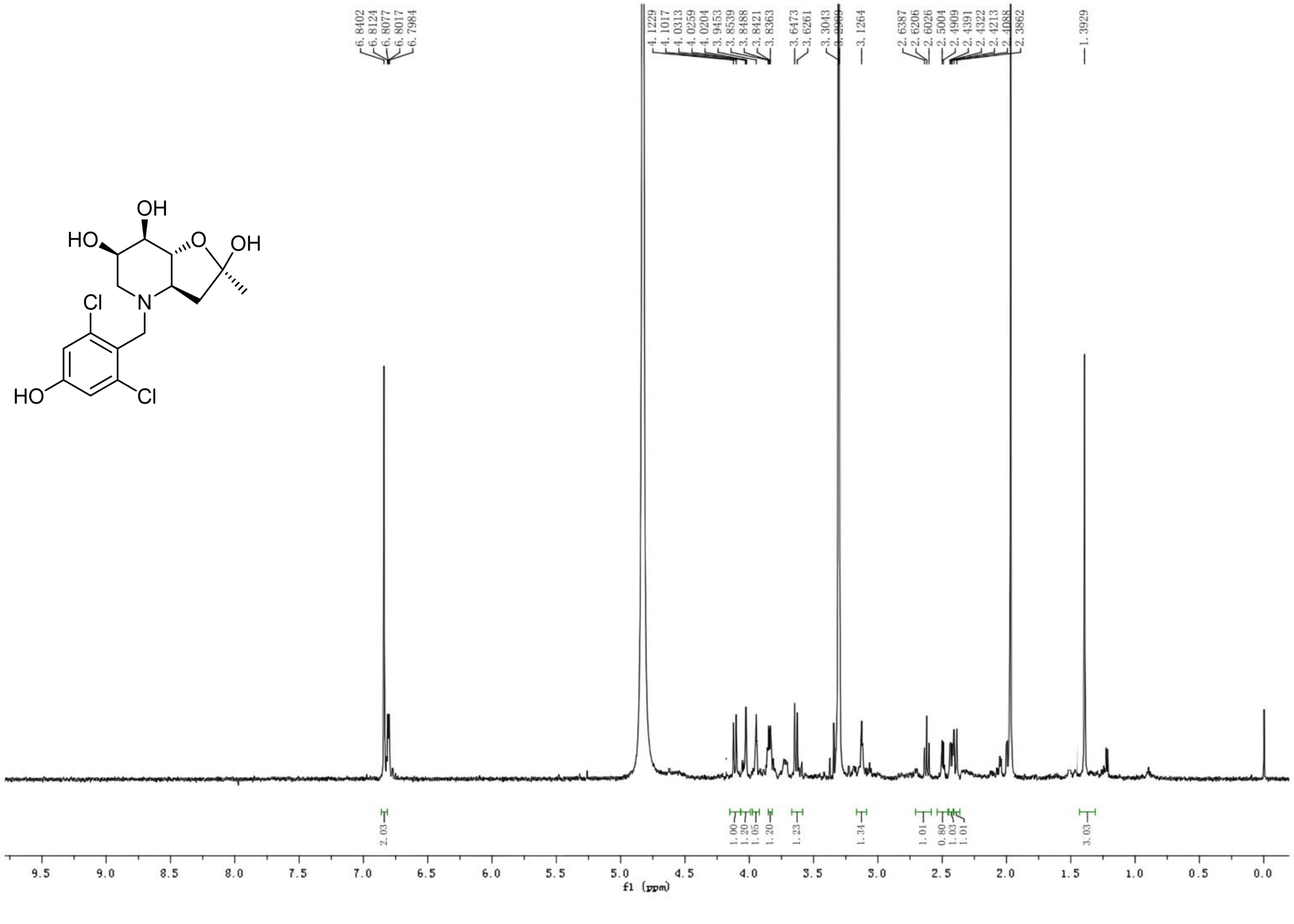

Figure S35. The ^{1}H NMR of compound **21**.

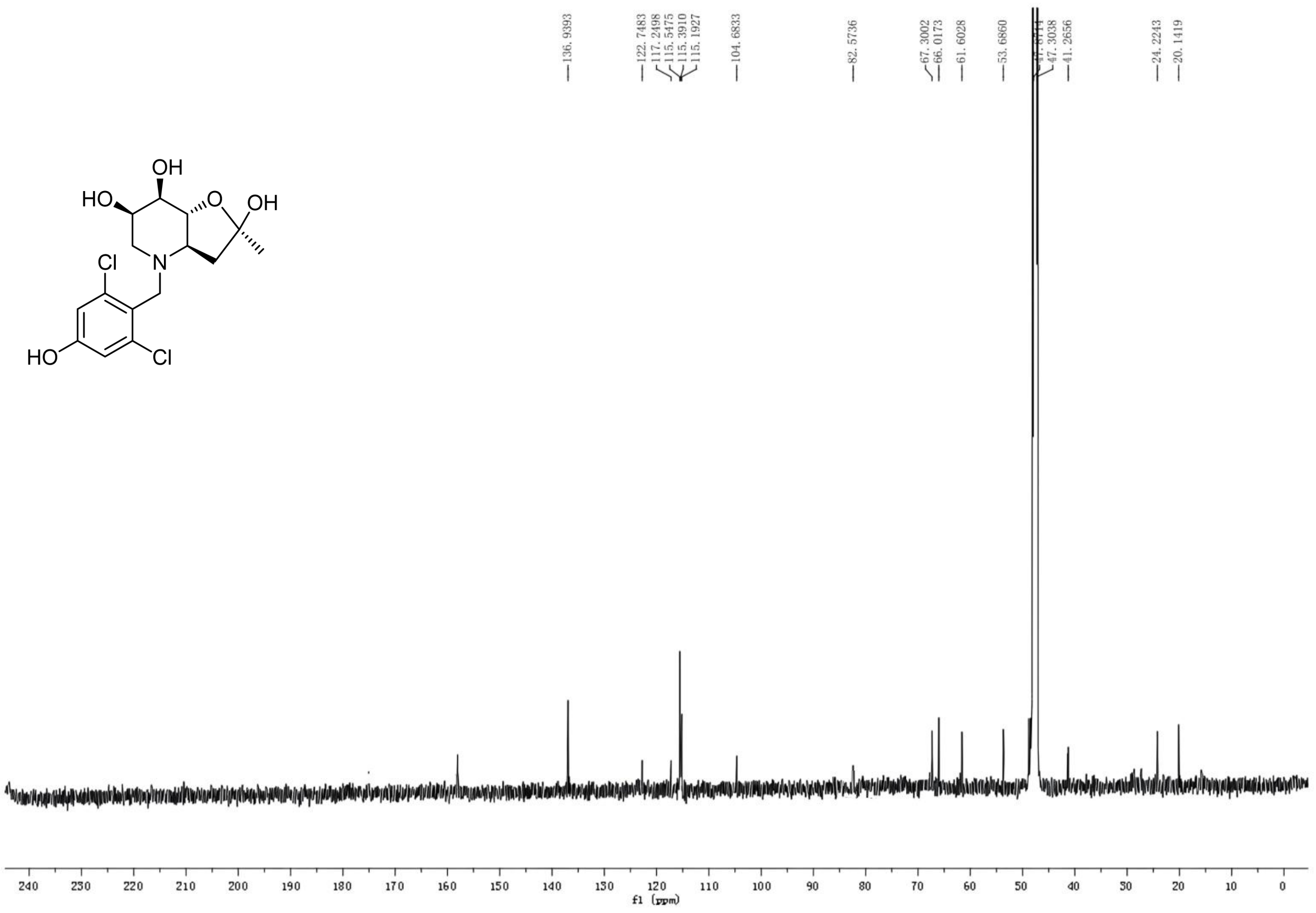

Figure S36. The ^{13}C-NMR of Compound **21**.

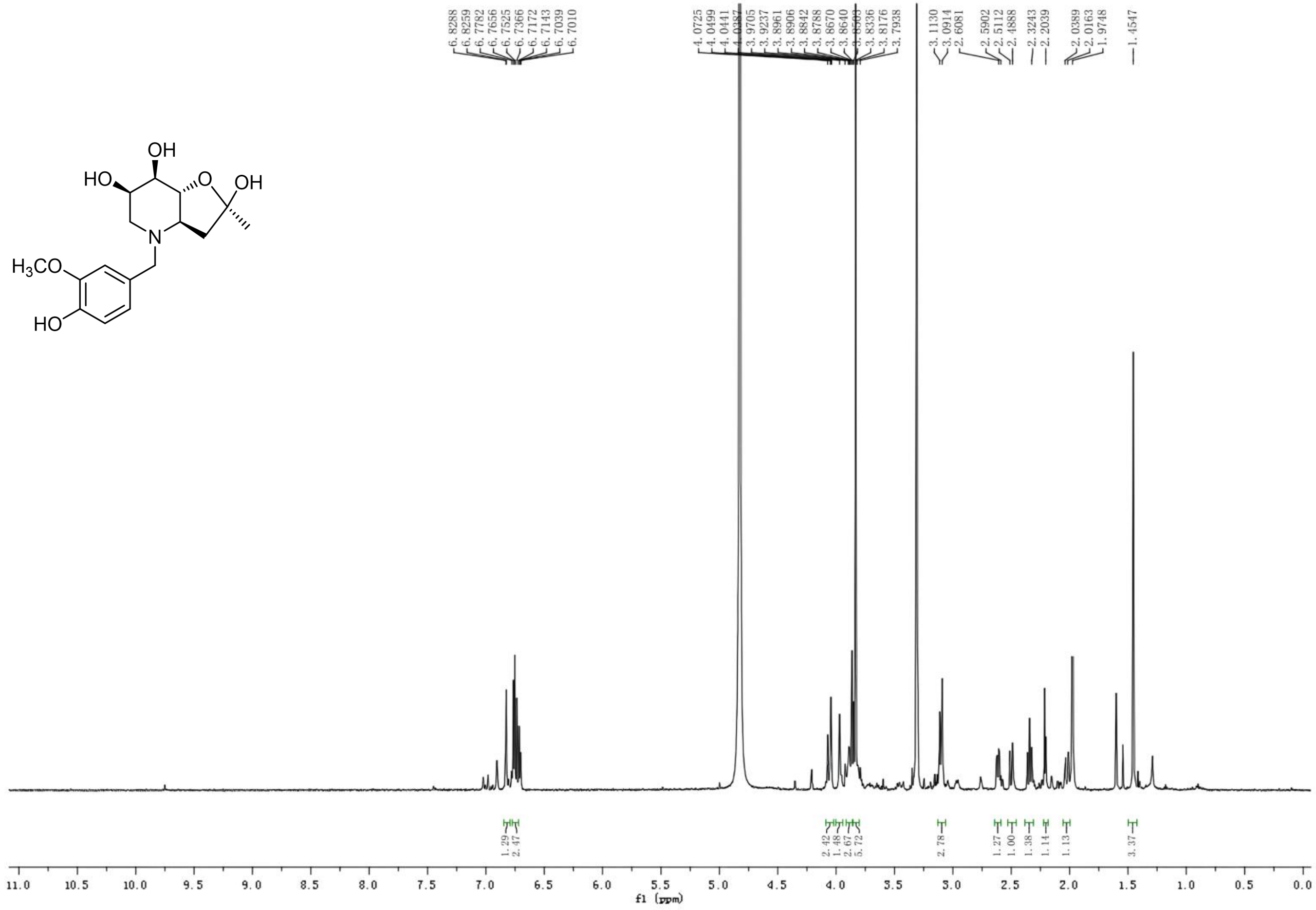

Figure S37. The ^{1}H NMR of compound **22**.

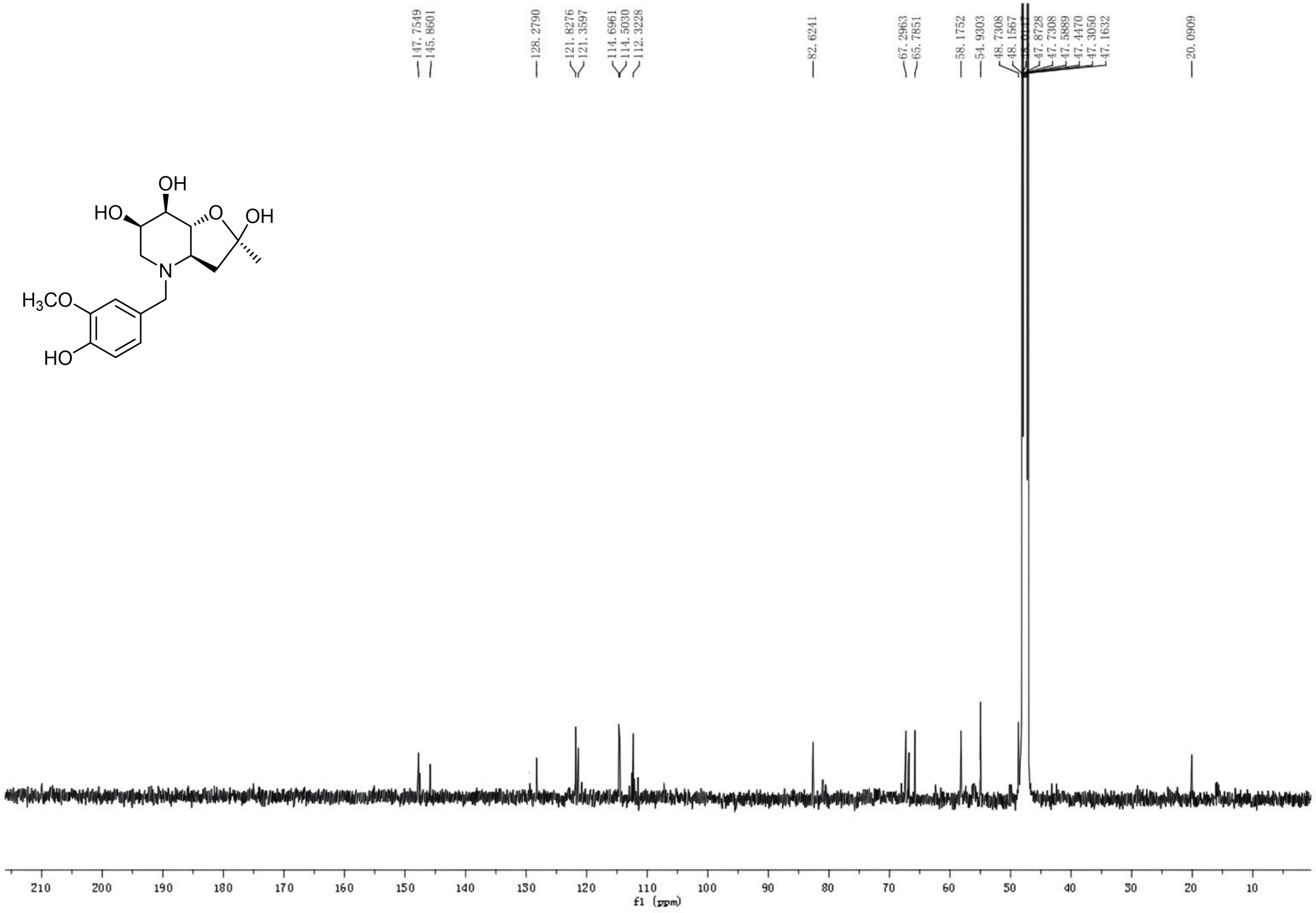

Figure S38. The ^{13}C-NMR of Compound **22**.

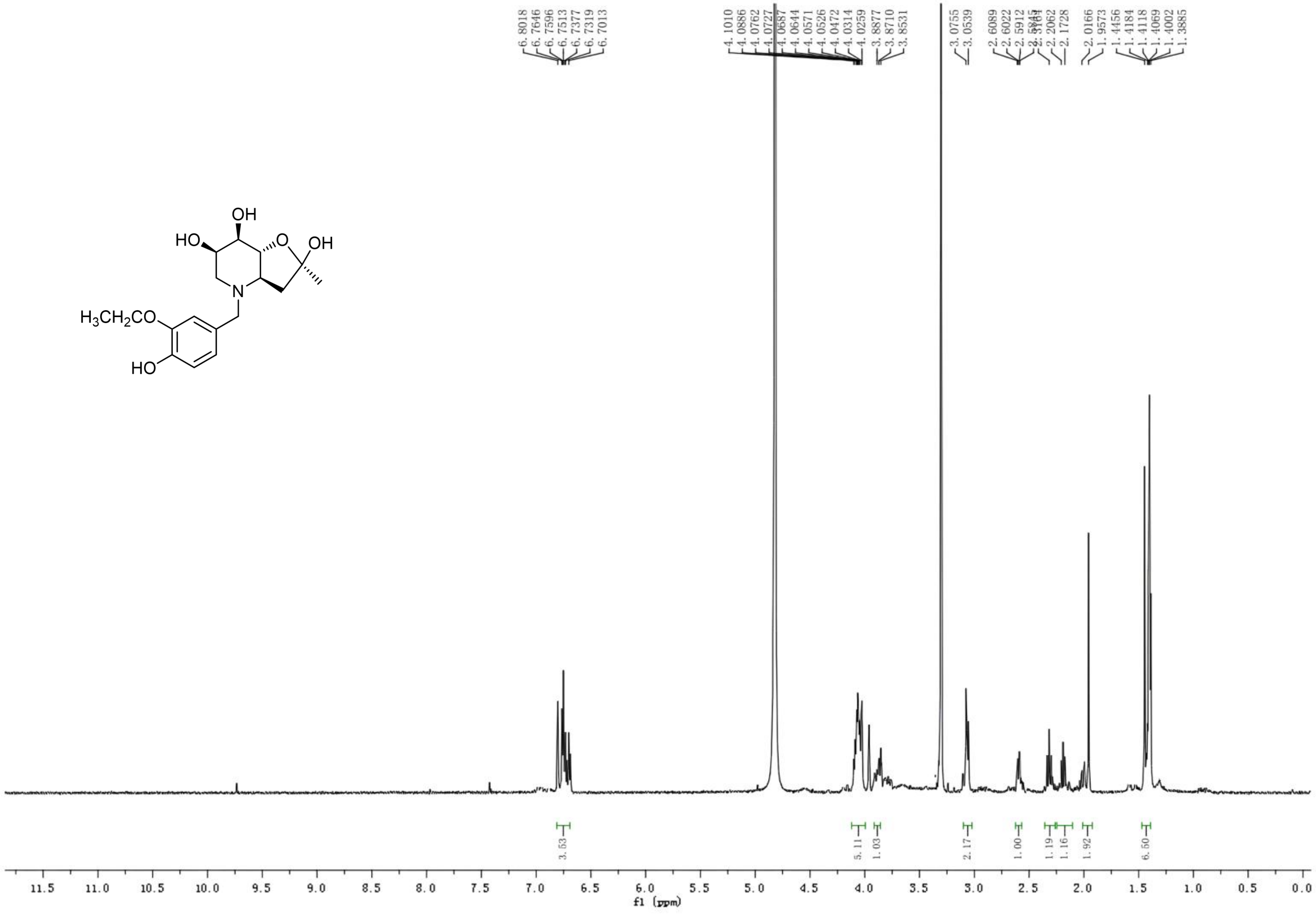

Figure S39. The ^{1}H NMR of compound **23**.

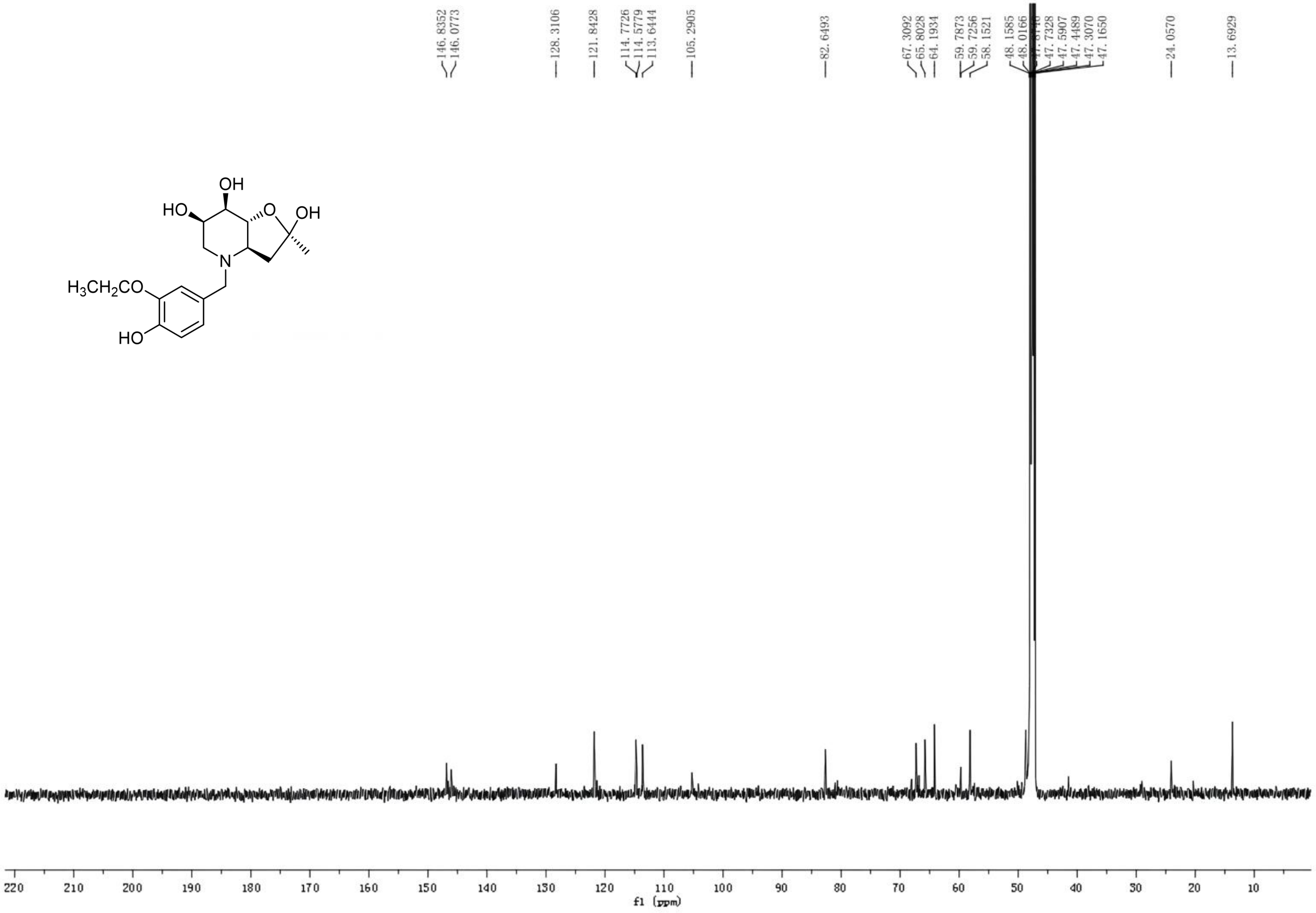

Figure S40. The ^{13}C-NMR of Compound **23**.

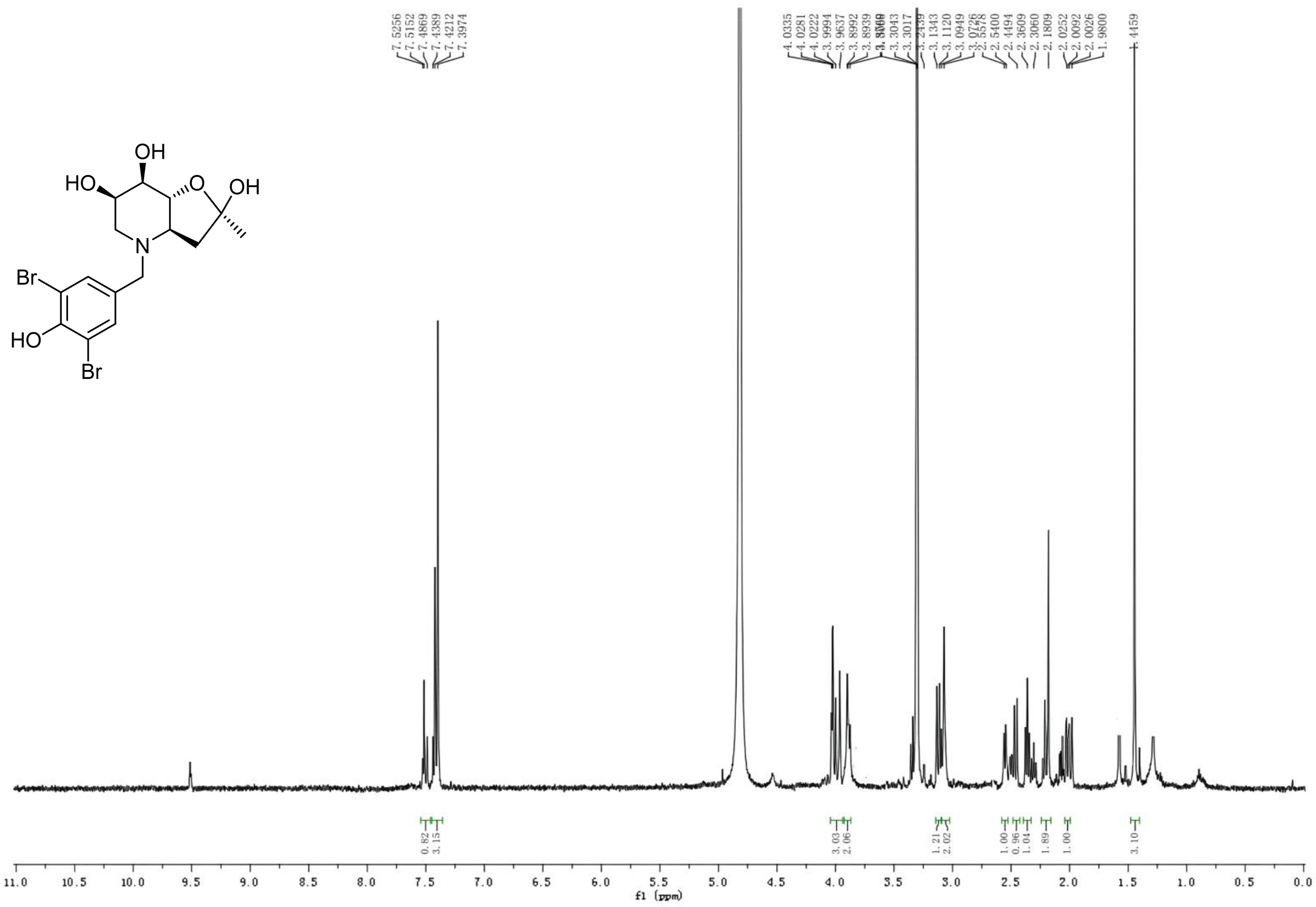

Figure S41. The ^{1}H NMR of compound **24**.

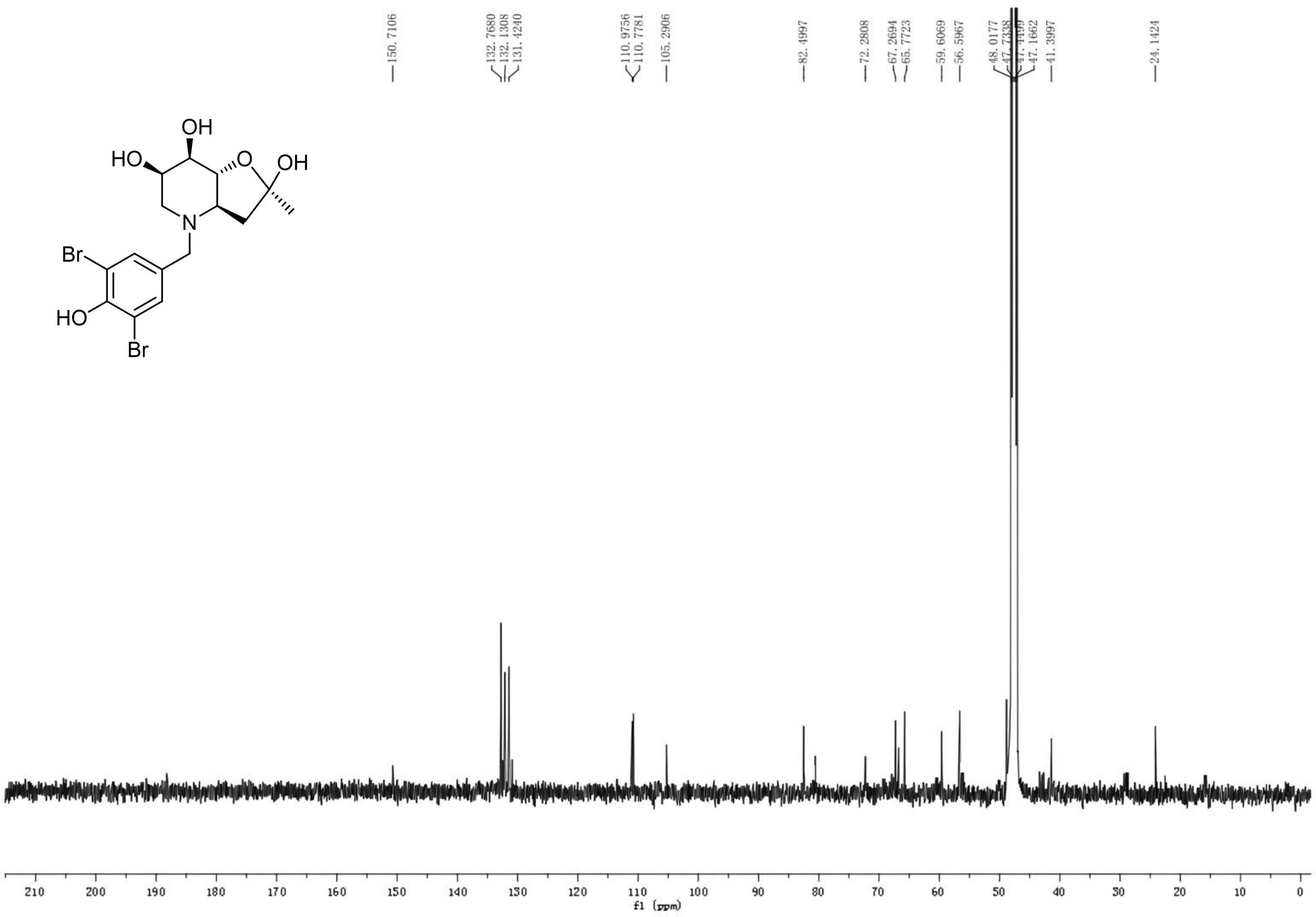

Figure S42. The ^{13}C-NMR of Compound **24**.

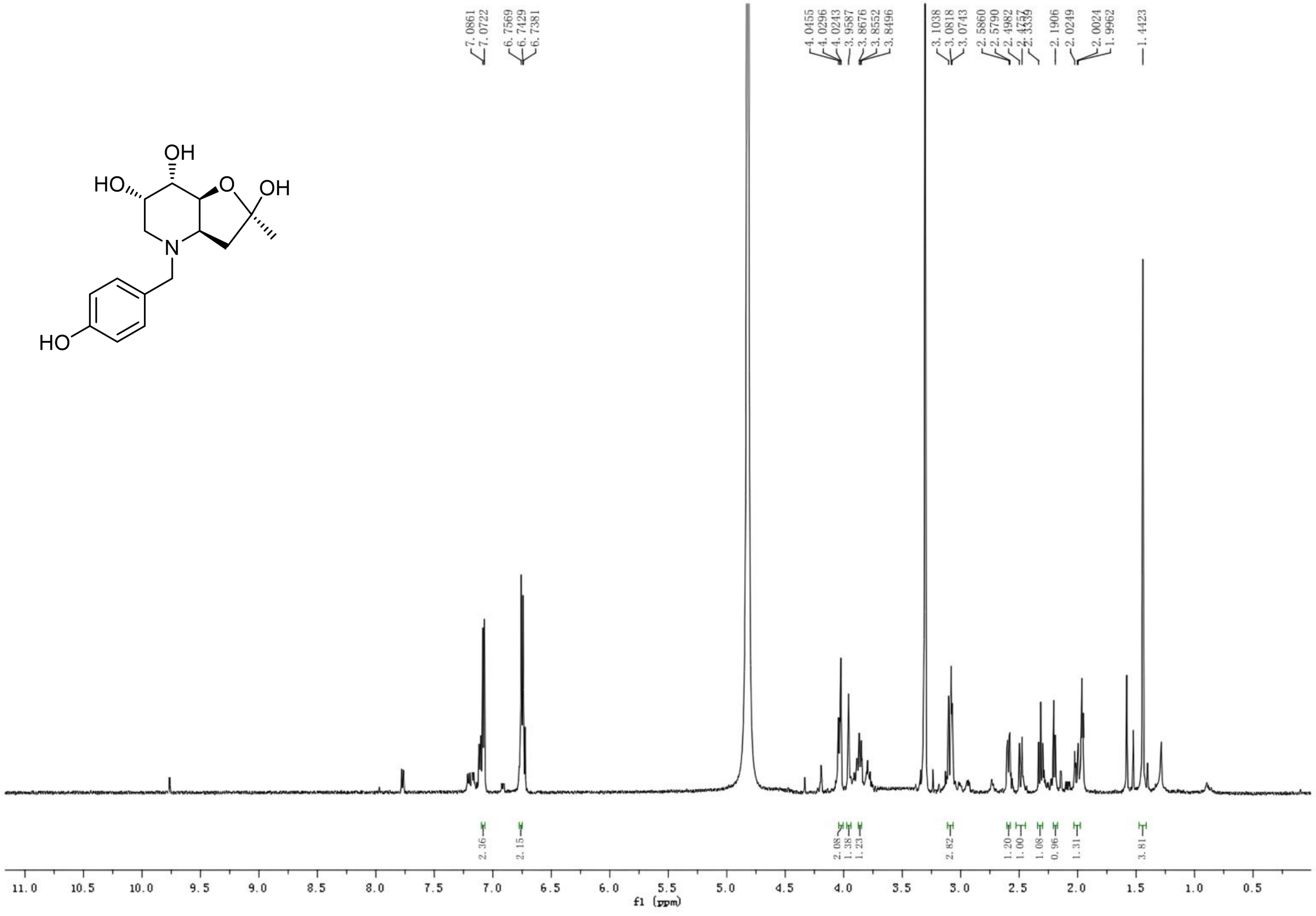

Figure S43. The ^{1}H NMR of compound **25**.

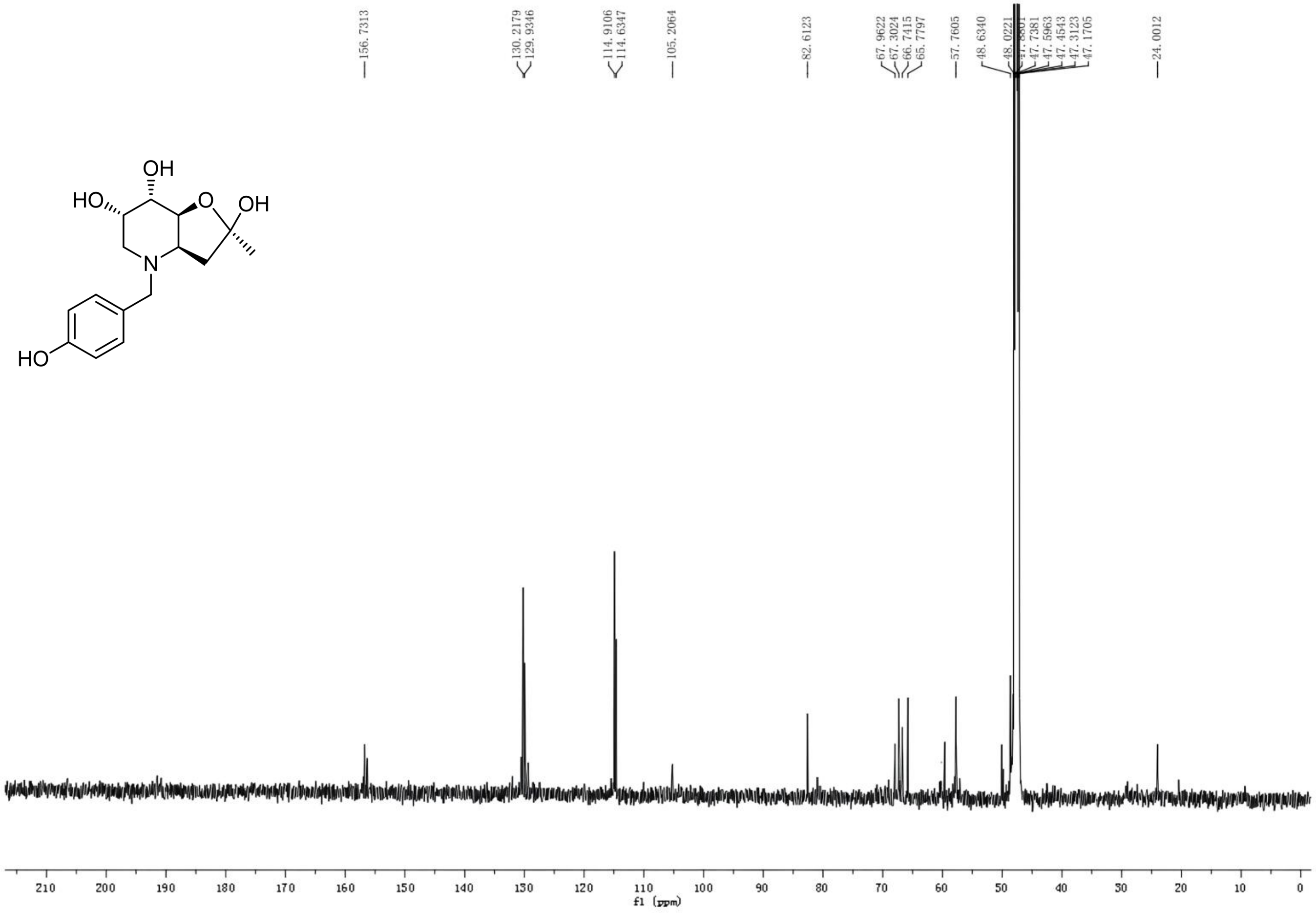

Figure S44. The ^{13}C-NMR of Compound **25**.

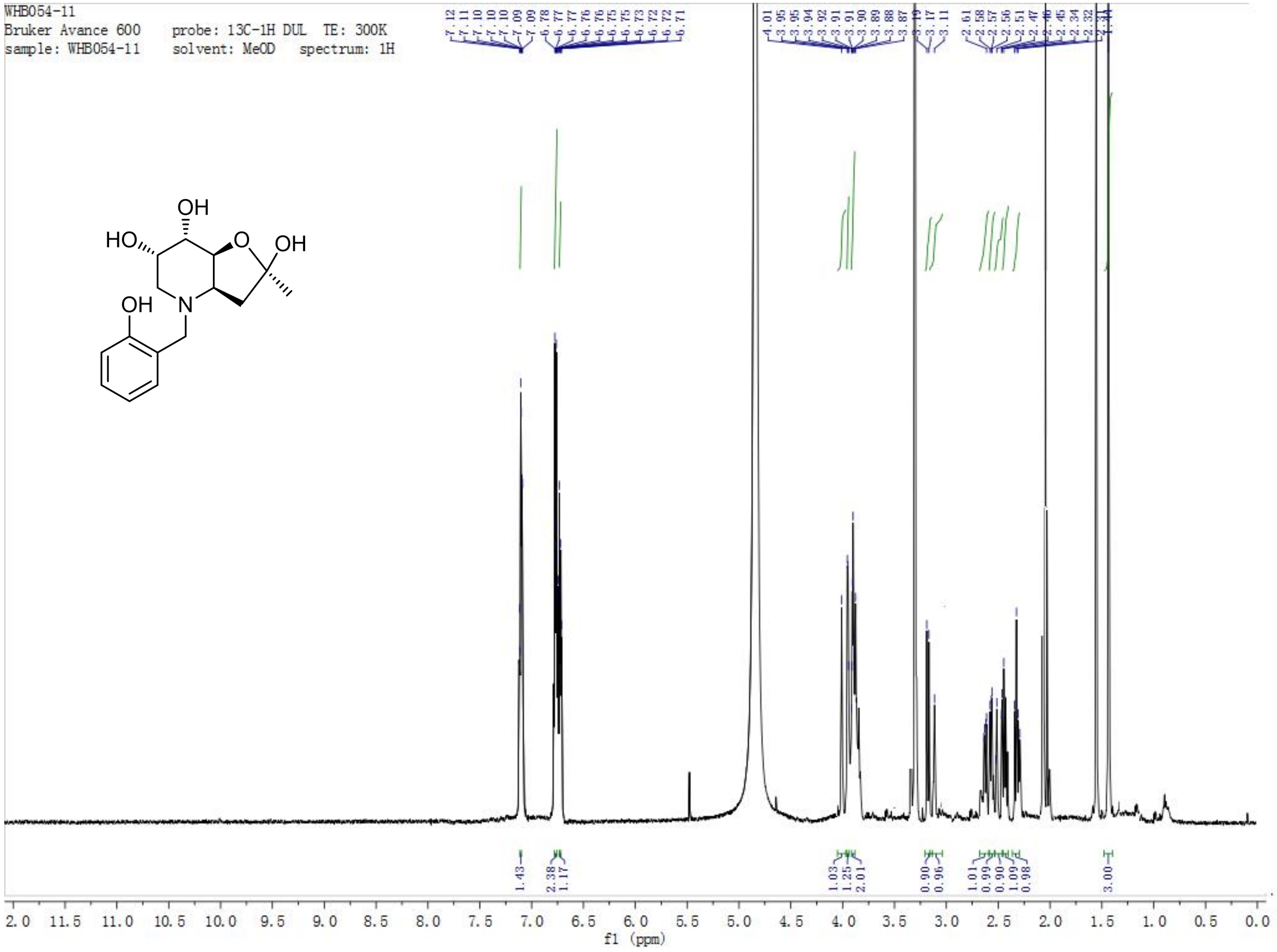

Figure S45. The ^{1}H NMR of compound **26**.

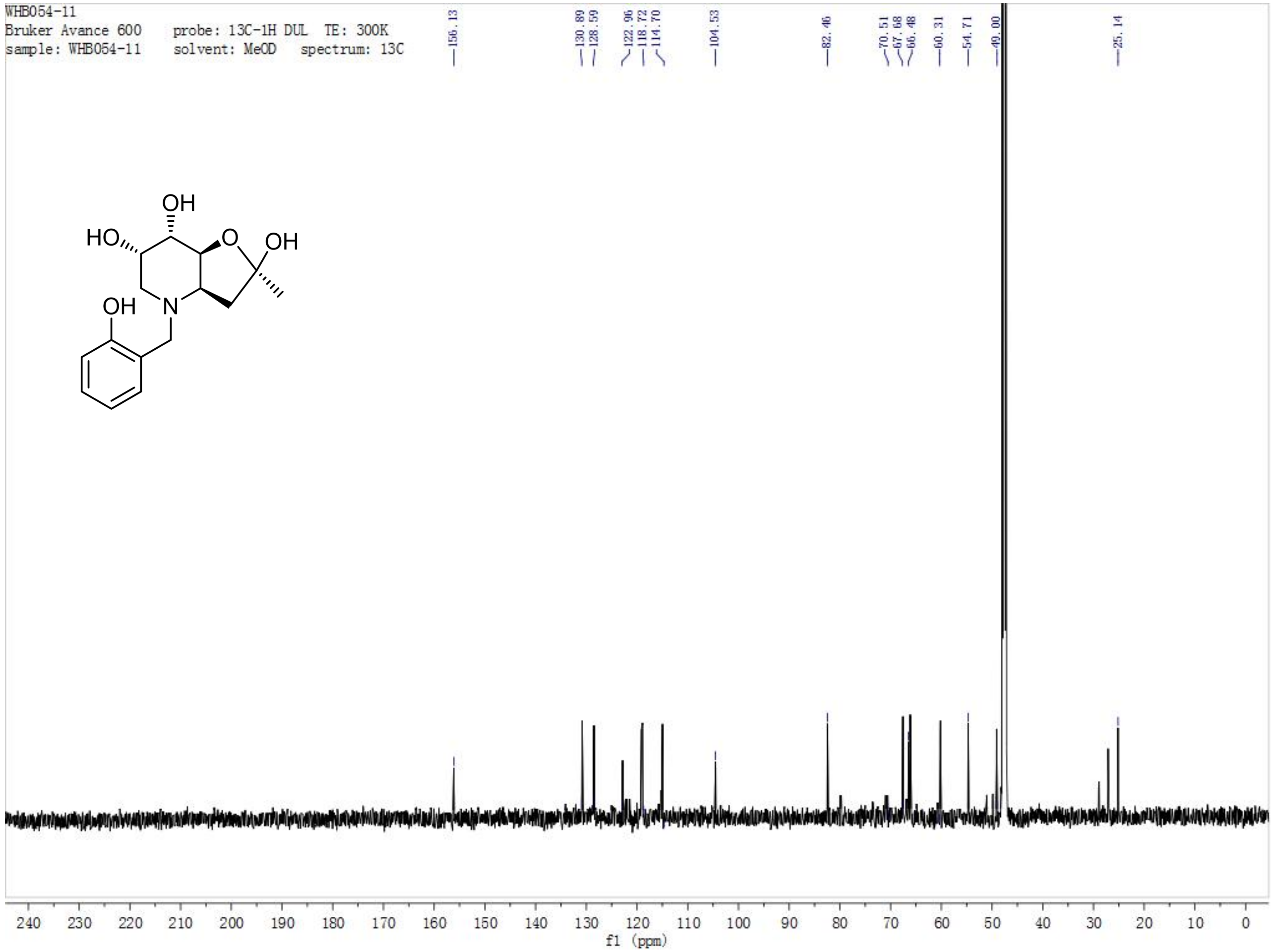

Figure S46. The ^{13}C-NMR of Compound **26**.

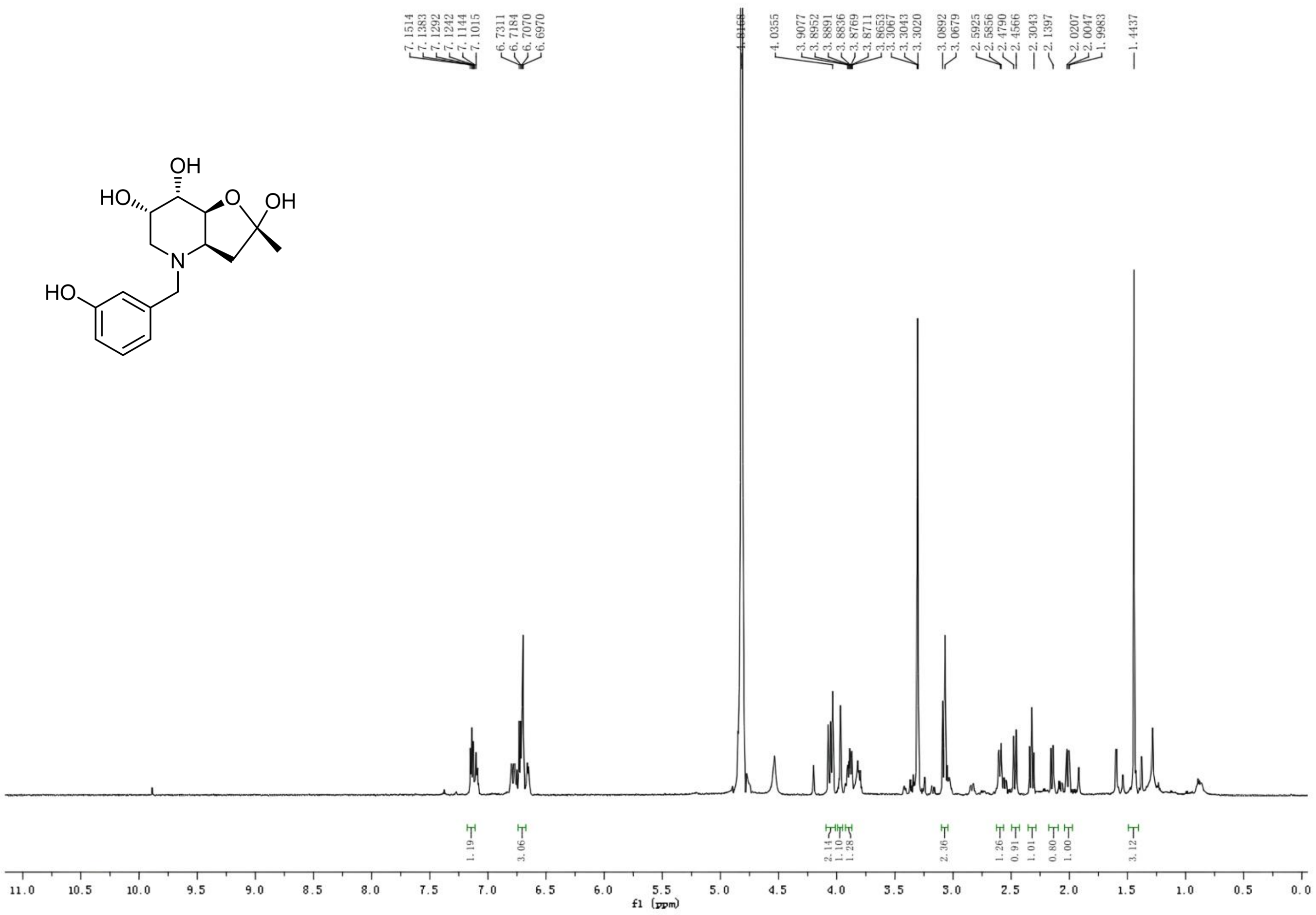

Figure S47. The ^{1}H NMR of compound **27**.

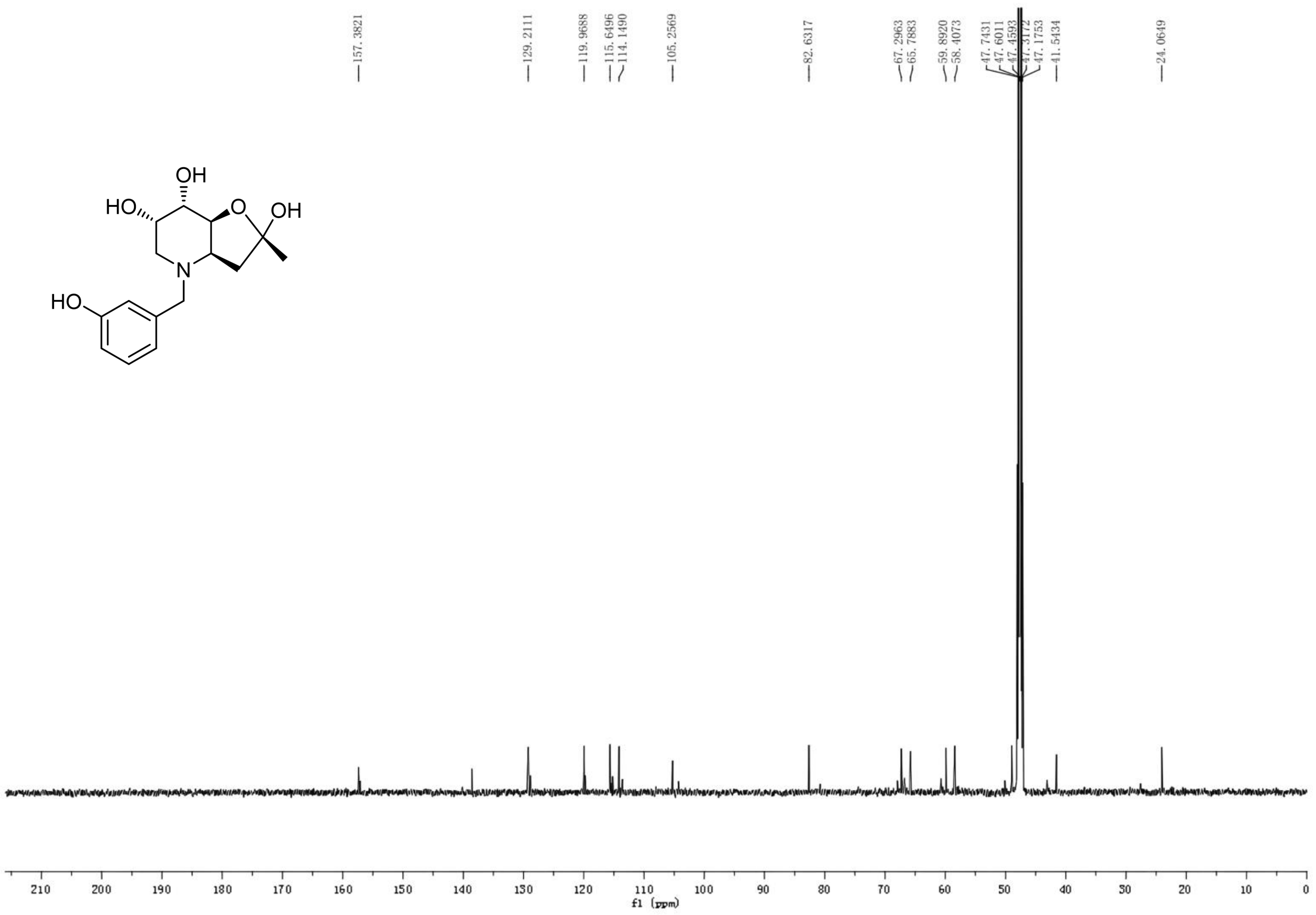

Figure S48. The ^{13}C-NMR of Compound **27**.

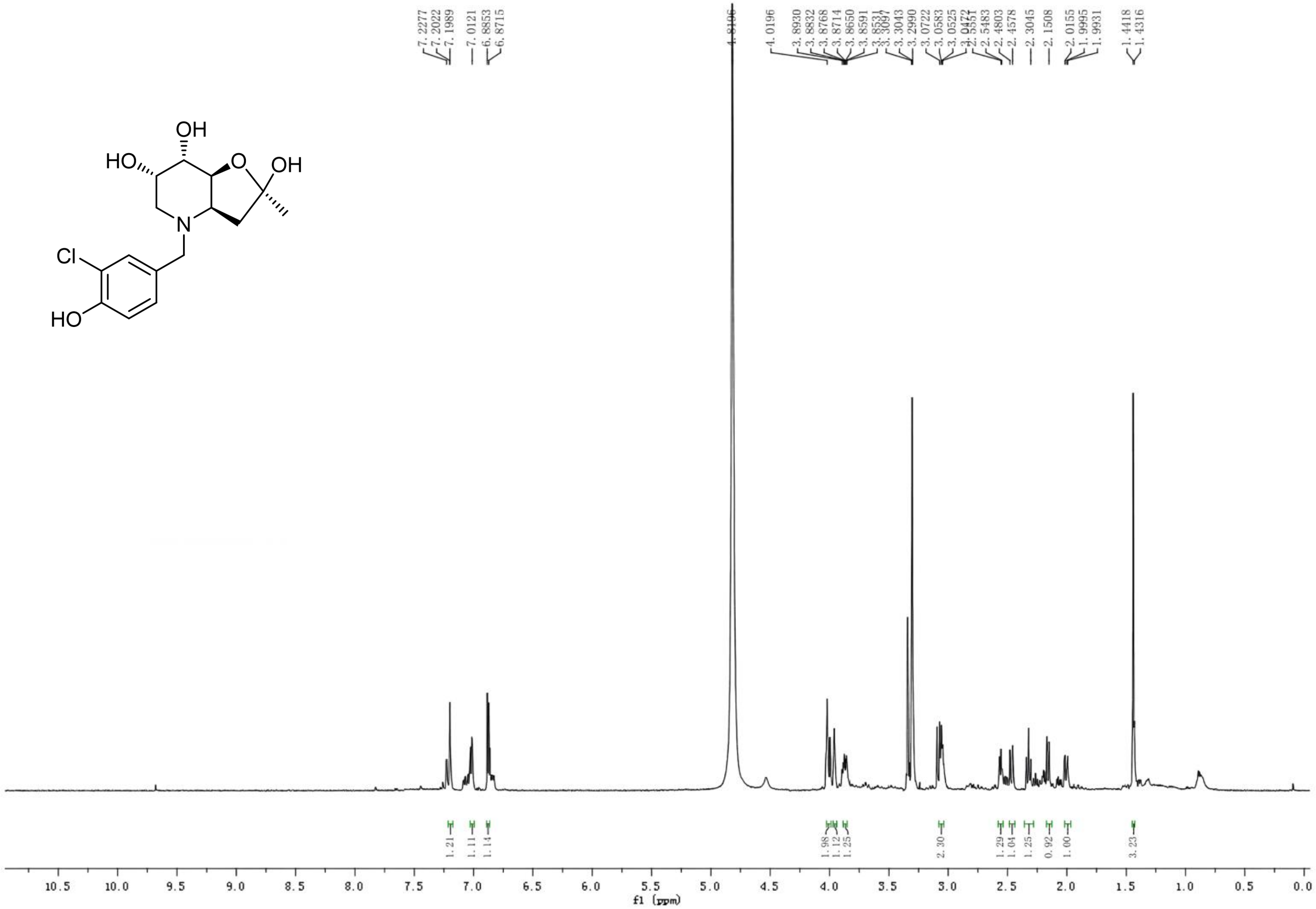

Figure S49. The ^{1}H NMR of compound **28**.

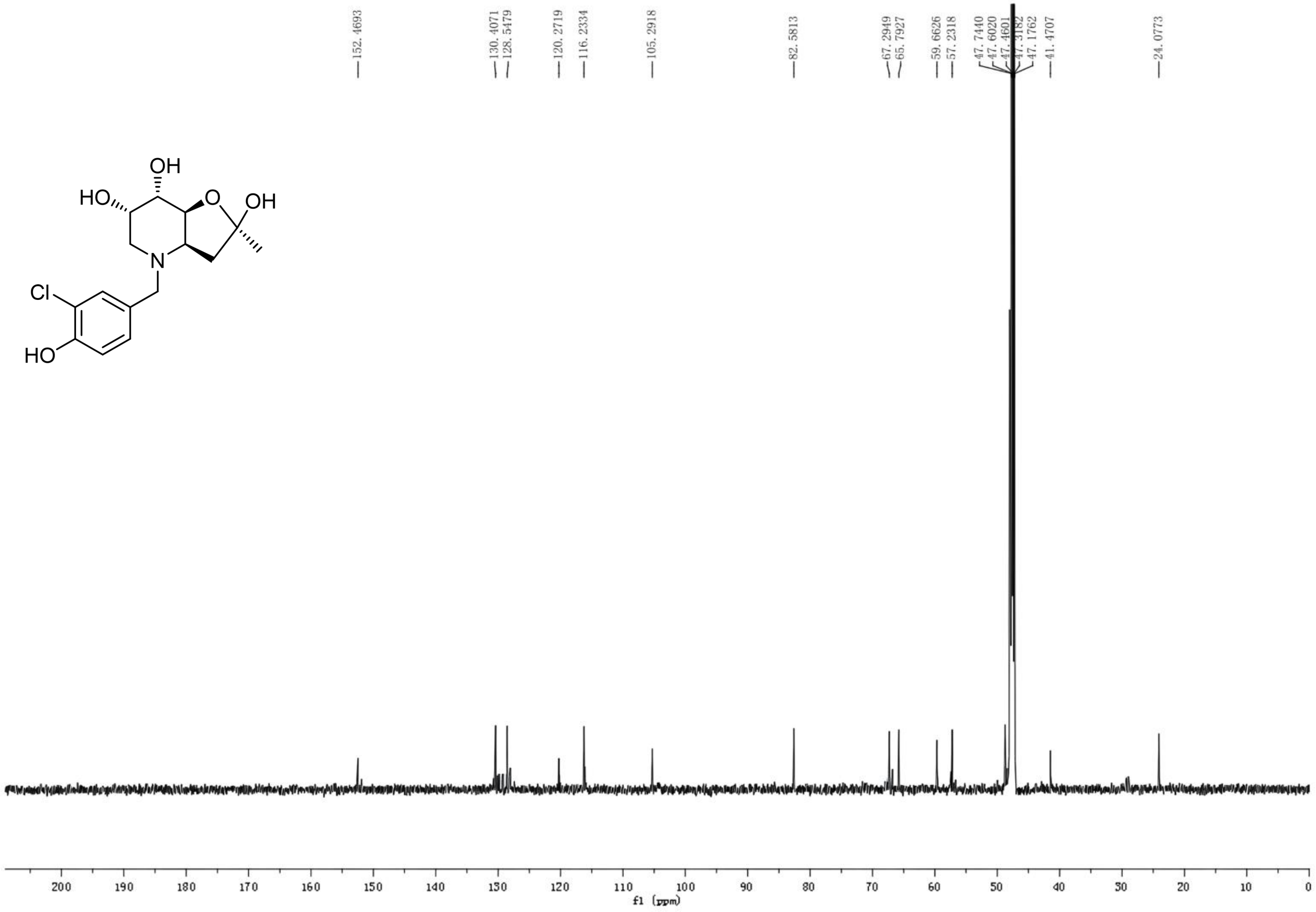

Figure S50. The ^{13}C-NMR of Compound **28**.

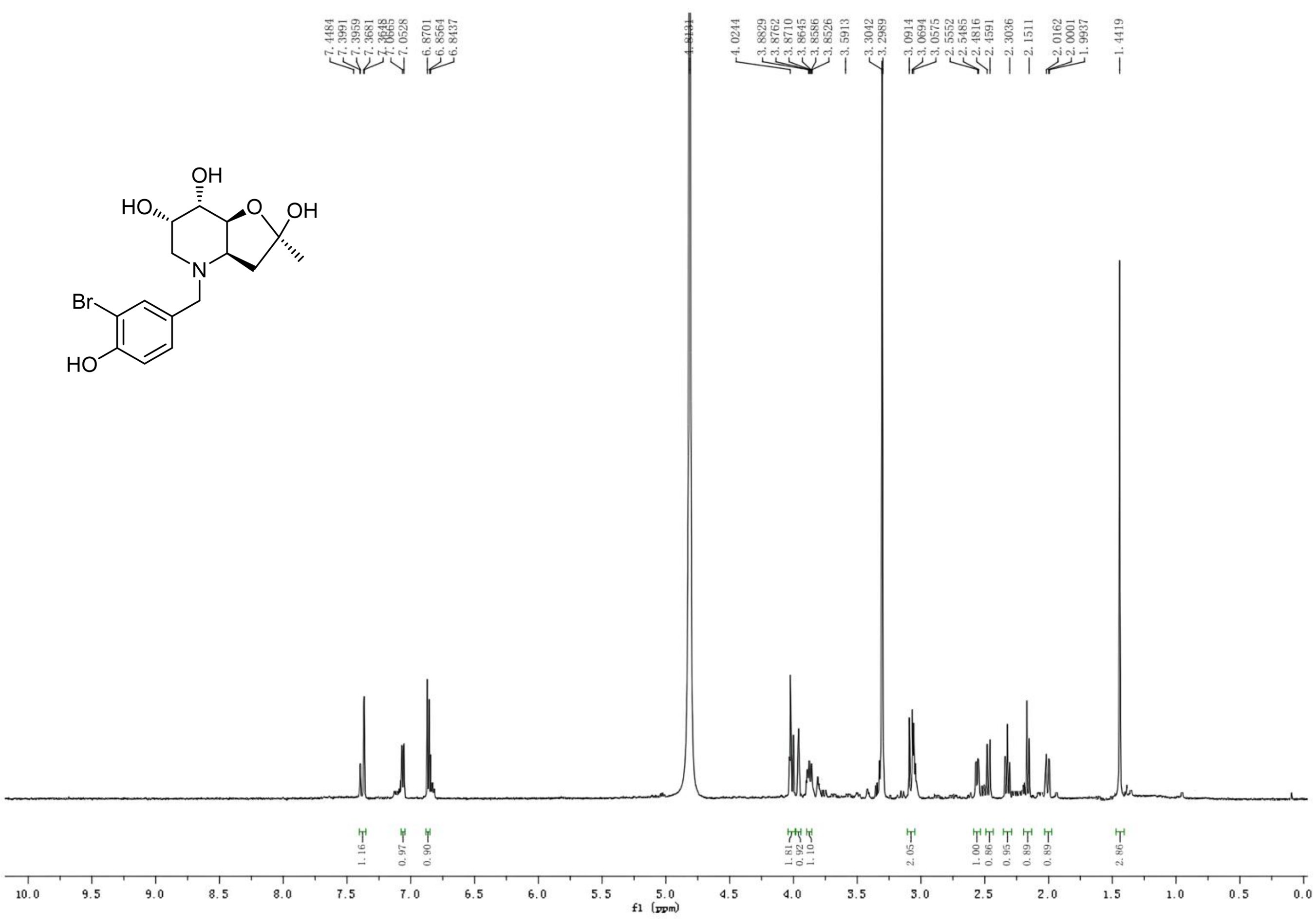

Figure S51. The ^{1}H NMR of compound **29**.

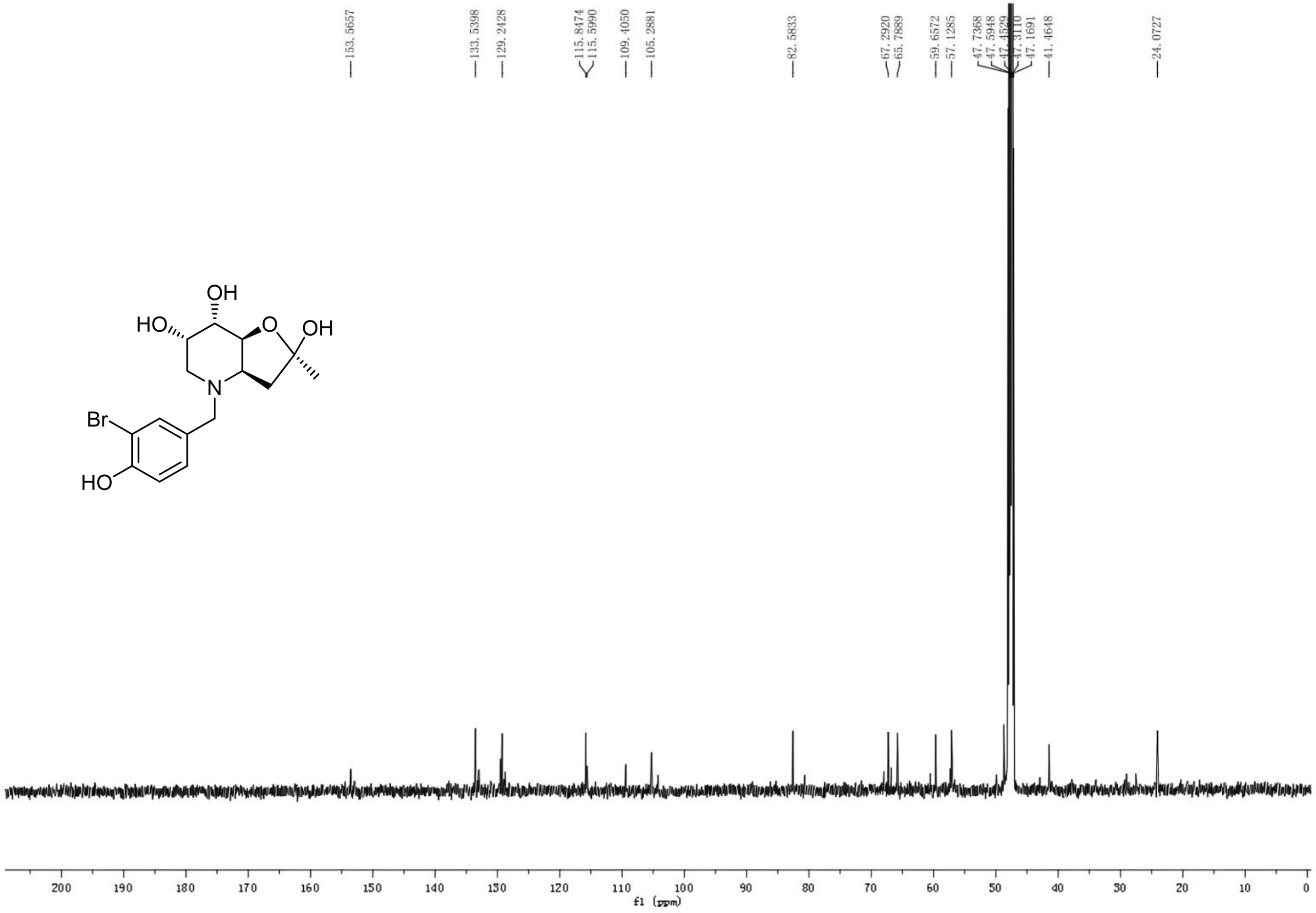

Figure S52. The ^{13}C-NMR of Compound **29**.

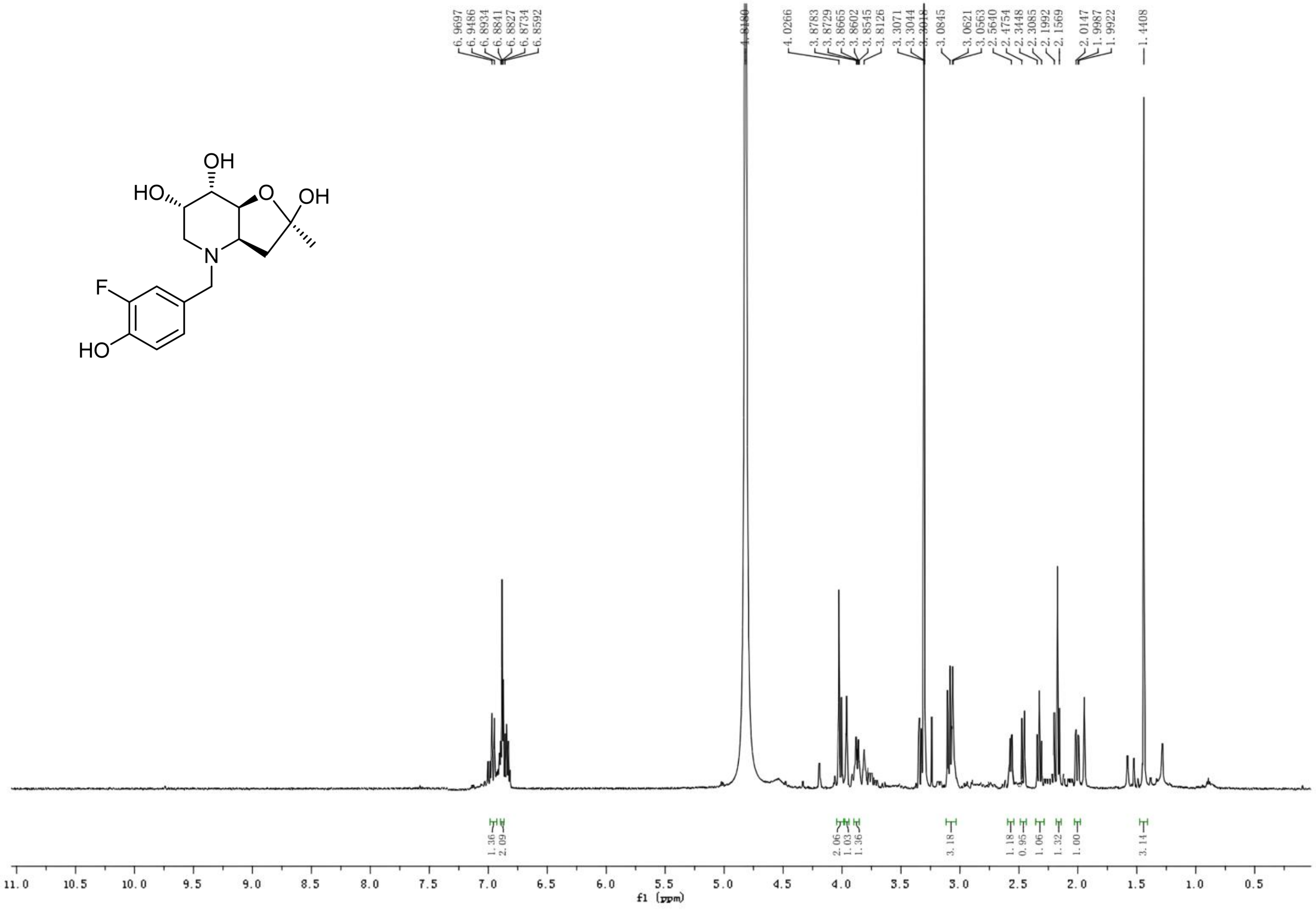

Figure S53. The ^{1}H NMR of compound **30**.

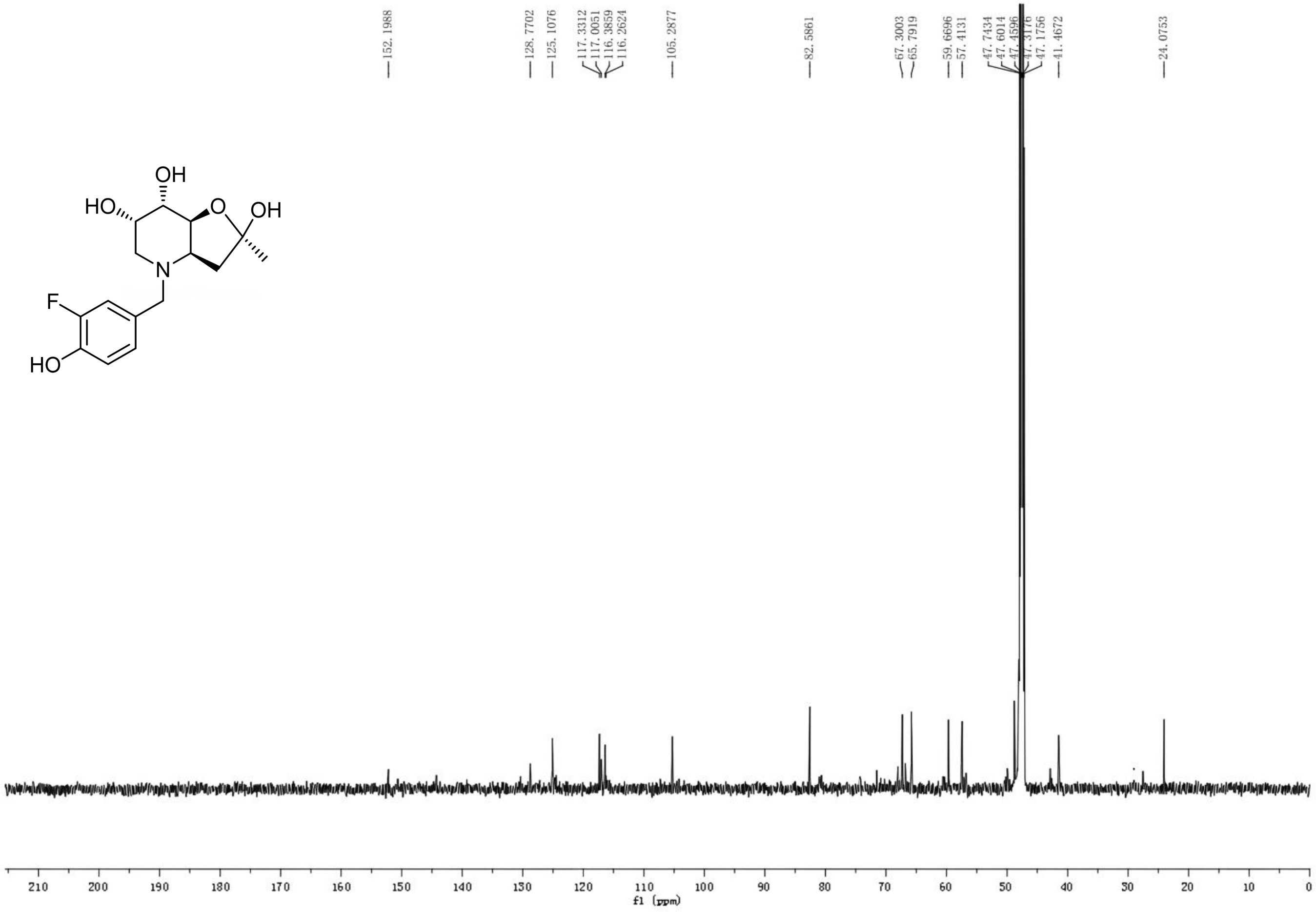

Figure S54. The ^{13}C-NMR of Compound **30**.

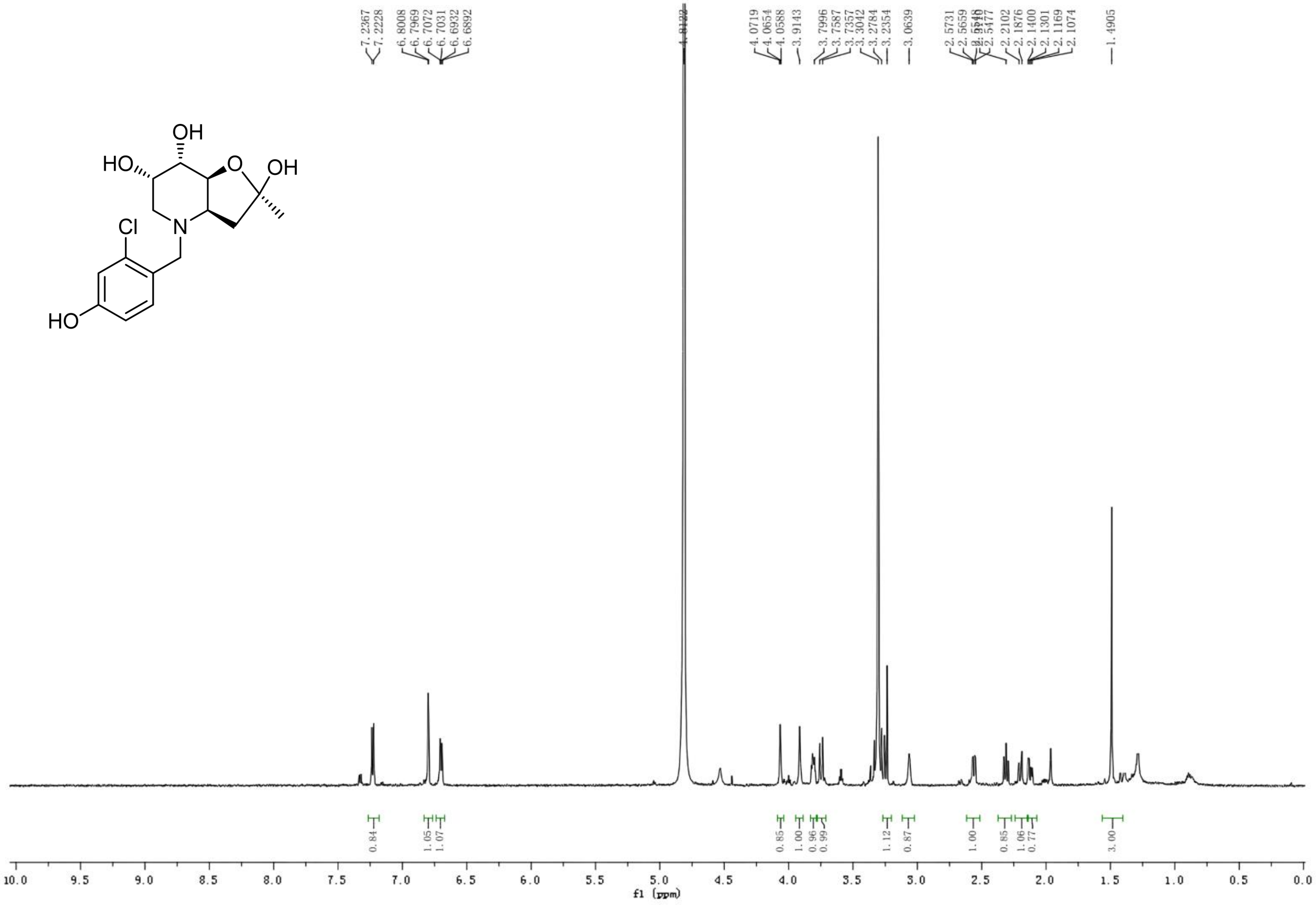

Figure S55. The ^{1}H NMR of compound **31**.

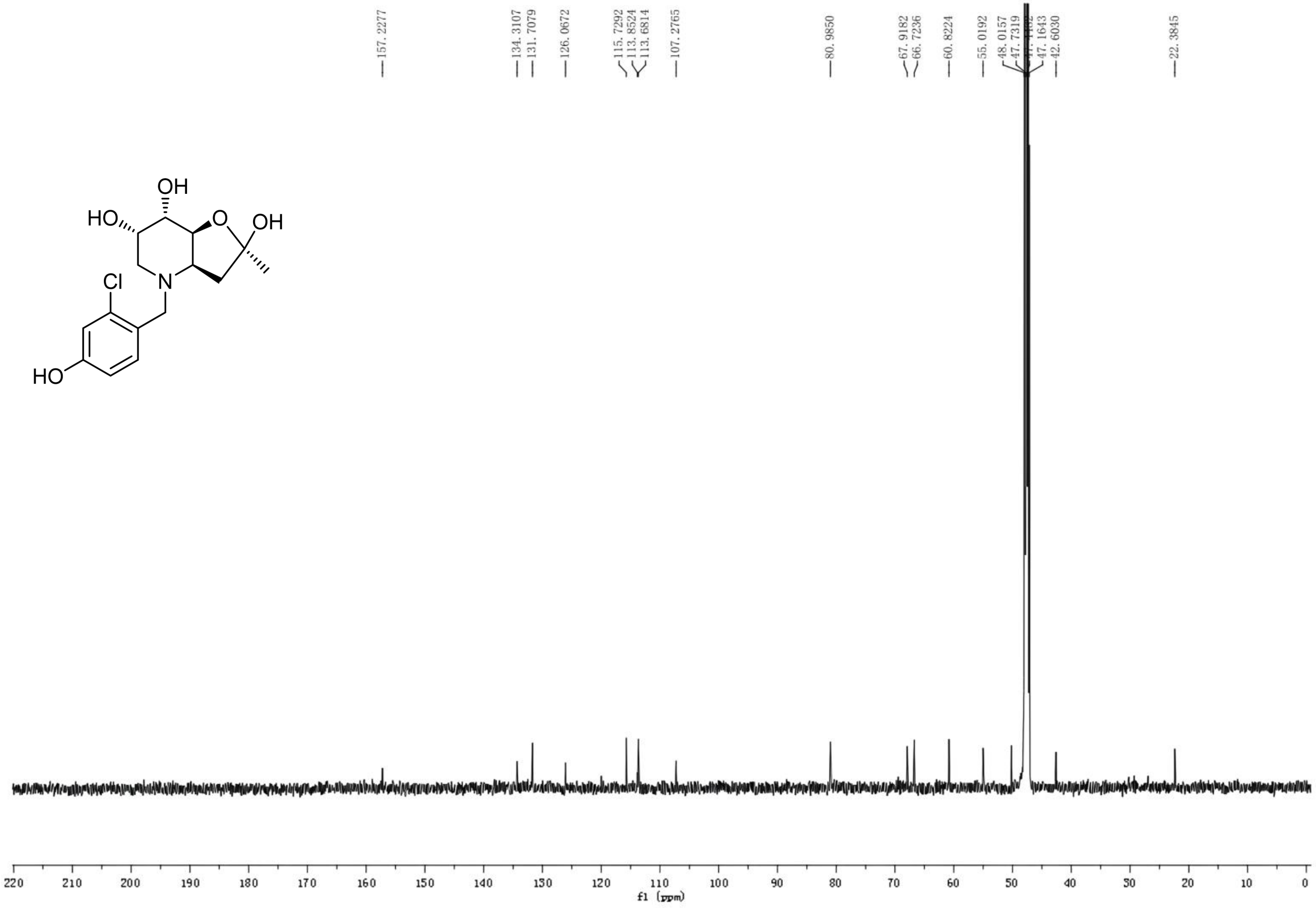

Figure S56. The ^{13}C-NMR of Compound **31**.

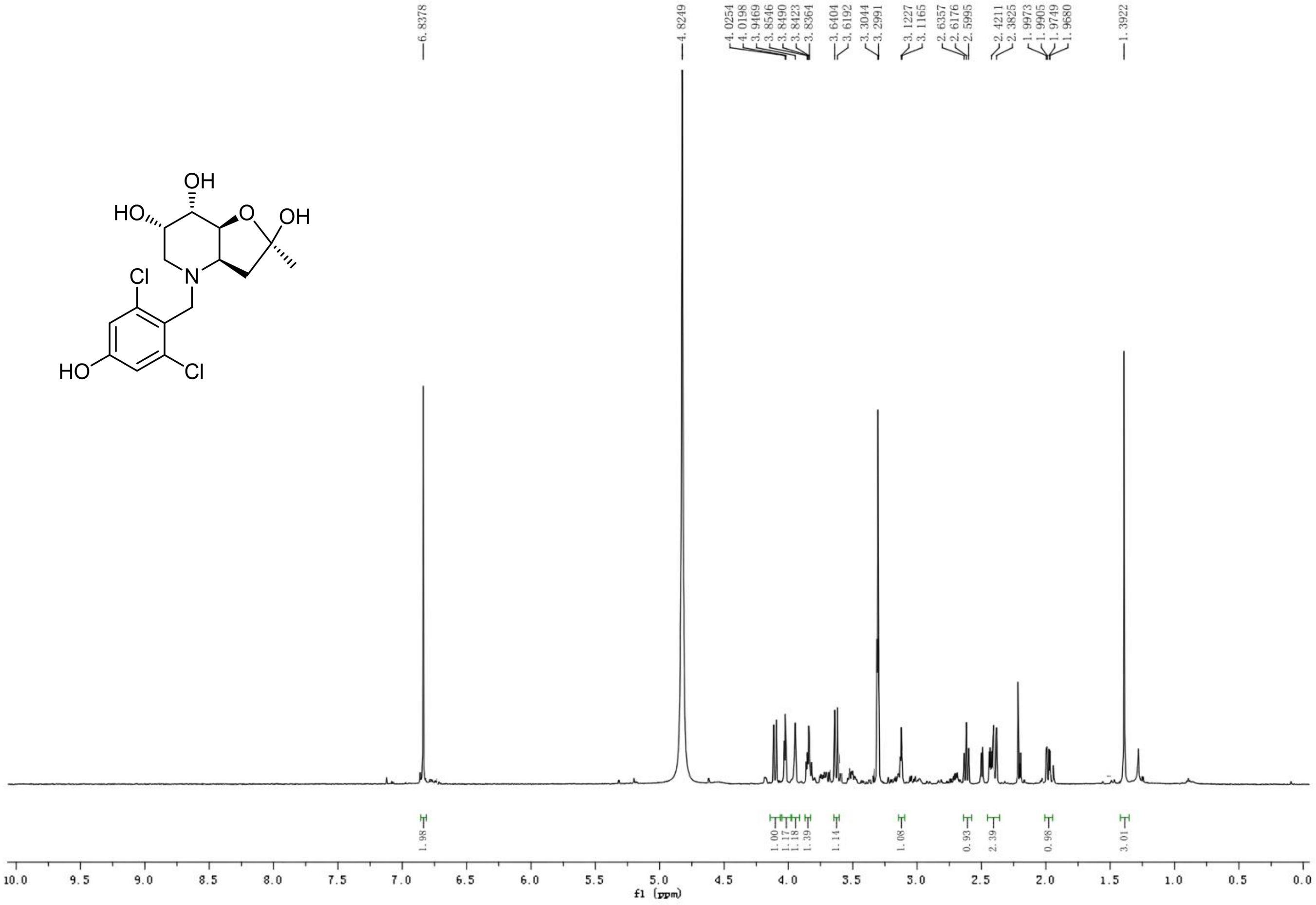

Figure S57. The ^{1}H NMR of compound **32**.

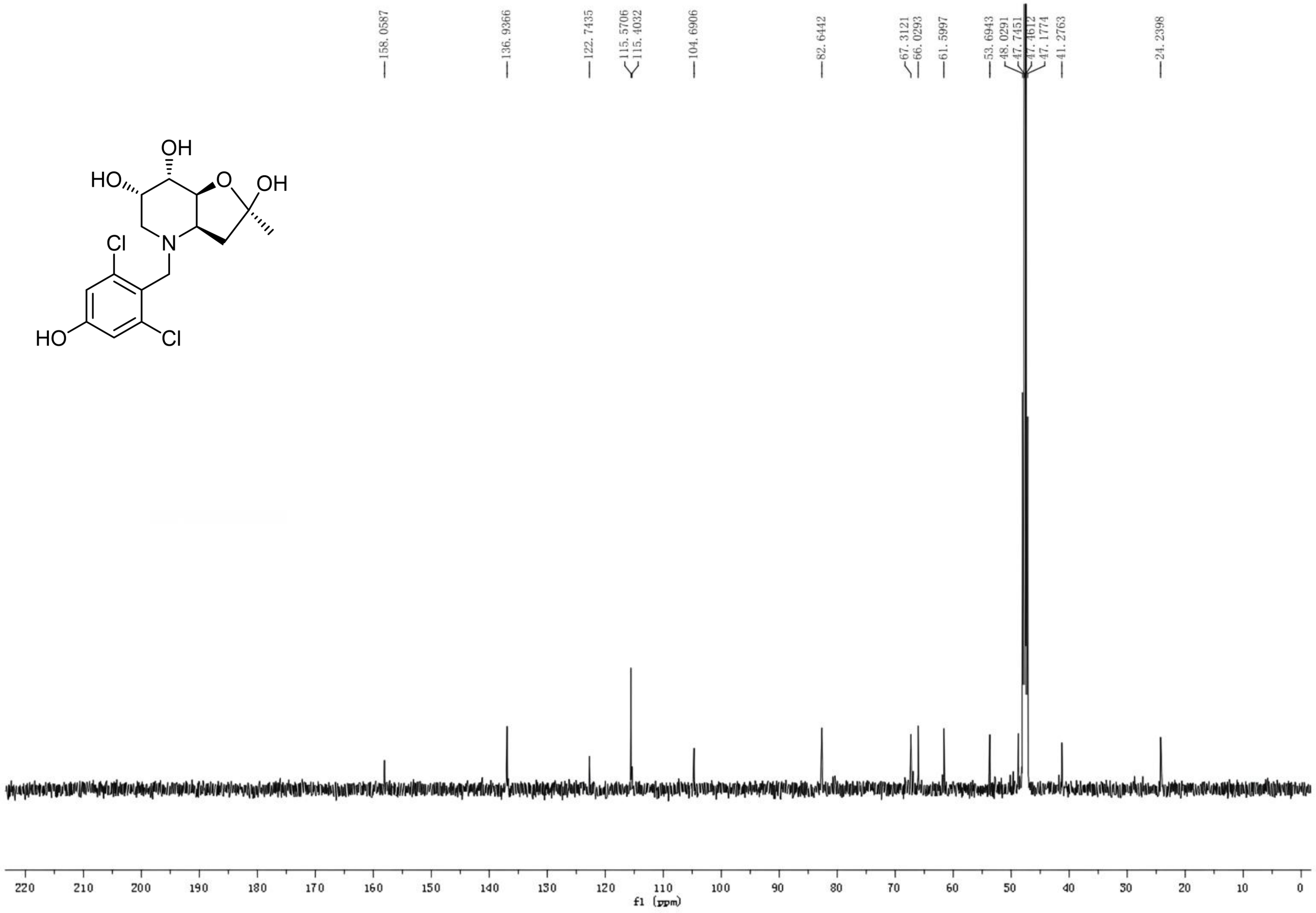

Figure S58. The ^{13}C-NMR of Compound **32**.

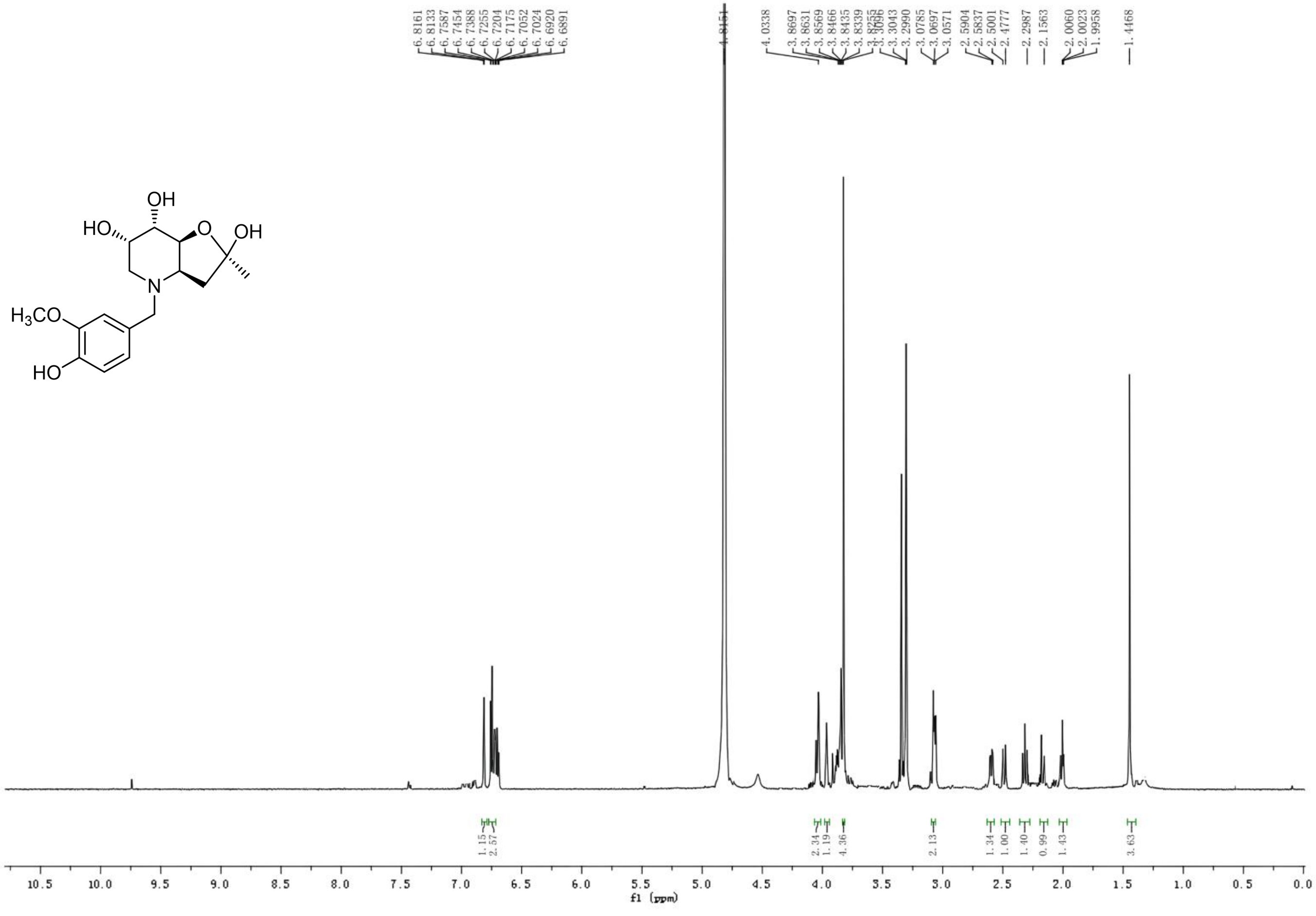

Figure S59. The ^{1}H NMR of compound **33**.

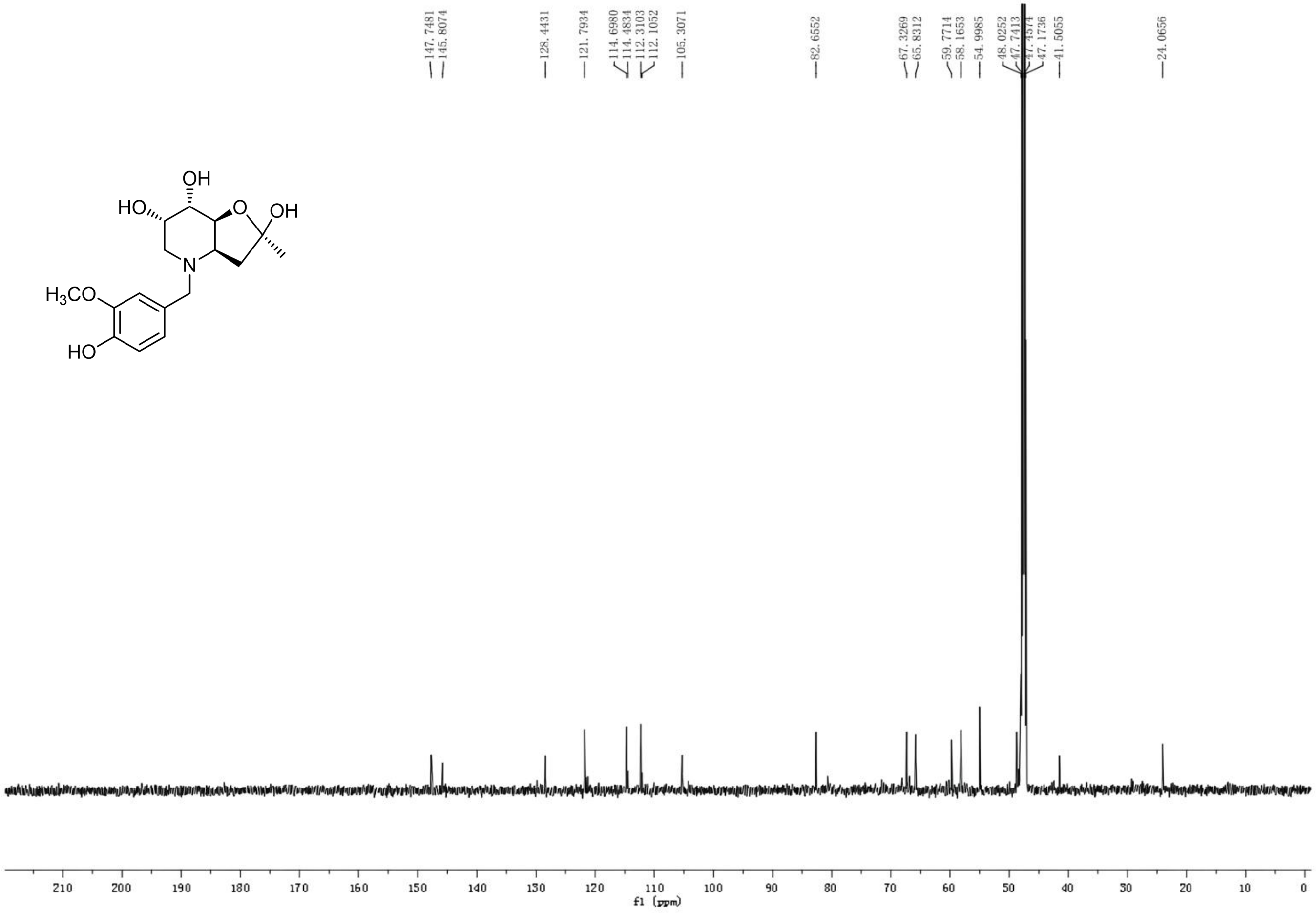

Figure S60. The ^{13}C-NMR of Compound **33**.

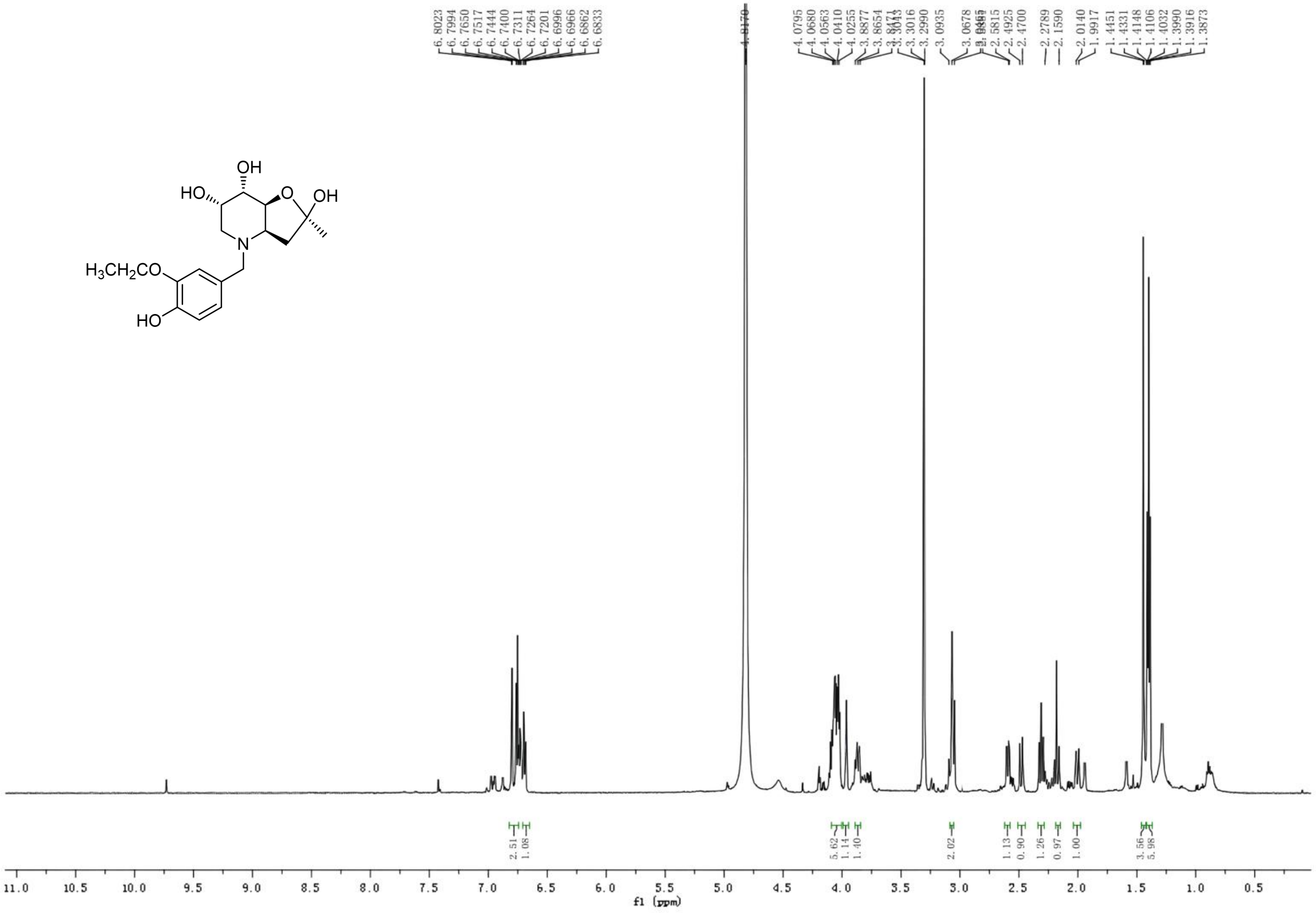

Figure S61. The ^{1}H NMR of compound **34**.

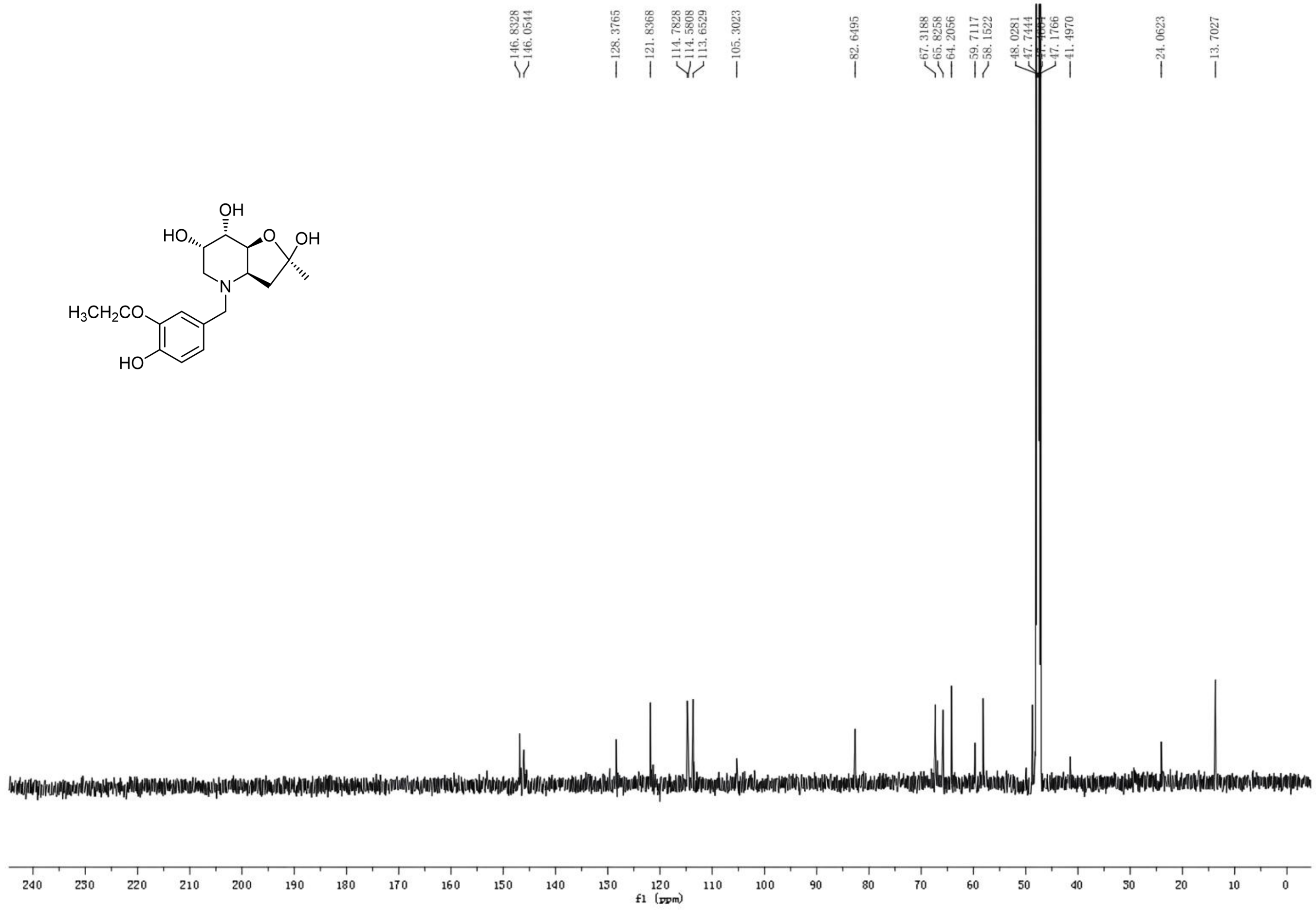

Figure S62. The ^{13}C-NMR of Compound **34**.
